# Global to local burden and inequalty of low respiratory infections among children and adolescents across 953 locations

**DOI:** 10.1186/s12889-025-25757-8

**Published:** 2025-11-25

**Authors:** Bo Yi, Yiran Huang, Aili Tian, Xu Hou, Longmei Yu, Queran Lin, Haiyun Zhang

**Affiliations:** 1 Dalian Women and Children’s Medical Group, Dalian, 116000 China; 2 WHO Collaborating Centre for Public Health Education and Training, School of Public Health, Department of Primary Care and Public Health, Faculty of Medicine, Imperial College London, London, UK; 3 No. 154 Zhongshan Road, Xigang District, Dalian City, 116000 Liaoning Province P. R. China; 4 Yixian Building, 107 Yanjiang Xi Road, Yuexiu District, Guangzhou City, Guangdong Province P. R. China

**Keywords:** LRIs, Inequality analysis, Frontier analysis, GBD

## Abstract

**Background:**

Adolescent lower respiratory infections (LRIs) are one of the major public health problems worldwide. Utilizing data from Global Burden of Disease 2021 (GBD 2021), this study aimed to investigate the trends and distribution characteristics of the burden of LRIs among children and adolescents from global to local levels, as well as to assess inequalities in disease burden. Unlike previous GBD reports, our analysis provides unprecedented subnational resolution across 953 global locations, uncovering local inequalities often masked in national-level estimates.

**Methods:**

We conducted an extensive analysis of the impact of LRIs across 953 global regions. This investigation included various metrics, including prevalence, incidence, mortality, disability-adjusted life years (DALYs), years of life lost (YLLs) and years lived with disability (YLDs). Additionally, we performed an inequality analysis and a frontier analysis pertinent to the sociodemographic index (SDI).

**Results:**

From 1990 to 2021, the incidence rate among adolescents decreased by 55.8%, and the DALYs rate decreased by 76.7%. Mortality rates decreased from 127.7 (95% uncertainty interval [UI]: 110.9 to 149.7) in 1980 to 21.2 (95% UI: 17.5 to 25.5) in 2021. In 2021, there were still 1.8 million adolescents worldwide suffering from LRIs. Among 652 local administrative regions, Mandera in Kenya had the highest incidence rate at 7,743.6 (95% UI: 6,876.8–8,715.6), and Sokoto in Nigeria had the highest DALYs rate at 16,663.3 (95% UI: 11,825.8–22,776.3). Since 1990, the global burden of LRIs among adolescents has significantly decreased, but regional disparities remain significant. From 1990 to 2021, the slope index of inequality (SII) for incidence rates and DALYs rates decreased by 56.8% and 81.5%, respectively, with higher disease burdens in low- and low- middle-SDI regions. In 2021, only 14.2% reached the incidence rate frontier level, and 15.2% reached the DALYs rate frontier level.

**Conclusions:**

Substantial progress has been made in reducing the burden of LRIs, but the burden remains heavy, particularly in low- and low- middle SDI regions. Trend analysis provides scientific evidence for international aid and policy-making to address inequalities.

**Supplementary Information:**

The online version contains supplementary material available at 10.1186/s12889-025-25757-8.

## Introduction

LRIs were the leading cause of death from infectious diseases in 2019, accounting for more than 2 million deaths annually worldwide [1], with particularly high mortality rates among children under five years of age [2]. LRIs can be caused by various pathogens, including bacteria, viruses, Mycoplasmas, and fungi. Streptococcus pneumoniae is among the most common pathogens associated with LRI incidence and mortality [3]. Respiratory syncytial virus (RSV) is a common pathogen in infants and young children with LRIs and one of the most common pathogens associated with hospitalization for pneumonia in children [3, 4]. Globally, it is closely associated with incidence and mortality in infants and young children, particularly in low- and middle-income countries [5–7].

Unlike upper respiratory tract infections, LRIs require active antibiotic treatment [8], significantly reducing long-term lung function in children and increasing the risk of developing asthma in children without a history of asthma [9]. Epidemiological studies have reported trends in the incidence, mortality, and pathogen composition of LRIs [2], as well as risk factors associated with different genders and ages [10]. Previous studies on the epidemiological trends and geographical distribution in adolescents and children have reported estimates only at the global level. Comparable estimates at levels ranging from global to local provinces are lacking, highlighting the need for more detailed research across these different scales.

This study integrates the largest spatiotemporal dataset on LRIs to date, covering 953 observation points from global to local levels between 1980 and 2021, to explore trends in incidence rates and DALYs among adolescents and children. Furthermore, we explored the correlation pattern with SDI through socioeconomic inequality analysis and frontier analysis to address the above issues. We also reported the distribution characteristics and inequality of the disease burden of LRIs among adolescents and children globally and in each region, with the aim of identifying the distribution of high-risk populations and areas of focus.

## Methods

### Data sources

The GBD 2021 report conducted a thorough examination of health losses attributable to 371 diseases and injuries across 204 countries and regions. This analysis specifically concentrated on three distinct age cohorts and the burden of LRIs across 953 national and subnational administrative divisions. The data pertaining to prevalence, incidence, mortality, DALYs, YLLs, and YLDs were obtained from the Institute for Health Metrics and Evaluation Global Health Data Exchange. The findings are articulated in terms of rates per 100,000 individuals, numbers and percentages, while being stratified by age, sex, and geographic location. This collective approach offers a detailed evaluation of the disease burden.

### Statistical modeling

In the GBD 2021 study, 75,459 data sources were used to calculate nonfatal indicators such as prevalence, incidence, DALYs, and YLDs, whereas 56,604 data sources were used to calculate fatality indicators such as mortality rates and YLLs from 1980 to 2021 [11]. DALYs are the sum of YLLs and YLDs [12]. YLLs are calculated via age-specific mortality rates and standard life expectancies, whereas YLDs are calculated using prevalence data and disability weights from the GBD 2021. The Bayesian meta-regression tool DisMod-MR 2.1 was used to estimate incidence rates and mortality rates, adjusting for covariates such as age, sex, and the SDI. 95% UI were generated through 1,000 posterior samples.

### Regional classification

The SDI is a composite indicator calculated based on socioeconomic status, average education level, and total fertility rate, categorizing 204 countries and regions into five levels: low, low-middle, middle, upper-middle, and high. In regional analysis, these regions are further divided into 21 regions based on geographical, epidemiological patterns, and cultural similarities [13]. This approach facilitates global disease burden comparison and analysis.

### Inequality analysis

The assessment of both absolute and relative disparities in the incidence rates of LRIs, as well as the corresponding DALYs rates, is conducted through the application of the SII and the concentration index (CI) [14]. The SII is derived from a regression analysis where incidence rates and DALYs rates are regressed against the SDI, followed by the computation of the midpoint value of the cumulative population distribution arranged by the SDI. Conversely, the CI is determined by aligning the cumulative incidence rate proportion with the cumulative population distribution across various SDI levels through the utilization of a Lorenz curve, followed by the numerical integration of the area beneath the curve.

### Frontier analysis

Frontier analysis combines local weighted regression (LOESS) and local polynomial regression to calculate a smooth curve [15]. This smooth curve represents the minimum achievable burden of LRI incidence rates and DALYs rates under current social development conditions. It is used to compare actual incidence rates and DALYs rates with the theoretical minimum values of the frontier level for each SDI, quantify the gap between different countries or regions and the theoretical level, and further understand the potential for improvement in incidence and disease burden at the national or regional level.

## Results

### Incidence rate of LRIs in 953 locations

**Fig. 1:**
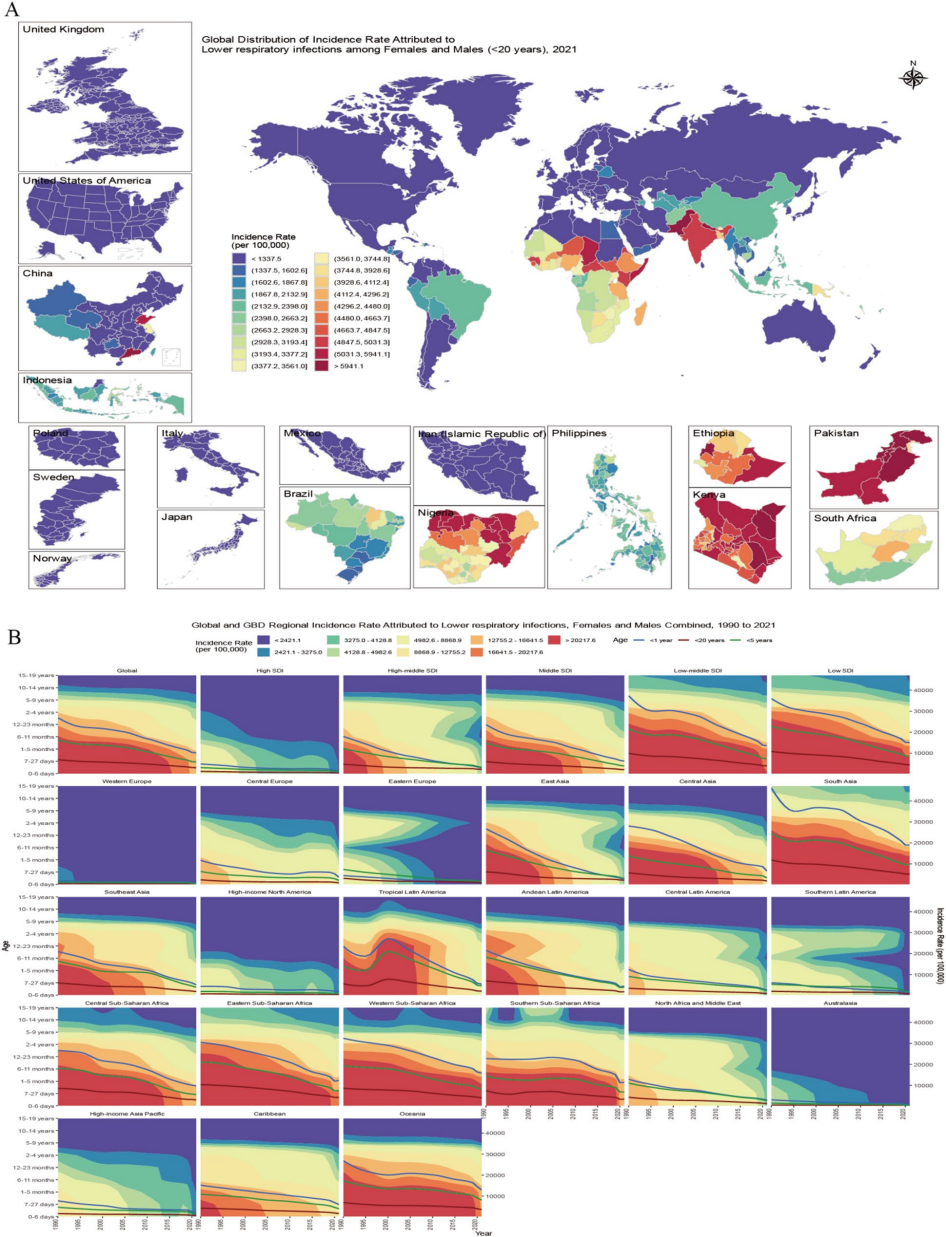
(**A**) Geographic distribution of < 20 years incidence rates in 2021. (**B**) The age-specific temporal patterns of incidence rates of LRIs in adolescents global and regional from 1990 to 2021. GBD, Global Burden of Disease; LRIs, lower respiratory infections; SDI, sociodemographic index

Based on the GBD data, the global incidence of LRIs showed a significant downward trend from 1990 to 2021. However, owing to the impact of the COVID-19 pandemic, the decline in incidence rates slowed from 2019 to 2021, and this phenomenon was particularly pronounced in low SDI and low-middle SDI regions (Fig. 1B). The incidence number for adolescents (< 20 years) decreased from 155,074,793 (95% UI: 139,519,396 to 173,367,472) to 80,077,892 (95% UI: 71,562,799 to 90,559,477), a decrease of 48.4% (95% UI: 50.6% to 46.0%). The incidence rate also decreased synchronously, with the incidence rate among adolescents in 2021 being 3,038.0 (95% UI: 2,715.0 to 3,435.7), a decrease of 56% (95% UI: 54% to 58%) compared with 6,866.0 (95% UI: 6,177.3 to 7,676.0) in 1990. and the incidence rate among infants (< 1 year) was 3–4 times higher than that among adolescents (Table 1).

**Table 1:** Global numbers and rates of LRIs incidence in different age groups

Age	Incidence number 1990	Incidence number 2019	Incidence number 2021	Incidence number 1990–2019	Incidence number 1990–2021	Incidence number 2019–2021
< 1 year	34,304,786 (31227655 to 37964062)	15,090,803 (13684936 to 16659169)	12,791,880 (11514160 to 14321358)	−56.0% (−61.6 to −49.5)	−62.7% (−64.1 to −61.2)	−15.2% (−18.0 to −12.2)
< 5 years	101,066,208 (89822531 to 113707337)	45,020,870 (40034721 to 50813610)	37,828,159 (33469076 to 43025157)	−55.5% (−62.3 to −47.4)	−62.6% (−64.0 to −61.2)	−16.0% (−18.4 to −13.2)
< 20 years	155,074,793 (139519396 to 173367472)	89,951,142 (80275133 to 101171494)	80,077,892 (71562799 to 90559477)	−42.0% (−50.4 to −32.1)	−48.4% (−50.6 to −46.0)	−11.0% (−13.1 to −8.8)
Age	Incidence rate 1990	Incidence rate 2019	Incidence rate 2021	Incidence rate 1990–2019	Incidence rate 1990–2021	Incidence rate 2019–2021
< 1 year	26853.6 (24444.8 to 29718.0)	11419.8 (10355.9 to 12606.6)	10096.6 (9088.1 to 11303.9)	−57.5% (−62.9 to −51.2)	−62.4% (−63.8 to −60.8)	−11.6% (−14.5 to −8.4)
< 5 years	16302.6 (14489.0 to 18341.7)	6639.0 (5903.8 to 7493.3)	5747.5 (5085.2 to 6537.1)	−59.3% (−65.5 to −51.9)	−64.7% (−66.1 to −63.5)	−13.4% (−15.9 to −10.6)
< 20 years	6866.0 (6177.3 to 7676.0)	3436.0 (3066.4 to 3864.6)	3038.0 (2715.0 to 3435.7)	−50.0% (−57.2 to −41.4)	−55.8% (−57.7 to −53.7)	−11.6% (−13.7 to −9.4)

Changes over time (1990–2019, 1990–2021, and 2019–2021) shown as percentage (95% uncertainty interval)

The global average incidence rate was 3,038.0 (95% UI: 2,883.2 to 3,651.2), with males having a higher overall incidence rate than females (males: 3,226.3; females: 2,837.8). However, among adolescents, females had higher incidence rates than males in 24 countries. Among these countries and regions, 4 with high SDIs accounted for 16.7%, 10 with high-middle SDIs accounted for 41.7%, 4 with middle SDIs accounted for 16.7%, and 2 with low-middle SDIs accounted for 8.3%. These countries and regions are primarily concentrated in North Africa and the Middle East. Among the top 100 countries by incidence rate, the top three regions are Western Sub-Saharan Africa, with the highest proportion (19%), where 89.5% of countries (17) have incidence rates above the global average; Oceania accounts for 18%, with only 11.1% of countries (2) exceeding the average; Eastern Sub-Saharan Africa accounts for 15%, with all countries (100.0%) exceeding the average (Fig. 1A); among SDI regions, low-middle SDI accounts for 34%, with 35.9% of countries (14) above average; low SDI accounts for 34%, with 82.4% of countries (28) above average. Among 204 countries and regions, Pakistan had the highest incidence rate at 6,612.4 (95% UI: 5,903.1 to 7,526.8), which decreased 51% from 1990 (95% UI: 47% to 54%). Among the 652 local administrative units, the countries with the highest proportion of units exceeding the global average incidence rate were Kenya (47 provinces, 100.0%) and Pakistan (7 provinces, 100.0%). Mandera in Kenya had the highest incidence rate among local administrative units at 7,743.6 (95% UI: 6,876.8 to 8,715.6).

### Trends in mortality and disability among LRIs in 953 locations

**Fig. 2:**
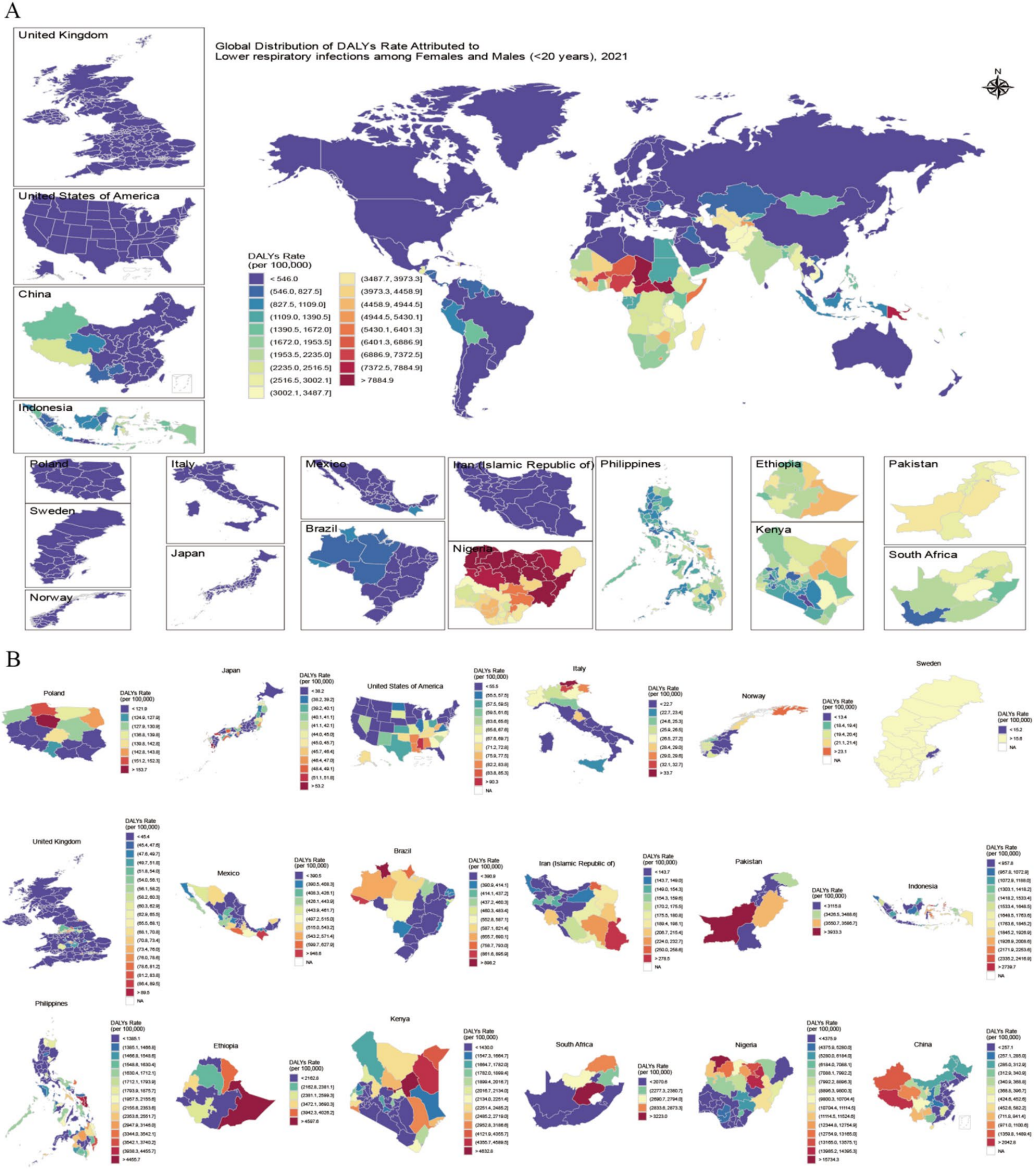
Geographic distribution of < 20 years DALYs rates in 2021 at national (**A**) and subnational levels (**B**). DALYs, disability-adjusted life years

In 2021, LRIs caused 559,877.3 (95% UI: 460,284.0 to 671,399.2) adolescent deaths worldwide. Compared with 1990, the mortality rate among adolescents decreased by 77% (95% UI: 72% to 80%) in 2021. The DALYs attributable to LRIs were less than one quarter of those reported in 1990, with the highest DALYs rate among infants, which was 2.3 times greater than that of adolescents. National-level data for 2021 revealed that Chad had the highest adolescent DALYs rate at 11,148.4 (95% UI:8,291.9 to 14,356.9) (Fig. 2A), while Niue and Tokelau reported increases of 94% (95% CI: 52% to 146%) and 49% (95% CI: 7% to 105%), respectively. In contrast, DALYs rates decreased in all other countries. Among the 652 local administrative units, Sokoto in Nigeria had the highest DALYs rate at 16,663.3 (95% UI: 11,825.8 to 22,776.3) (Fig. 2B), a decrease of 73% (95% UI: 61% to 81%) compared with 1990. The largest decrease in DALYs rate was observed in Lorestan, Iran, at 98% (95% UI: 97% to 99%). Compared with 1990, the global YLL rate decreased by 77% (95% UI: 72% to 80%) in 2021, and the YLD rate decreased by 55% (95% UI: 53% to 57%). Compared with other countries, Chad and South Sudan had higher YLL rates. Among local administrative units, Guizhou in China experienced the largest decrease in YLDs (Appendix).

### Trends in LRIs burden and regional inequalities

**Fig. 3:**
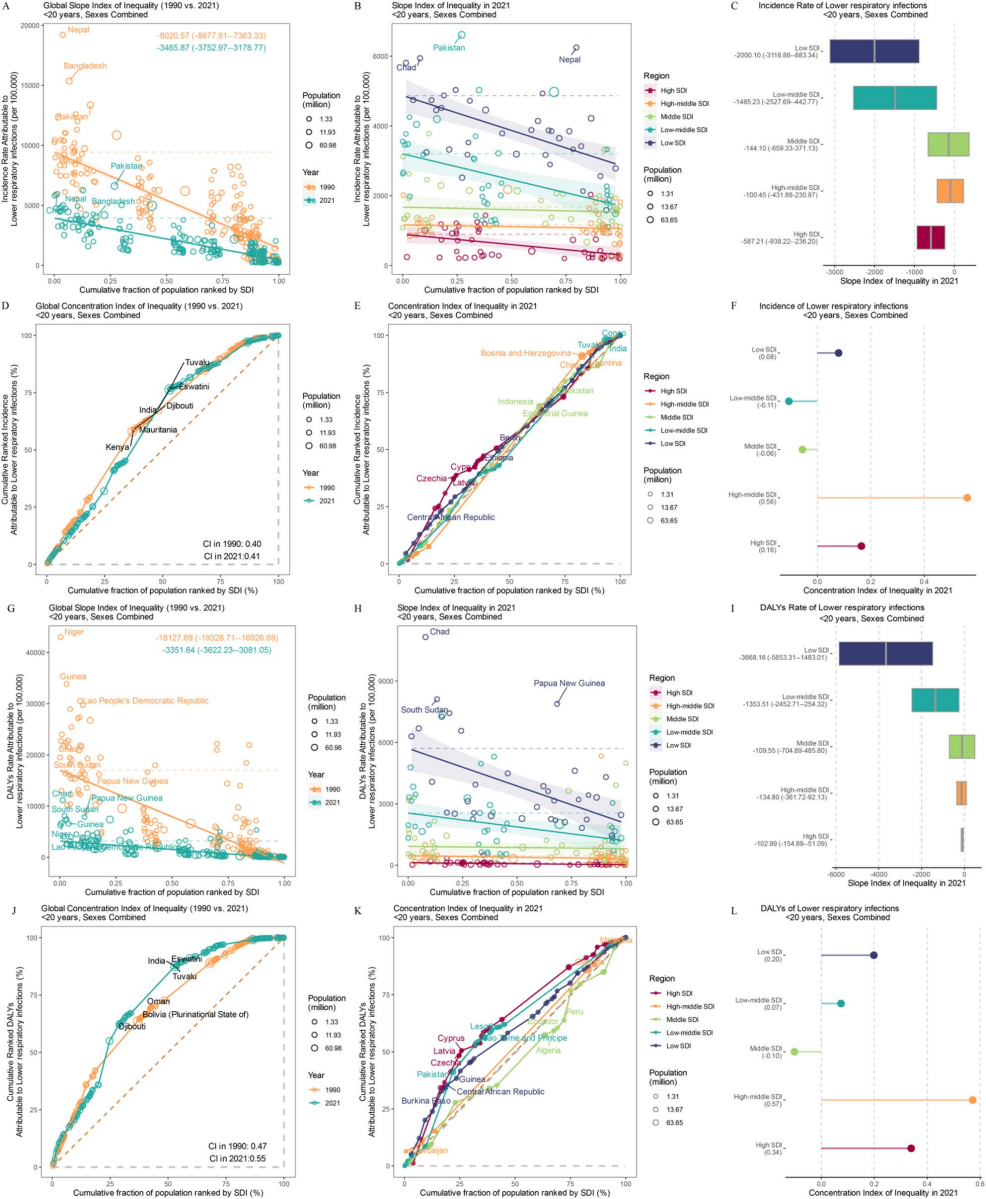
The absolute cross-country inequality in LRIs incidence rates and DALYs rates at global (**A**, **G**) and regional (**B**, **H**) levels, measured by the slope index of inequality (SII). Negative SII values imply that socioeconomically disadvantaged populations have higher burdens, whereas positive SII values imply the opposite.The relative cross-country inequality in LRIs incidence rates and DALYs rates at global (**D**, **J**) and regional (**F**, **K**) levels, measured by the concentration index (CI). Negative CI values imply that socioeconomically advantaged populations have higher burdens, whereas positive CI values imply the opposite. Quantitative forest plot of the SII for Incidence rate and DALYs rate(**C**, **I**).Quantitative forest plot of the CI for Incidence rate and DALYs rate (**F**,** L**)

Significant absolute and relative inequalities linked to the SDI have been observed globally. While there was a notable improvement in disease burden from 1990 to 2021, low- and low-middle SDI countries bore a greater burden (Fig. 3B, H). Compared with that 1990, the global incidence SII for adolescents decreased by 56.8% in 2021 (Fig. 3A), and the DALYs rate SII decreased by 81.5% between 1990 and 2021 (Fig. 3G). In 2021, the incidence SII for low and low-middle SDI regions were − 2000.10 (95% UI: −3116.86 to −883.34) and − 1,485.23 (95% UI: −2,527.69 to −442.77)(Fig. 3C), respectively. Pakistan, Nepal, and Chad were the three countries with the highest incidence rate inequality(Fig. 3B). The SII for DALYs rates in these two regions were − 3,668.16 (95% UI: −5,853.31 to −1,483.01) and − 1,353.51 (95% UI: −2,452.71 to −254.32), respectively (Fig. 3I). Chad, South Sudan and Papua New Guinea were the three countries with the highest DALYs rate inequality (Fig. 3H). Compared with 1990, the CI for DALYs increased by 17% in 2021 (Fig. 3J). Unlike absolute inequality, relative SDI-related inequality results revealed more severe regional inequality in medium-to-high SDI regions (Fig. 3F, L). Infants and children (< 5 years) exhibited the same trend (Appendix).

**Fig. 4:**
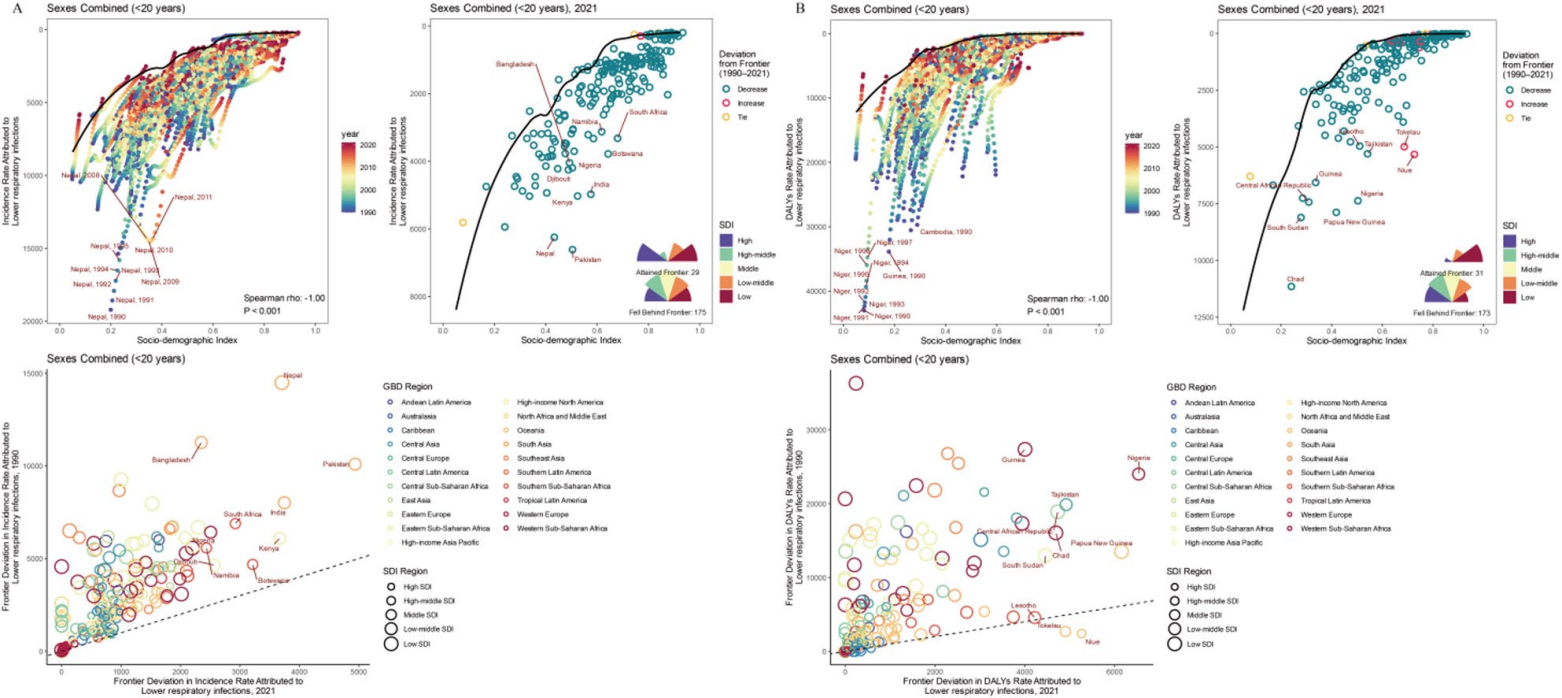
(**A**) Frontier analyses for LRIs incidence rates. The ‘frontier’ is the minimal incidence rate theoretically achievable at any SDI level and is shown as a solid black line in the top left and top right panels. The top left panel plots annual LRIs incidence rates for 204 countries (colored by year) to illustrate how each country’s rates compare with its frontier over time. The top right panel displays 204 countries’ incidence rates in 2021, coloured by whether the country’s gap from the frontier has increased (red) or decreased (green) since 1990. The bottom left panel compares frontier gaps for 1990 and 2021.(**B**)Frontier analyses for LRIs DALYs rates, following the same approach as in (**A**). DALYs, disability-adjusted life years; GBD, Global Burden of Disease; SDI, sociodemographic index

Frontier analysis of LRIs also revealed a negative correlation between SDI and disease burden (Fig. 4A, C). As SDI increases, the effective difference (EF) and variance decrease, indicating that as socioeconomic development progresses, there is increasing attention is given to the disease burden of LRIs. Between 1990 and 2021, most countries and regions approached the frontier level disease burden line in both the incidence rate and DALYs rate. However, in 2021, 85.8% of countries had not reached the incidence rate frontier level, and 84.8% had not reached the DALYs rate frontier level (Appendix). Pakistan, India, and Nepal had relatively high EFs in terms of incidence, whereas Nigeria, Papua New Guinea, and Niue had relatively high EFs in terms of DALYs (Fig. 4B, D). Nine countries showed an increase in EF for DALYs, all from middle and high-middle SDI regions. Spain showed an increase in EF for incidence.

## Discussion

Our study represents the most comprehensive analysis to date on LRIs in adolescents and children, boasting the widest geographical coverage and the longest observation period. The temporal and spatial distributions of the disease burden were further examined via inequality and frontier analysis. We have identified four key findings:

(1) The disease burden of LRIs among adolescents has decreased, whereas that among infants remains the heaviest. The global number of cases decreased from 155,074,793 in 1990 to 80,077,891.6 in 2021. In 2021, among 653 subnational regions, Guizhou Province in China experienced an 84.3% decrease in incidence rates, whereas Lorestan Province in Iran experienced a 98% decline in DALYs rates, making it the region with the fastest decline in disease burden. The DALYs rate for infants was 25,385.4 per 100,000 populations.
(2) Countries where the disease burden for women exceeds that for men are primarily concentrated in the Middle East and North Africa regions with high-middle and high SDI. There are 24 countries where the incidence rate for women is higher than for men, with only 8.3% of these countries located in low and low-middle SDI regions. There are 38 countries where the DALYs rate for women is higher than for men, with 21.1% of these countries located in low and low-middle SDI regions.
(3) Reduced inequality: Since 1990, DALY-related inequality has decreased by 81.5%. Chad, South Sudan, and Papua New Guinea were the three most unequal countries in 2021. Morbidity-related inequality decreased by 56.8%, with Pakistan, Nepal, and Chad ranking as the three countries with the highest morbidity-related inequality in 2021.
(4) Failure to reach frontier levels: In 2021, only 14.2% and 15.2% of countries reached the incidence rate and DALYs rate frontier level, respectively. As the SDI increases, the EF decreases, indicating that the disease burden of LRIs is increasingly being prioritized.

With economic development, the incidence of LRIs has been declining annually since 1990 [16]. However, in 2021, 559,877 adolescents still died from LRIs. In low and low-middle SDI regions, where social living conditions are poor and medical resources are limited, the COVID-19 pandemic in 2020 further diverted medical resources toward COVID-19 response, leading to a slower decline in incidence rates and even an increase in some countries, such as Namibia and Botswana. In high and high-middle SDI regions, widespread public health policies and nonpharmaceutical interventions, including mask wearing, travel restrictions, and remote work [17–20], not only limit the spread of the COVID-19 virus [18]but also significantly reduce the transmission of enveloped viruses such as RSV and influenza virus [17, 19, 20], thereby accelerating the decline in incidence rates in high and high-middle SDI countries. Infants and young children have underdeveloped immune and respiratory systems, resulting in high hospitalization rates and severe illness proportions due to RSV infection [5, 21, 22], leading to higher incidence rates and disease burden among this population. There was a trend toward a greater disease burden in males compared to females. Differences in respiratory system development between male and female children during childhood increase the susceptibility of males to early infections [23], and males also exhibit slower immune responses to pathogens, increasing their risk of severe LRIs [24]. Countries with higher disease burdens among males are primarily concentrated in low and low-middle SDI regions such as Western Sub-Saharan Africa [1, 10], whereas countries with higher disease burdens among females are concentrated in high-middle and high SDI regions in the Middle East and North Africa. These findings indicate that the distribution of LRIs disease burden is influenced not only by physiological factors but also by socioeconomic factors such as economic, cultural, and healthcare levels [25].

In 2021, the unequal burden of LRIs persisted, with significant regional disparities. Low- and low-middle income regions experience greater disease burden due to insufficient and misused medical resources, severe air pollution, overcrowded living conditions that accelerate pathogen transmission [26–28], and deficiencies in public health systems [10, 29, 30], which can also exacerbate the disease burden of LRIs. Political instability in regions such as sub-Saharan Africa and other conflict zones can further exacerbate the disease burden in LRIs [31]. Weak healthcare systems in these regions struggle to maintain stability during conflicts. As socioeconomic levels and infrastructure improve, the disease burden shifts toward high-income populations, revealing issues such as the uneven distribution of healthcare resources [32]and air pollution caused by urban industrialization in middle- and high-SDI countries [33].

Through frontier analysis, it was found that in low and low-middle SDI regions, countries such as Pakistan, India, Nepal, Nigeria, Papua New Guinea, and Niue are most likely to narrow the gap with frontier countries by optimizing international health technology support and resource allocation. It is also imperative to formulate public health policies and strengthen infrastructure construction. Additionally, establishing a comprehensive antibiotic management system to reduce antibiotic overuse [34, 35], implementing hospital infection control systems for LRI patients [36], and ensuring vaccine coverage for priority populations [10, 37]can further narrow the gap with leading countries. Despite potential limitations in the accuracy of data from low SDI regions, such data still holds considerable reference value.

Based on the aforementioned research, several practical and effective measures can be proposed that target the environment, public infrastructure development, and socioeconomic conditions. In terms of the environment, a core strategy involves reducing the use of biofuels to lower PM2.5 levels in the air. For countries with varying socioeconomic conditions, strategies can be tailored to their specific characteristics. Countries with lower socioeconomic conditions should prioritize public health and environmental disinfection, as well as the promotion of preventive measures, particularly in nations with scarce medical resources. Countries with better socioeconomic conditions, which typically have abundant medical resources, need to address issues related to the distribution of these resources. During the process of high-SDI urbanization, low-income populations may concentrate in crowded areas with relatively poor sanitary conditions, and medical resources should be appropriately allocated to these groups. Additionally, vaccination coverage should be expanded for different regions and age groups. Under the impact of COVID-19, some countries in low SDI regions have experienced a slowdown or even a rebound in the rate of decline. Postpandemic recovery strategies need to prioritize vulnerable regions.

### Limitations

Our dataset includes 953 observation points ranging from global to local levels, covering 204 countries, 21 GBD regions, and 652 subnational administrative units. It represents the most detailed regional division and longest time span worldwide to date. The report presents six core research indicators, providing numbers, rates, and 95% UI, thereby achieving a multidimensional quantification of health losses. Additionally, by integrating inequality and frontier analysis, the report quantifies trends in disease burden reduction and regional disparities, providing scientific evidence for international aid allocation and targeted policy formulation. Our study also has certain limitations. First, in low-SDI regions, poor healthcare standards lead to low cause-of-death attribution and diagnosis rates, incomplete information systems, and extremely low death registration rates, requiring extensive model imputation. Some countries experience conflict or political instability, further contributing to data incompleteness. Although DisMod-MR 2.1 can be adjusted, the uncertainty intervals remain widest in high-burden regions. Second, the study focused primarily on the association between SDI and disease burden but did not delve into other potential key factors, such as regional variations in the distribution of specific pathogens (e.g., Streptococcus pneumoniae, RSV) or the detailed impact of vaccination rates, which may limit a comprehensive understanding of the drivers of disease burden. Third, the report categorizes infants as < 1 year, children as < 5 years, and adolescents as < 20 years, without further refinement, which may overlook differing incidence trends among preschool-aged children and adolescents. Fourth, the frontier level of the minimum achievable burden in the frontier analysis report is derived from a model based on the socioeconomic level at the time. Its actual feasibility may be influenced by unquantified factors such as regional healthcare infrastructure and policy implementation capacity. Special circumstances such as the increase in DALYs rates in some countries are not adequately explained, limiting the reference value of the frontier level. Fifth, the explanation of gender differences is limited. While our study reported that countries with a greater disease burden among women are concentrated in high and upper-middle SDI regions, it did not further explore cultural, biological, and social factors, making it difficult to provide specific intervention pathways or policy implementation recommendations.

## Supplementary Information

Supplementary Material 1.

## Data Availability

The data used for analyses are publicly available at https://ghdx.healthdata.org/gbd-results-tool.

## Abbreviations

CI: Concentration Index
DALYs: Disability-Adjusted Life Years
DisMod-MR: Disease model-Bayesian meta-regression
EF: Effective Difference
GBD: Global Burden of Disease
LOESS: Local Weighted Regression
LRIs: Lower Respiratory Infections
SDI: Sociodemographic Index
SII: Slope Index of Inequality
UI: Uncertainty Interval
YLDs: Years Lived with Disability
YLLs: Years of Life Lost
