# Supplementary material for: Global to local burden and inequalty of low respiratory infections among children and adolescents across 953 locations
Source: BMC Public Health. 2025 Nov 25;26:35. doi: 10.1186/s12889-025-25757-8 (PMC12763975; doi:10.1186/s12889-025-25757-8)
Supplement: Supplementary file 1 — Supplementary Material 1. [file 12889_2025_25757_MOESM1_ESM.pdf]

Table of Contents

Inequality Analysis of incidence of Lower respiratory infections_1 year 2
Inequality Analysis of incidence of Lower respiratory infections_5 years 3
Inequality Analysis of incidence of Lower respiratory infections_20 years 4
Inequality Analysis of DALYs rate of Lower respiratory infections_1 year 5
Inequality Analysis of DALYs rate of Lower respiratory infections_5 years 6
Inequality Analysis of DALYs rate of Lower respiratory infections_20 years 7
Inequality Analysis of prevalence of Lower respiratory infections_1 year 8
Inequality Analysis of prevalence of Lower respiratory infections_5 years 9
Inequality Analysis of prevalence of Lower respiratory infections_20 years 10
Inequality Analysis of deaths rate of Lower respiratory infections_1 year 11
Inequality Analysis of deaths rate of Lower respiratory infections_5 years 12
Inequality Analysis of deaths rate of Lower respiratory infections_20 years 13
Frontier analysis 14

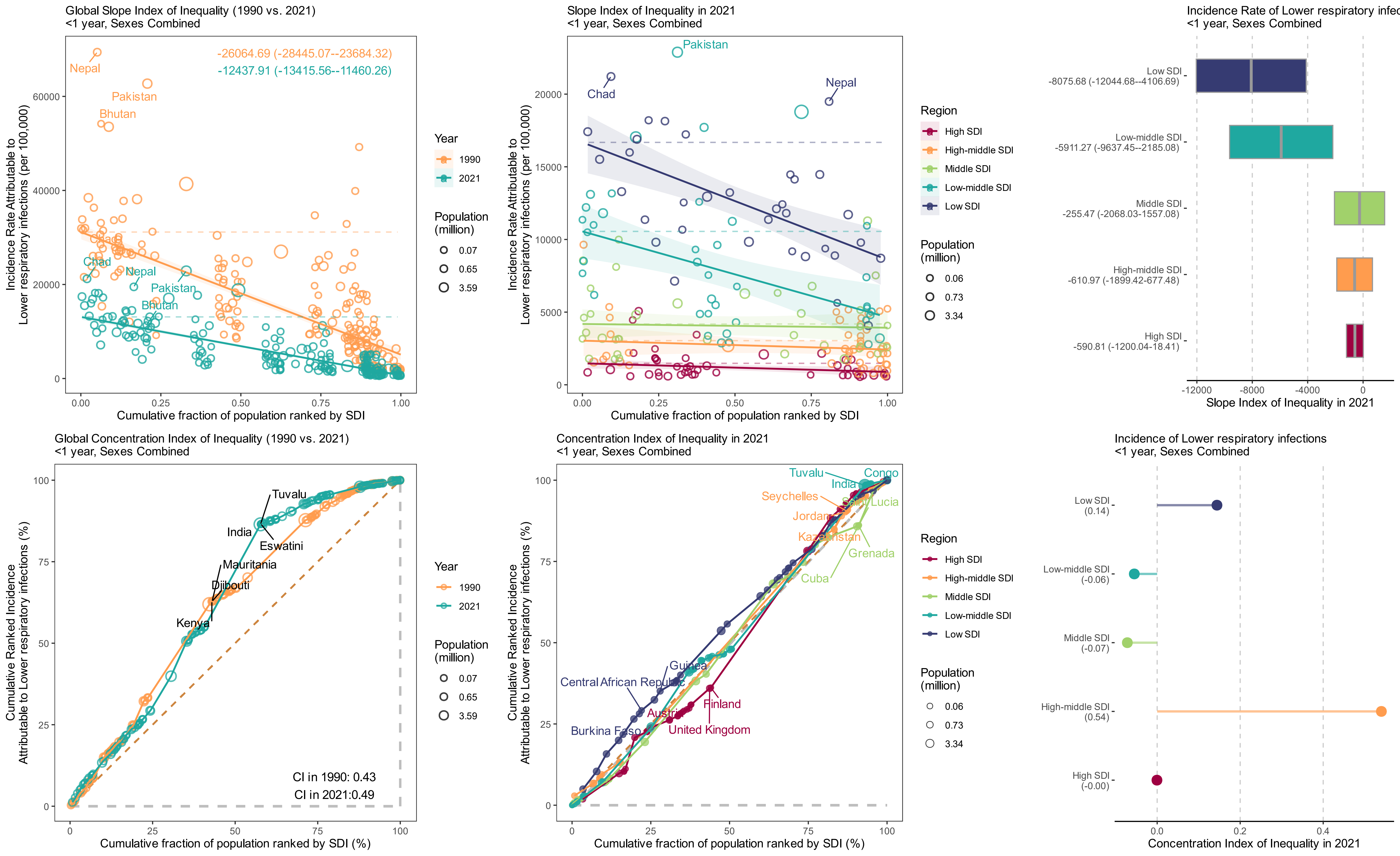

Global Slope Index of Inequality (1990 vs. 2021)
<1 year, Sexes Combined
-26064.69 (-28445.07--23684.32)
-12437.91 (-13415.56--11460.26)
Nepal
Pakistan
Bhutan
Chad
Nepal
Pakistan
Bhutan
Incidence Rate Attributable to Lower respiratory infections (per 100,000)
Cumulative fraction of population ranked by SDI
Year
1990
2021
Population (million)
0.07
0.65
3.59
Slope Index of Inequality in 2021
<1 year, Sexes Combined
Pakistan
Chad
Nepal
Region
High SDI
High-middle SDI
Middle SDI
Low-middle SDI
Low SDI
Population (million)
0.06
0.73
3.34
Incidence Rate of Lower respiratory infec
<1 year, Sexes Combined
Low SDI
-8075.68 (-12044.68--4106.69)
Low-middle SDI
-5911.27 (-9637.45--2185.08)
Middle SDI
-255.47 (-2068.03-1557.08)
High-middle SDI
-610.97 (-1899.42-677.48)
High SDI
-590.81 (-1200.04-18.41)
Slope Index of Inequality in 2021
Global Concentration Index of Inequality (1990 vs. 2021)
<1 year, Sexes Combined
Tuvalu
India
Eswatini
Mauritania
Djibouti
Kenya
CI in 1990: 0.43
CI in 2021:0.49
Cumulative Ranked Incidence Attributable to Lower respiratory infections (%)
Cumulative fraction of population ranked by SDI (%)
Concentration Index of Inequality in 2021
<1 year, Sexes Combined
Tuvalu
Congo
India
Seychelles
Saint Lucia
Jordan
Kazakhstan
Grenada
Cuba
Guinea
Central African Republic
Austria
Finland
Burkina Faso
United Kingdom
Incidence of Lower respiratory infections
<1 year, Sexes Combined
Low SDI (0.14)
Low-middle SDI (-0.06)
Middle SDI (-0.07)
High-middle SDI (0.54)
High SDI (-0.00)
Concentration Index of Inequality in 2021

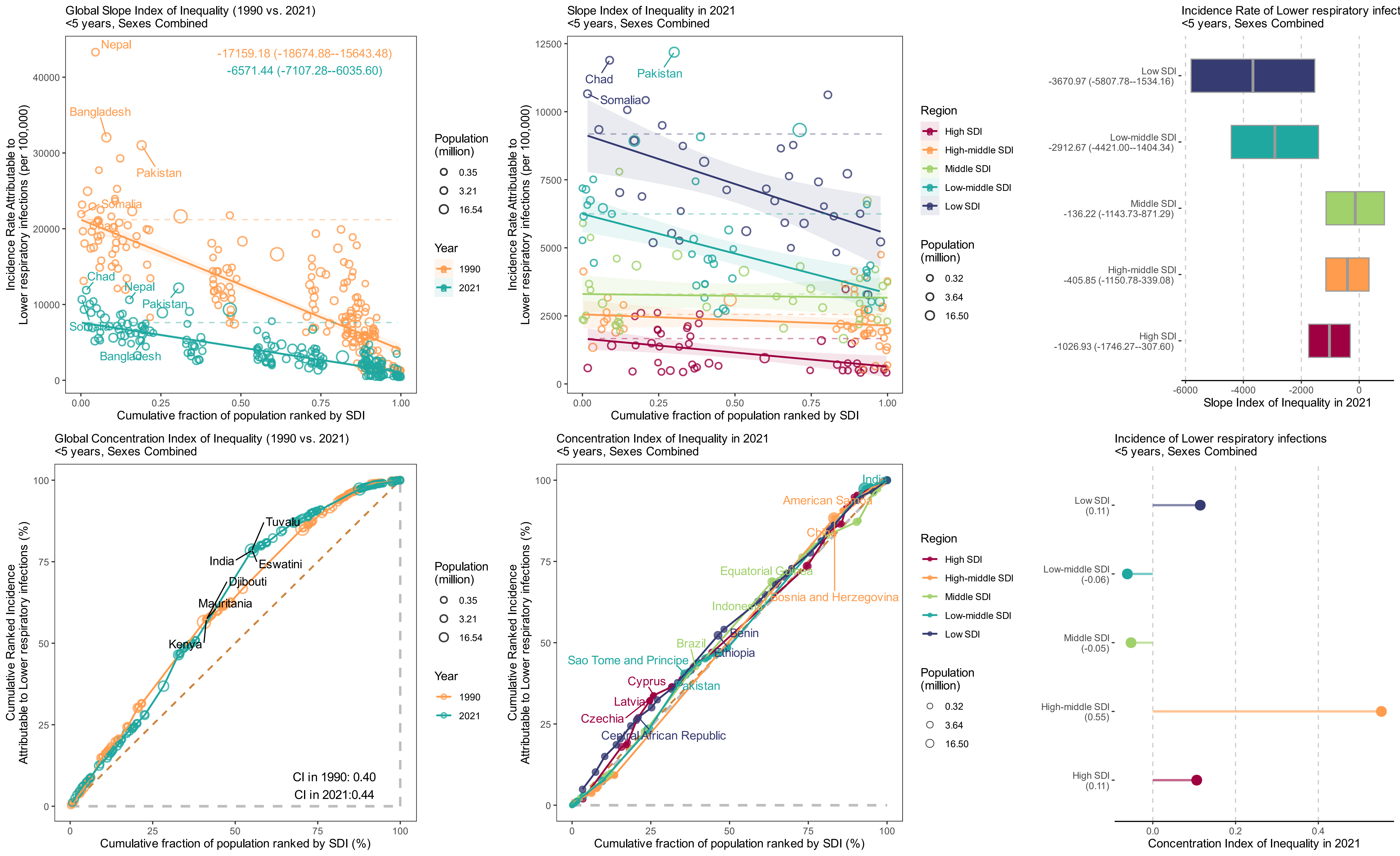
Global Slope Index of Inequality (1990 vs. 2021)
<5 years, Sexes Combined
-17159.18 (-18674.88--15643.48)
-6571.44 (-7107.28--6035.60)
Nepal
Bangladesh
Pakistan
Somalia
Chad
Incidence Rate Attributable to Lower respiratory infections (per 100,000)
Cumulative fraction of population ranked by SDI
Population (million)
0.35
3.21
16.54
Year
1990
2021
Slope Index of Inequality in 2021
<5 years, Sexes Combined
Chad
Pakistan
Somalia
Region
High SDI
High-middle SDI
Middle SDI
Low-middle SDI
Low SDI
Population (million)
0.32
3.64
16.50
Incidence Rate of Lower respiratory infect
<5 years, Sexes Combined
Low SDI
-3670.97 (-5807.78--1534.16)
Low-middle SDI
-2912.67 (-4421.00--1404.34)
Middle SDI
-136.22 (-1143.73-871.29)
High-middle SDI
-405.85 (-1150.78-339.08)
High SDI
-1026.93 (-1746.27--307.60)
Slope Index of Inequality in 2021
Global Concentration Index of Inequality (1990 vs. 2021)
<5 years, Sexes Combined
Tuvalu
India
Eswatini
Djibouti
Mauritania
Kenya
CI in 1990: 0.40
CI in 2021:0.44
Cumulative Ranked Incidence Attributable to Lower respiratory infections (%)
Cumulative fraction of population ranked by SDI (%)
Concentration Index of Inequality in 2021
<5 years, Sexes Combined
India
American Samoa
China
Equatorial Guinea
Bosnia and Herzegovina
Indonesia
Benin
Brazil
Ethiopia
Sao Tome and Principe
Cyprus
Pakistan
Latvia
Czechia
Central African Republic
Incidence of Lower respiratory infections
<5 years, Sexes Combined
Low SDI (0.11)
Low-middle SDI (-0.06)
Middle SDI (-0.05)
High-middle SDI (0.55)
High SDI (0.11)
Concentration Index of Inequality in 2021

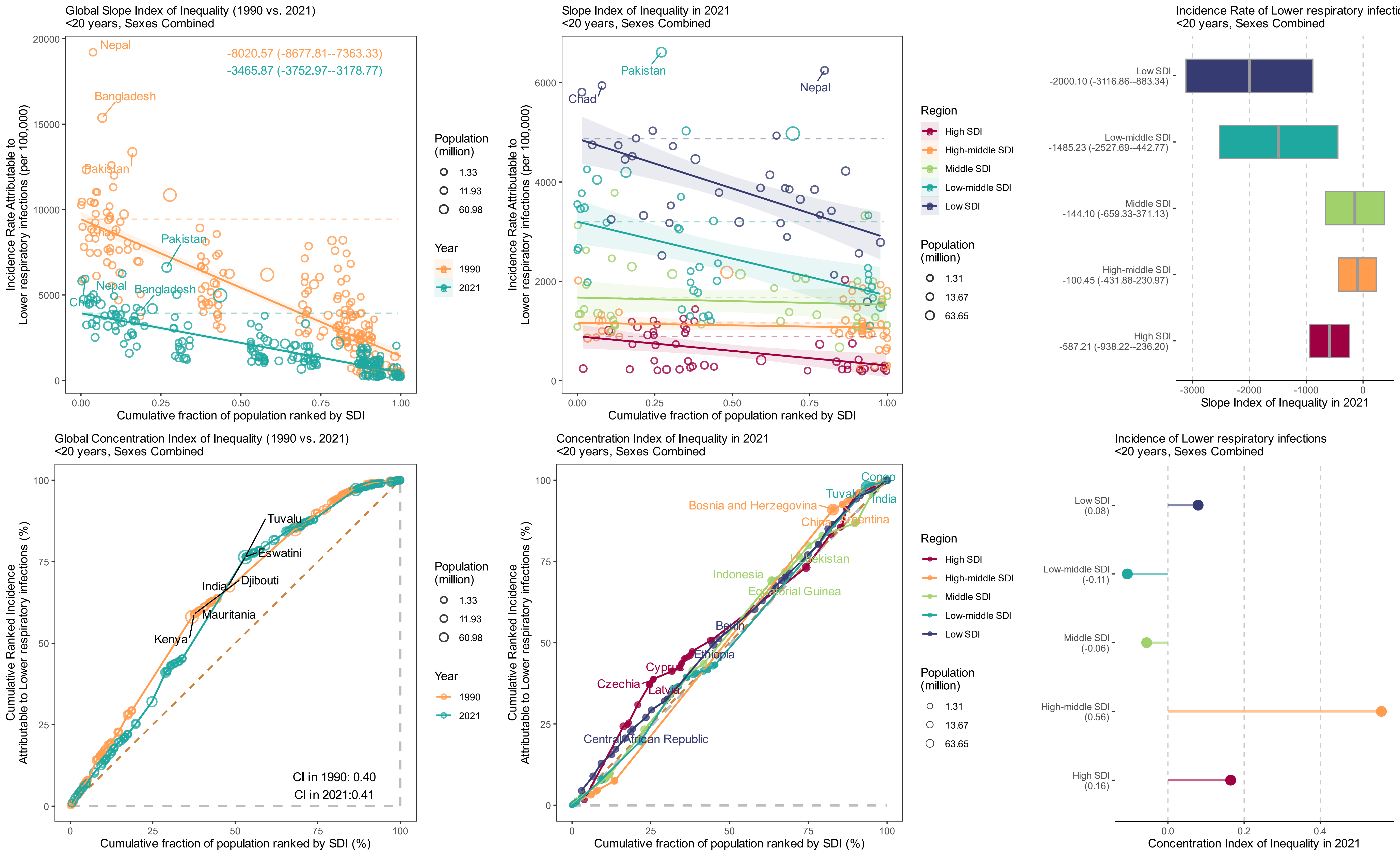

Global Slope Index of Inequality (1990 vs. 2021)
<20 years, Sexes Combined
-8020.57 (-8677.81--7363.33)
-3465.87 (-3752.97--3178.77)
Nepal
Bangladesh
Pakistan
Chad
Incidence Rate Attributable to Lower respiratory infections (per 100,000)
Cumulative fraction of population ranked by SDI
Population (million)
1.33
11.93
60.98
Year
1990
2021
Slope Index of Inequality in 2021
<20 years, Sexes Combined
Region
High SDI
High-middle SDI
Middle SDI
Low-middle SDI
Low SDI
Population (million)
1.31
13.67
63.65
Incidence Rate of Lower respiratory infecti
<20 years, Sexes Combined
Low SDI -2000.10 (-3116.86--883.34)
Low-middle SDI -1485.23 (-2527.69--442.77)
Middle SDI -144.10 (-659.33-371.13)
High-middle SDI -100.45 (-431.88-230.97)
High SDI -587.21 (-938.22--236.20)
Slope Index of Inequality in 2021
Global Concentration Index of Inequality (1990 vs. 2021)
<20 years, Sexes Combined
Tuvalu
Eswatini
India
Djibouti
Mauritania
Kenya
CI in 1990: 0.40
CI in 2021:0.41
Cumulative Ranked Incidence Attributable to Lower respiratory infections (%)
Cumulative fraction of population ranked by SDI (%)
Concentration Index of Inequality in 2021
<20 years, Sexes Combined
Congo
Tuvalu
India
Bosnia and Herzegovina
China
Argentina
Uzbekistan
Indonesia
Equatorial Guinea
Benin
Ethiopia
Cyprus
Czechia
Latvia
Central African Republic
Incidence of Lower respiratory infections
<20 years, Sexes Combined
Low SDI (0.08)
Low-middle SDI (-0.11)
Middle SDI (-0.06)
High-middle SDI (0.56)
High SDI (0.16)
Concentration Index of Inequality in 2021

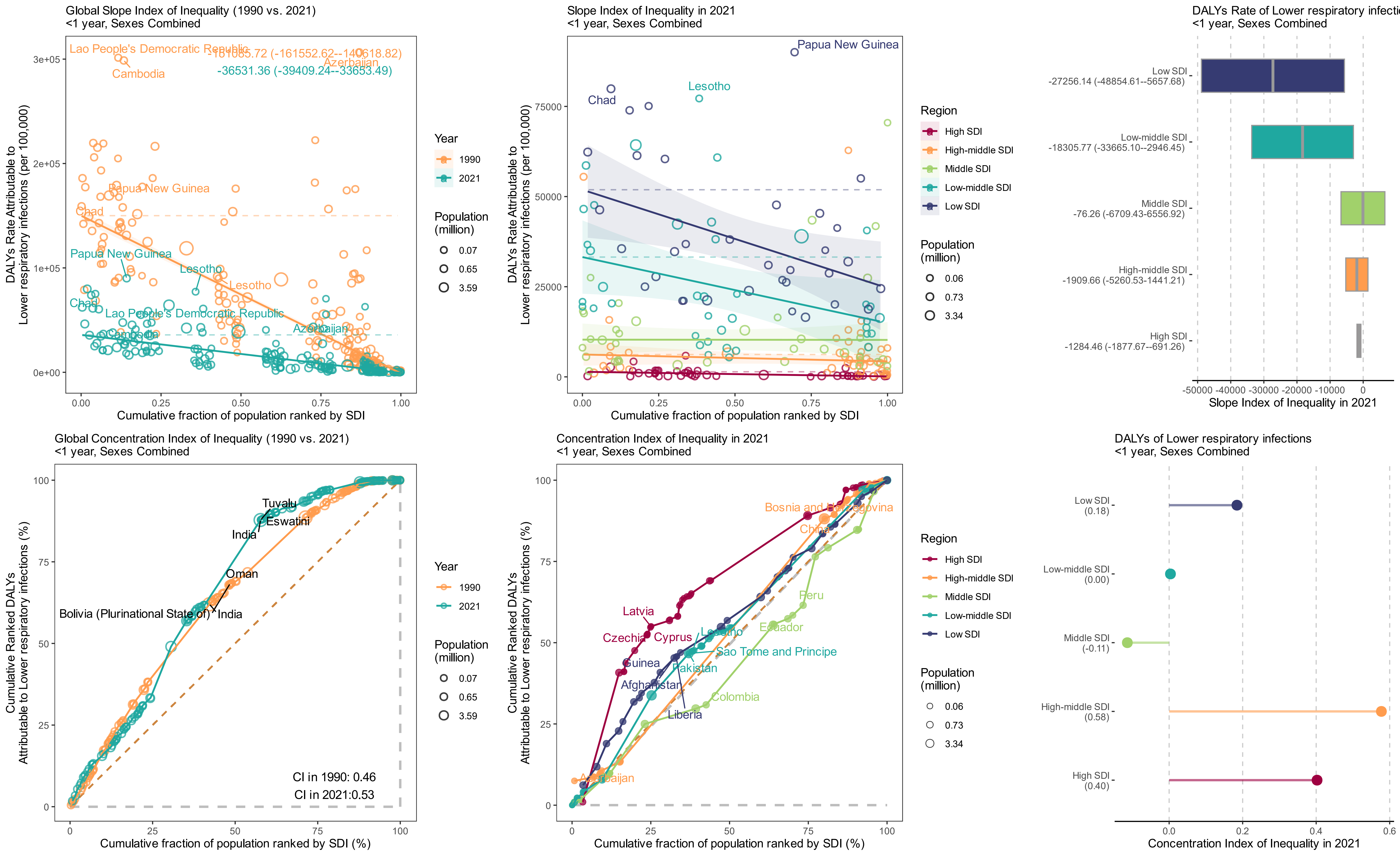
Global Slope Index of Inequality (1990 vs. 2021)
<1 year, Sexes Combined
DALYs Rate Attributable to Lower respiratory infections (per 100,000)
Cumulative fraction of population ranked by SDI
-36531.36 (-39409.24--33653.49)
Lao People's Democratic Republic
Cambodia
Papua New Guinea
Chad
Lesotho
Azerbaijan
Year
1990
2021
Population (million)
0.07
0.65
3.59
Slope Index of Inequality in 2021
<1 year, Sexes Combined
Region
High SDI
High-middle SDI
Middle SDI
Low-middle SDI
Low SDI
0.06
0.73
3.34
DALYs Rate of Lower respiratory infecti
<1 year, Sexes Combined
Low SDI
-27256.14 (-48854.61--5657.68)
Low-middle SDI
-18305.77 (-33665.10--2946.45)
Middle SDI
-76.26 (-6709.43-6556.92)
High-middle SDI
-1909.66 (-5260.53-1441.21)
High SDI
-1284.46 (-1877.67--691.26)
Slope Index of Inequality in 2021
Global Concentration Index of Inequality (1990 vs. 2021)
<1 year, Sexes Combined
Cumulative Ranked DALYs Attributable to Lower respiratory infections (%)
Cumulative fraction of population ranked by SDI (%)
Tuvalu
Eswatini
India
Oman
Bolivia (Plurinational State of)
CI in 1990: 0.46
CI in 2021:0.53
Concentration Index of Inequality in 2021
<1 year, Sexes Combined
Bosnia and Herzegovina
China
Peru
Ecuador
Latvia
Czechia
Cyprus
Sao Tome and Principe
Guinea
Pakistan
Afghanistan
Colombia
Liberia
DALYs of Lower respiratory infections
<1 year, Sexes Combined
Low SDI (0.18)
Low-middle SDI (0.00)
Middle SDI (-0.11)
High-middle SDI (0.58)
High SDI (0.40)
Concentration Index of Inequality in 2021

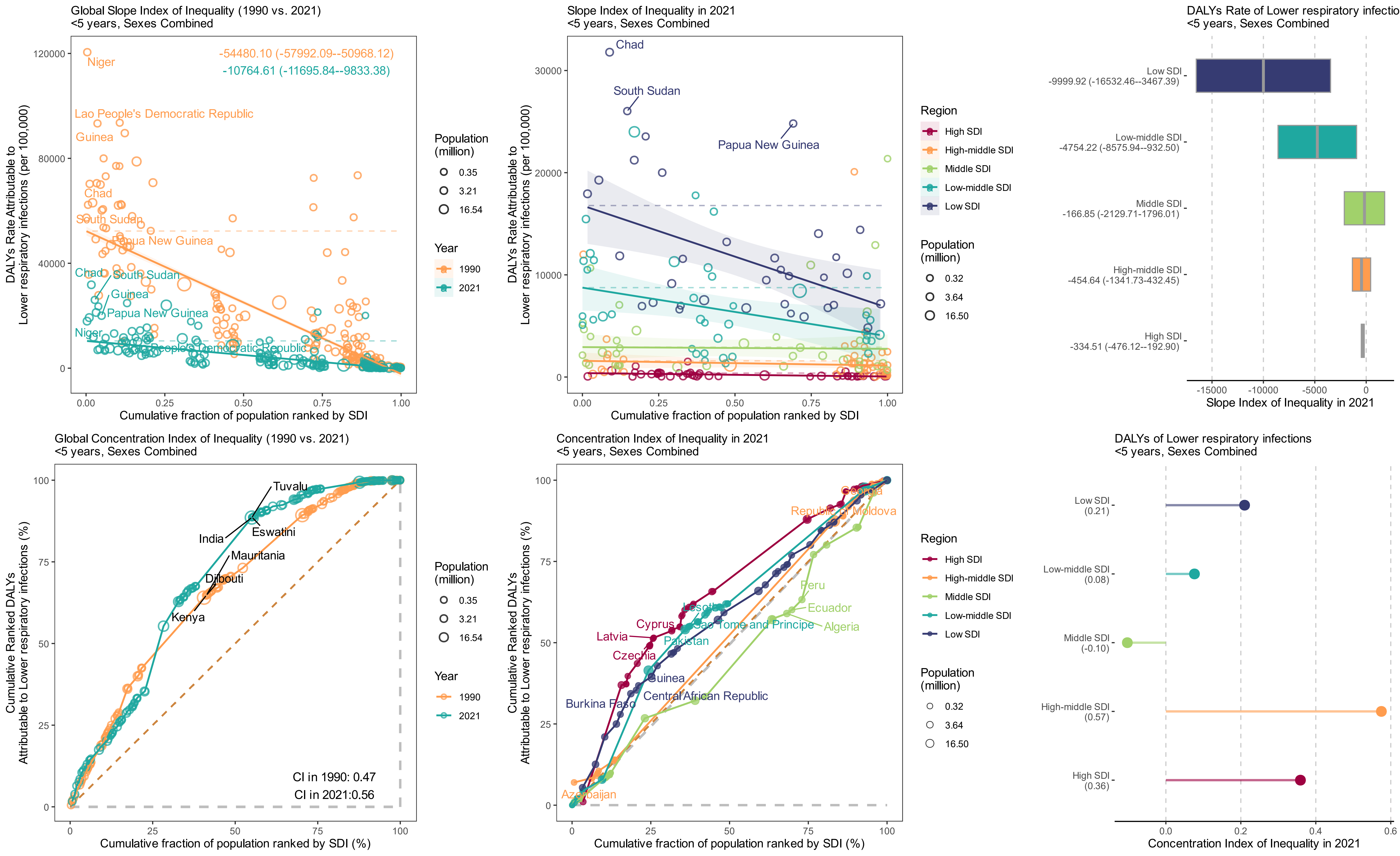
Global Slope Index of Inequality (1990 vs. 2021)
<5 years, Sexes Combined
-54480.10 (-57992.09--50968.12)
-10764.61 (-11695.84--9833.38)
Niger
Lao People's Democratic Republic
Guinea
Chad
South Sudan
Papua New Guinea
DALYs Rate Attributable to Lower respiratory infections (per 100,000)
Cumulative fraction of population ranked by SDI
Population (million)
0.35
3.21
16.54
Year
1990
2021
Slope Index of Inequality in 2021
<5 years, Sexes Combined
Chad
South Sudan
Papua New Guinea
Region
High SDI
High-middle SDI
Middle SDI
Low-middle SDI
Low SDI
Population (million)
0.32
3.64
16.50
DALYs Rate of Lower respiratory infectio
<5 years, Sexes Combined
Low SDI
-9999.92 (-16532.46--3467.39)
Low-middle SDI
-4754.22 (-8575.94--932.50)
Middle SDI
-166.85 (-2129.71-1796.01)
High-middle SDI
-454.64 (-1341.73-432.45)
High SDI
-334.51 (-476.12--192.90)
Slope Index of Inequality in 2021
Global Concentration Index of Inequality (1990 vs. 2021)
<5 years, Sexes Combined
Tuvalu
India
Eswatini
Mauritania
Djibouti
Kenya
CI in 1990: 0.47
CI in 2021:0.56
Cumulative Ranked DALYs Attributable to Lower respiratory infections (%)
Cumulative fraction of population ranked by SDI (%)
Concentration Index of Inequality in 2021
<5 years, Sexes Combined
Georgia
Republic of Moldova
Peru
Ecuador
Algeria
Cyprus
Lesotho
Sao Tome and Principe
Latvia
Pakistan
Czechia
Guinea
Central African Republic
Burkina Faso
Azerbaijan
DALYs of Lower respiratory infections
<5 years, Sexes Combined
Low SDI (0.21)
Low-middle SDI (0.08)
Middle SDI (-0.10)
High-middle SDI (0.57)
High SDI (0.36)
Concentration Index of Inequality in 2021

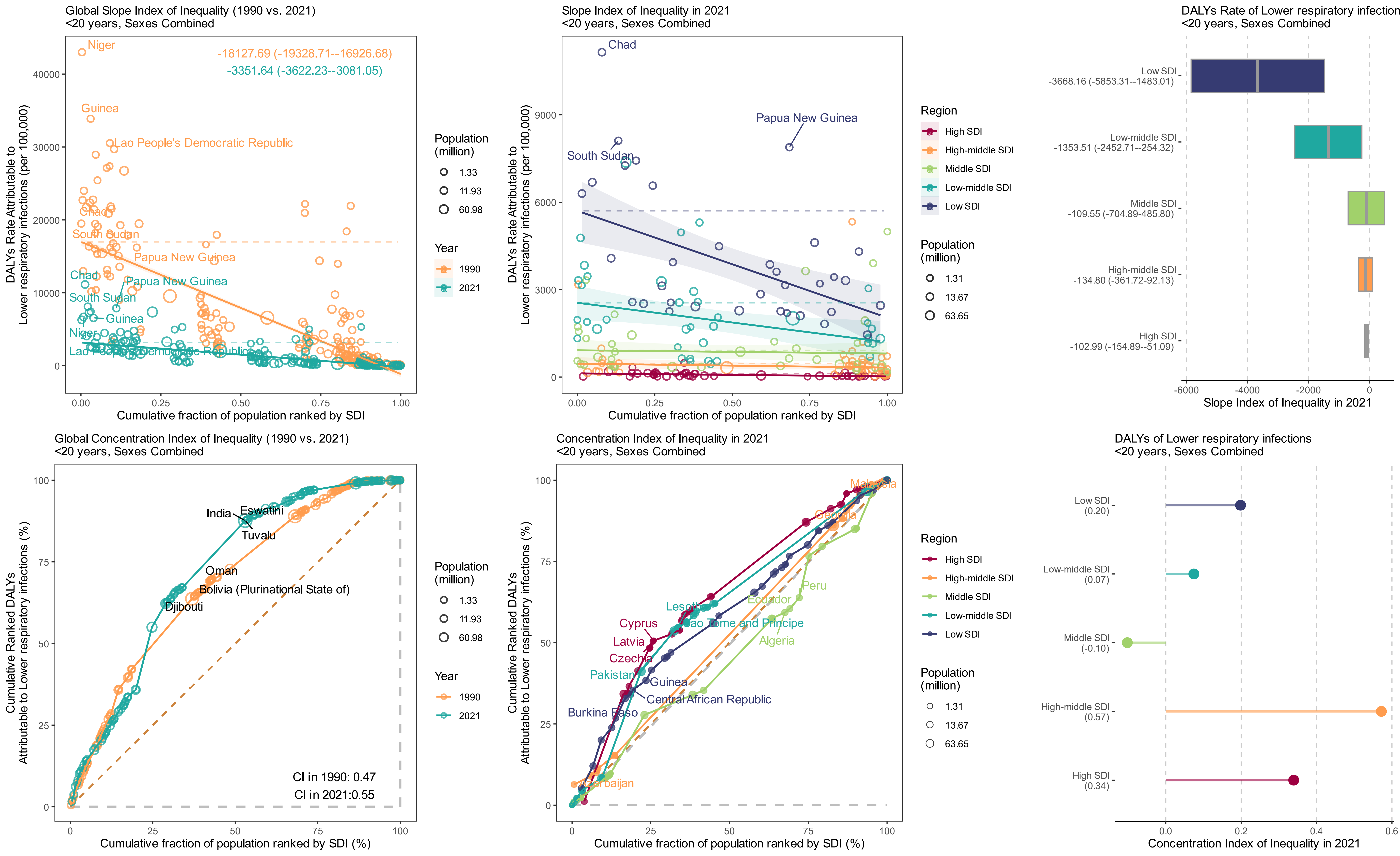
Global Slope Index of Inequality (1990 vs. 2021)
<20 years, Sexes Combined
-18127.69 (-19328.71--16926.68)
-3351.64 (-3622.23--3081.05)
Niger
Guinea
Lao People's Democratic Republic
Chad
South Sudan
Papua New Guinea
DALYs Rate Attributable to Lower respiratory infections (per 100,000)
Cumulative fraction of population ranked by SDI
Population (million)
1.33
11.93
60.98
Year
1990
2021
Slope Index of Inequality in 2021
<20 years, Sexes Combined
Chad
Papua New Guinea
South Sudan
Region
High SDI
High-middle SDI
Middle SDI
Low-middle SDI
Low SDI
Population (million)
1.31
13.67
63.65
DALYs Rate of Lower respiratory infection
<20 years, Sexes Combined
Low SDI
-3668.16 (-5853.31--1483.01)
Low-middle SDI
-1353.51 (-2452.71--254.32)
Middle SDI
-109.55 (-704.89-485.80)
High-middle SDI
-134.80 (-361.72-92.13)
High SDI
-102.99 (-154.89--51.09)
Slope Index of Inequality in 2021
Global Concentration Index of Inequality (1990 vs. 2021)
<20 years, Sexes Combined
India
Eswatini
Tuvalu
Oman
Bolivia (Plurinational State of)
Djibouti
CI in 1990: 0.47
CI in 2021:0.55
Cumulative Ranked DALYs Attributable to Lower respiratory infections (%)
Cumulative fraction of population ranked by SDI (%)
Concentration Index of Inequality in 2021
<20 years, Sexes Combined
Malaysia
Georgia
Peru
Ecuador
Lesotho
Sao Tome and Principe
Algeria
Cyprus
Latvia
Czechia
Pakistan
Guinea
Central African Republic
Burkina Faso
Azerbaijan
DALYs of Lower respiratory infections
<20 years, Sexes Combined
Low SDI (0.20)
Low-middle SDI (0.07)
Middle SDI (-0.10)
High-middle SDI (0.57)
High SDI (0.34)
Concentration Index of Inequality in 2021

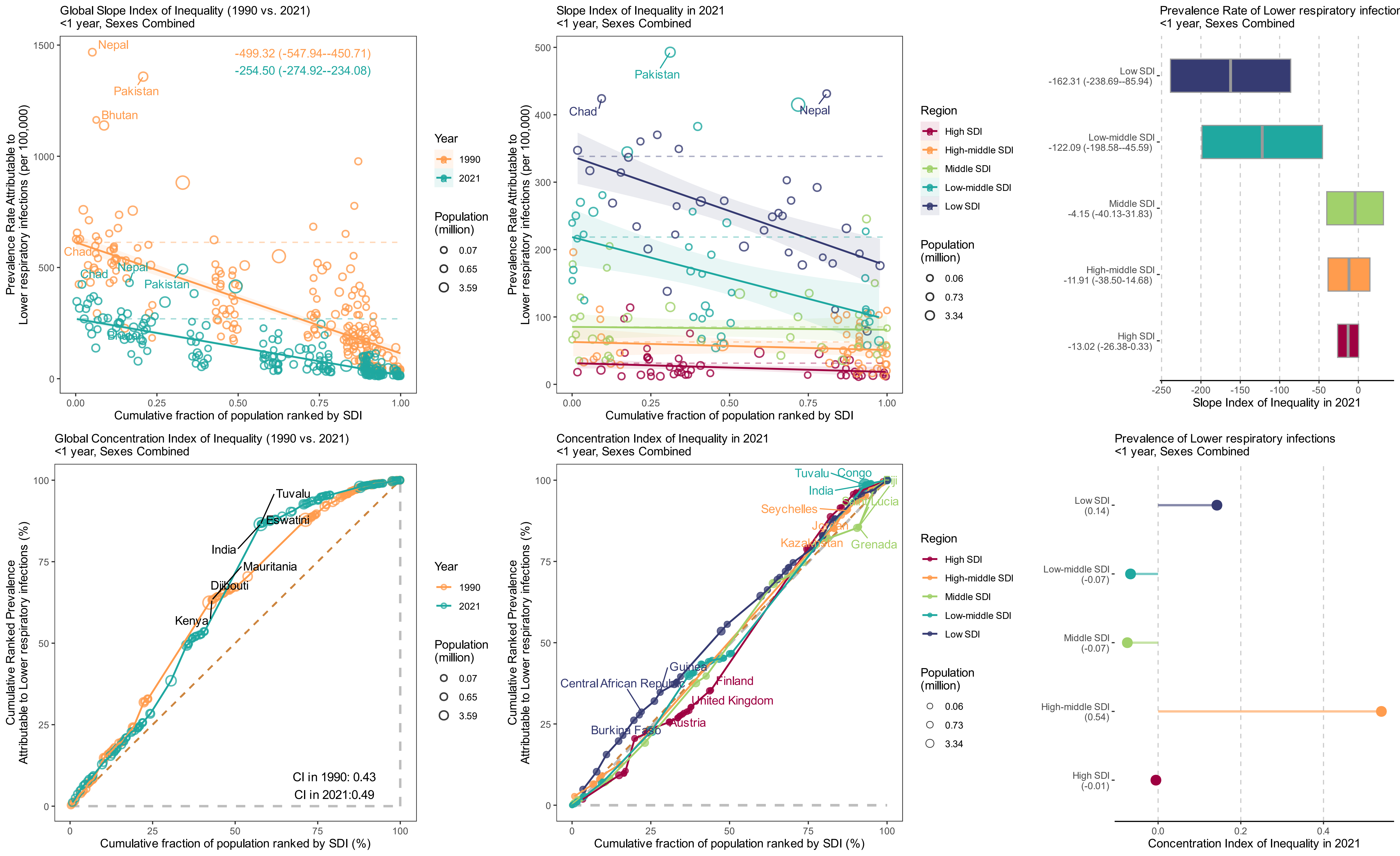
Global Slope Index of Inequality (1990 vs. 2021)
<1 year, Sexes Combined
-499.32 (-547.94--450.71)
-254.50 (-274.92--234.08)
Prevalence Rate Attributable to Lower respiratory infections (per 100,000)
Cumulative fraction of population ranked by SDI
Year
1990
2021
Population (million)
0.07
0.65
3.59
Slope Index of Inequality in 2021
Region
High SDI
High-middle SDI
Middle SDI
Low-middle SDI
Low SDI
Prevalence Rate of Lower respiratory infection
Low SDI -162.31 (-238.69--85.94)
Low-middle SDI -122.09 (-198.58--45.59)
Middle SDI -4.15 (-40.13-31.83)
High-middle SDI -11.91 (-38.50-14.68)
High SDI -13.02 (-26.38-0.33)
Global Concentration Index of Inequality (1990 vs. 2021)
Cumulative Ranked Prevalence Attributable to Lower respiratory infections (%)
Cumulative fraction of population ranked by SDI (%)
CI in 1990: 0.43
CI in 2021:0.49
Concentration Index of Inequality in 2021
Prevalence of Lower respiratory infections
Low SDI (0.14)
Low-middle SDI (-0.07)
Middle SDI (-0.07)
High-middle SDI (0.54)
High SDI (-0.01)

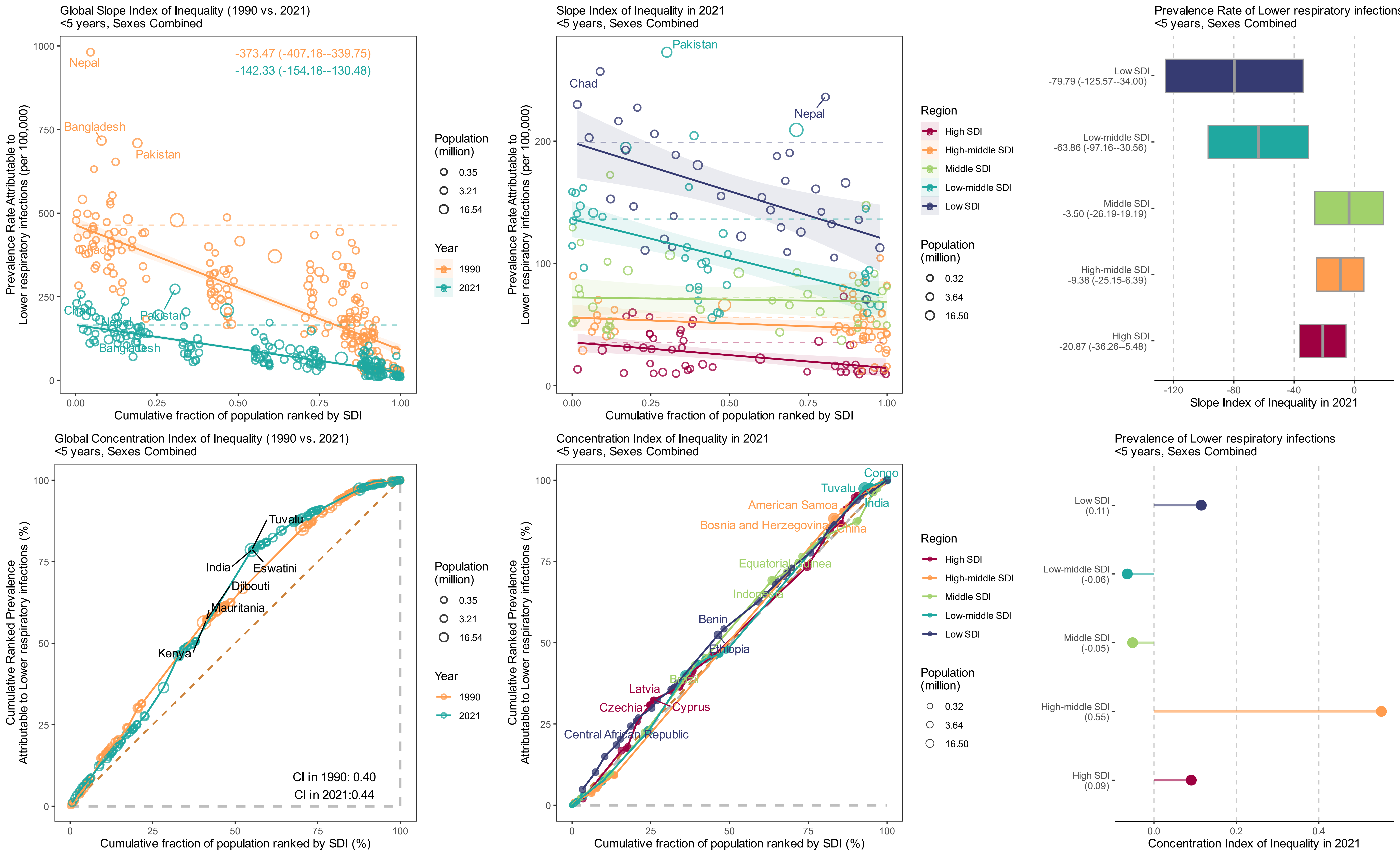
Global Slope Index of Inequality (1990 vs. 2021)
<5 years, Sexes Combined
-373.47 (-407.18--339.75)
-142.33 (-154.18--130.48)
Nepal
Bangladesh
Pakistan
Chad
Prevalence Rate Attributable to Lower respiratory infections (per 100,000)
Cumulative fraction of population ranked by SDI
Population (million)
0.35
3.21
16.54
Year
1990
2021
Slope Index of Inequality in 2021
<5 years, Sexes Combined
Region
High SDI
High-middle SDI
Middle SDI
Low-middle SDI
Low SDI
0.32
3.64
16.50
Prevalence Rate of Lower respiratory infections
Low SDI -79.79 (-125.57--34.00)
Low-middle SDI -63.86 (-97.16--30.56)
Middle SDI -3.50 (-26.19-19.19)
High-middle SDI -9.38 (-25.15-6.39)
High SDI -20.87 (-36.26--5.48)
Slope Index of Inequality in 2021
Global Concentration Index of Inequality (1990 vs. 2021)
Tuvalu
India
Eswatini
Djibouti
Mauritania
Kenya
CI in 1990: 0.40
CI in 2021:0.44
Cumulative Ranked Prevalence Attributable to Lower respiratory infections (%)
Cumulative fraction of population ranked by SDI (%)
Concentration Index of Inequality in 2021
Congo
American Samoa
Bosnia and Herzegovina
China
Equatorial Guinea
Indonesia
Benin
Ethiopia
Brazil
Latvia
Czechia
Cyprus
Central African Republic
Prevalence of Lower respiratory infections
Low SDI (0.11)
Low-middle SDI (-0.06)
Middle SDI (-0.05)
High-middle SDI (0.55)
High SDI (0.09)
Concentration Index of Inequality in 2021

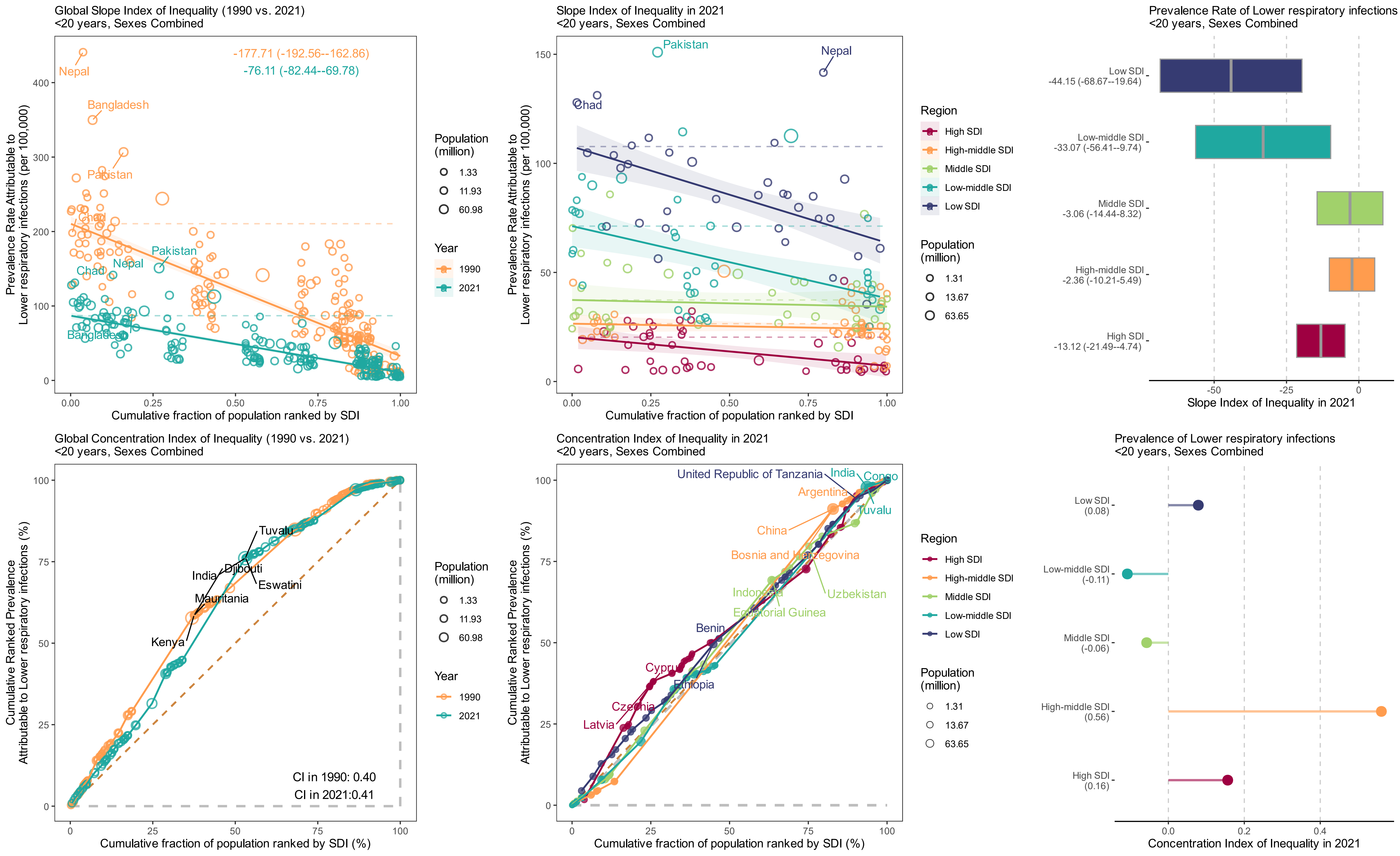
Global Slope Index of Inequality (1990 vs. 2021)
<20 years, Sexes Combined
-177.71 (-192.56--162.86)
-76.11 (-82.44--69.78)
Nepal
Bangladesh
Pakistan
Chad
Pakistan
Nepal
Chad
Bangladesh
Prevalence Rate Attributable to Lower respiratory infections (per 100,000)
Cumulative fraction of population ranked by SDI
Population (million)
1.33
11.93
60.98
Year
1990
2021
Slope Index of Inequality in 2021
<20 years, Sexes Combined
Pakistan
Nepal
Chad
Prevalence Rate Attributable to Lower respiratory infections (per 100,000)
Cumulative fraction of population ranked by SDI
Region
High SDI
High-middle SDI
Middle SDI
Low-middle SDI
Low SDI
Population (million)
1.31
13.67
63.65
Prevalence Rate of Lower respiratory infections
<20 years, Sexes Combined
Low SDI
-44.15 (-68.67--19.64)
Low-middle SDI
-33.07 (-56.41--9.74)
Middle SDI
-3.06 (-14.44-8.32)
High-middle SDI
-2.36 (-10.21-5.49)
High SDI
-13.12 (-21.49--4.74)
Slope Index of Inequality in 2021
Global Concentration Index of Inequality (1990 vs. 2021)
<20 years, Sexes Combined
Tuvalu
India
Djibouti
Eswatini
Mauritania
Kenya
CI in 1990: 0.40
CI in 2021:0.41
Cumulative Ranked Prevalence Attributable to Lower respiratory infections (%)
Cumulative fraction of population ranked by SDI (%)
Population (million)
1.33
11.93
60.98
Year
1990
2021
Concentration Index of Inequality in 2021
<20 years, Sexes Combined
United Republic of Tanzania
India
Congo
Argentina
Tuvalu
China
Bosnia and Herzegovina
Uzbekistan
Indonesia
Equatorial Guinea
Benin
Cyprus
Ethiopia
Czechia
Latvia
Cumulative Ranked Prevalence Attributable to Lower respiratory infections (%)
Cumulative fraction of population ranked by SDI (%)
Region
High SDI
High-middle SDI
Middle SDI
Low-middle SDI
Low SDI
Population (million)
1.31
13.67
63.65
Prevalence of Lower respiratory infections
<20 years, Sexes Combined
Low SDI
(0.08)
Low-middle SDI
(-0.11)
Middle SDI
(-0.06)
High-middle SDI
(0.56)
High SDI
(0.16)
Concentration Index of Inequality in 2021

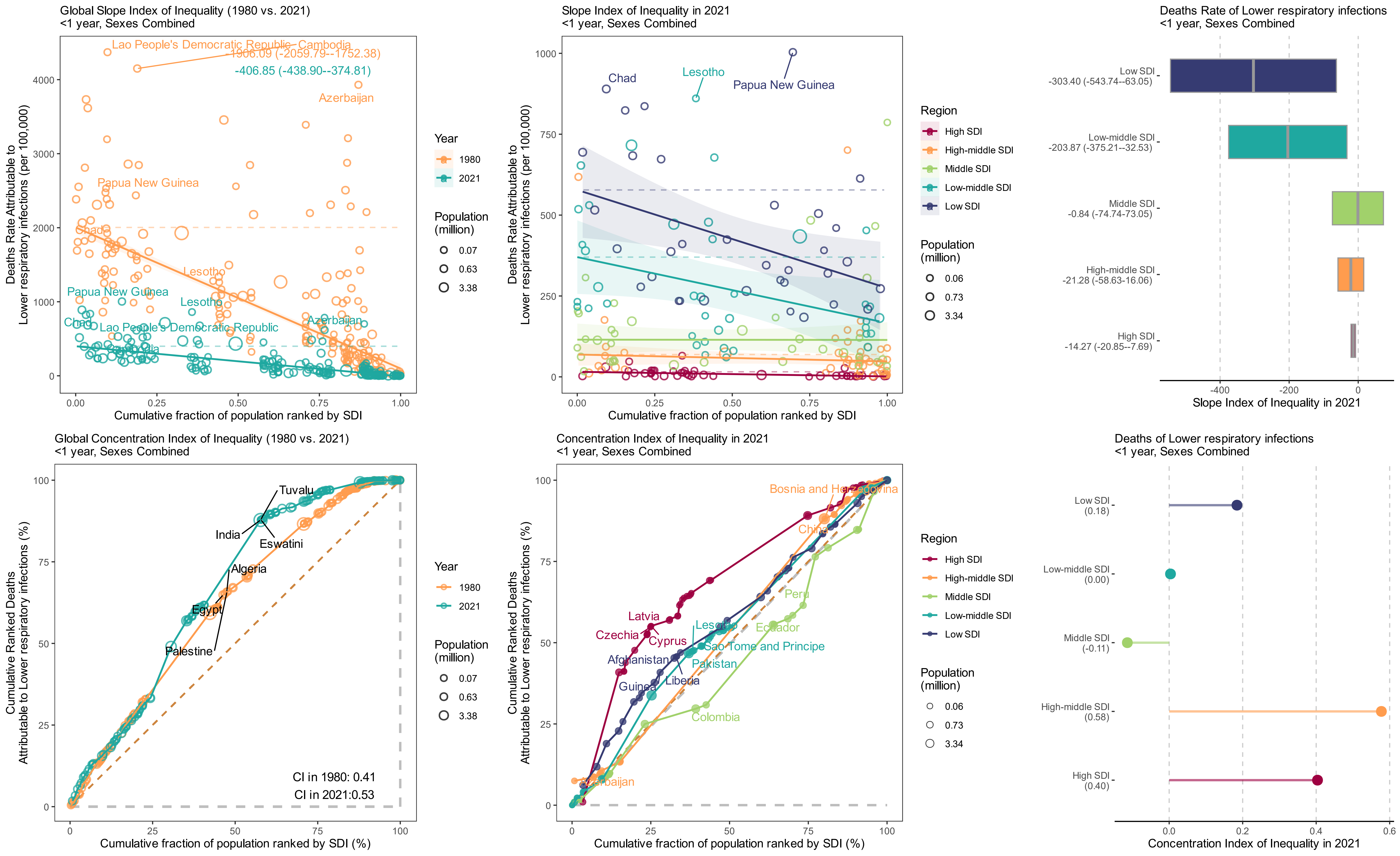

Global Slope Index of Inequality (1980 vs. 2021)
<1 year, Sexes Combined
Deaths Rate Attributable to Lower respiratory infections (per 100,000)
Cumulative fraction of population ranked by SDI
Lao People's Democratic Republic
Cambodia
-1906.09 (-2059.79--1752.38)
-406.85 (-438.90--374.81)
Azerbaijan
Papua New Guinea
Chad
Lesotho
Year
1980
2021
Population (million)
0.07
0.63
3.38
Slope Index of Inequality in 2021
<1 year, Sexes Combined
Chad
Lesotho
Papua New Guinea
Region
High SDI
High-middle SDI
Middle SDI
Low-middle SDI
Low SDI
Population (million)
0.06
0.73
3.34
Deaths Rate of Lower respiratory infections
<1 year, Sexes Combined
Low SDI
-303.40 (-543.74--63.05)
Low-middle SDI
-203.87 (-375.21--32.53)
Middle SDI
-0.84 (-74.74-73.05)
High-middle SDI
-21.28 (-58.63-16.06)
High SDI
-14.27 (-20.85--7.69)
Slope Index of Inequality in 2021
Global Concentration Index of Inequality (1980 vs. 2021)
<1 year, Sexes Combined
Cumulative Ranked Deaths Attributable to Lower respiratory infections (%)
Cumulative fraction of population ranked by SDI (%)
Tuvalu
India
Eswatini
Algeria
Egypt
Palestine
CI in 1980: 0.41
CI in 2021:0.53
Concentration Index of Inequality in 2021
<1 year, Sexes Combined
Bosnia and Herzegovina
China
Peru
Latvia
Czechia
Cyprus
Lesotho
Ecuador
Sao Tome and Principe
Afghanistan
Pakistan
Liberia
Guinea
Colombia
Azerbaijan
Deaths of Lower respiratory infections
<1 year, Sexes Combined
Low SDI
(0.18)
Low-middle SDI
(0.00)
Middle SDI
(-0.11)
High-middle SDI
(0.58)
High SDI
(0.40)
Concentration Index of Inequality in 2021

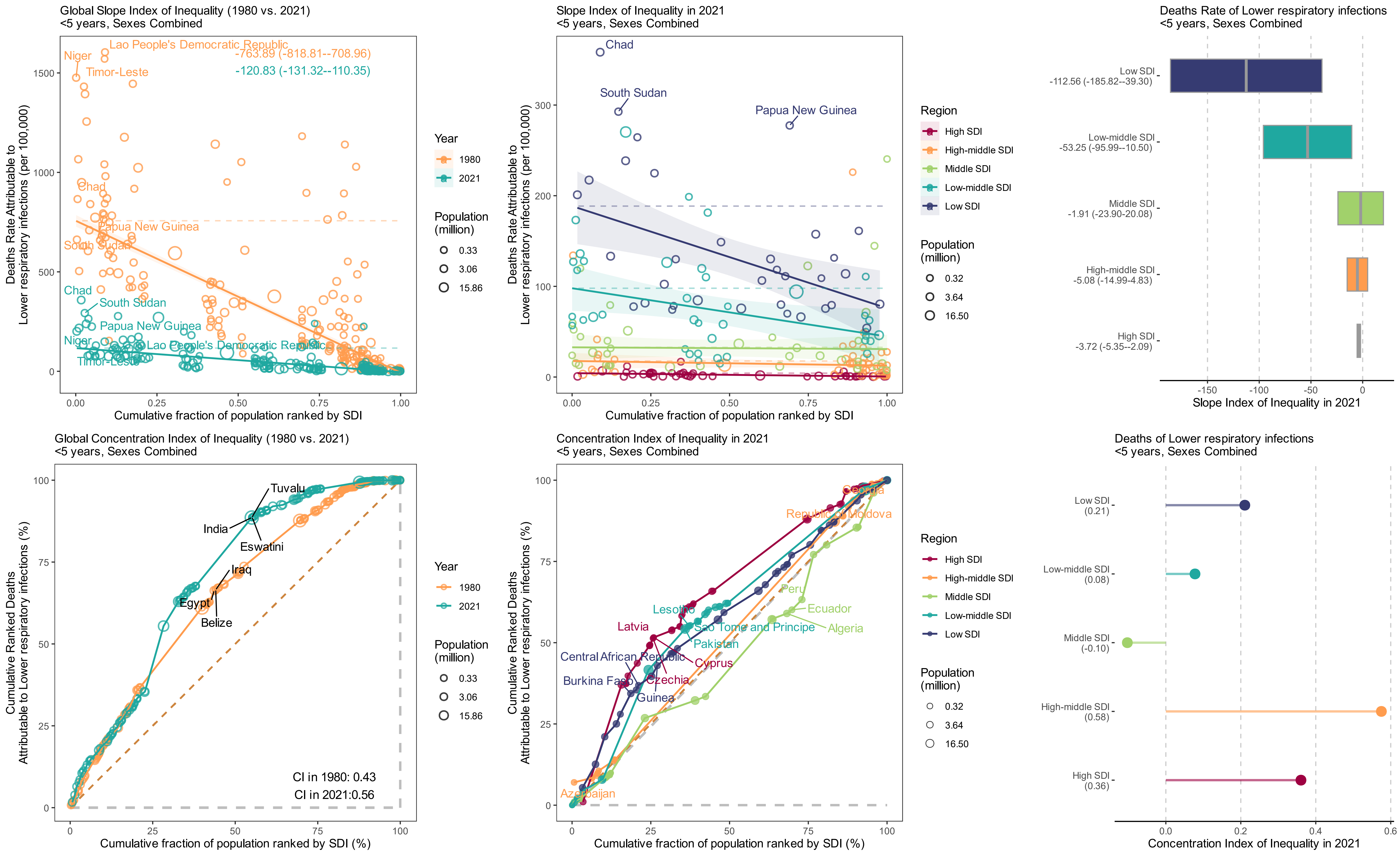

Global Slope Index of Inequality (1980 vs. 2021)
<5 years, Sexes Combined
-763.89 (-818.81--708.96)
-120.83 (-131.32--110.35)
Deaths Rate Attributable to Lower respiratory infections (per 100,000)
Cumulative fraction of population ranked by SDI
Niger
Lao People's Democratic Republic
Timor-Leste
Chad
Papua New Guinea
South Sudan
Year
1980
2021
Population (million)
0.33
3.06
15.86
Slope Index of Inequality in 2021
<5 years, Sexes Combined
Region
High SDI
High-middle SDI
Middle SDI
Low-middle SDI
Low SDI
Population (million)
0.32
3.64
16.50
Deaths Rate of Lower respiratory infections
<5 years, Sexes Combined
Low SDI -112.56 (-185.82--39.30)
Low-middle SDI -53.25 (-95.99--10.50)
Middle SDI -1.91 (-23.90-20.08)
High-middle SDI -5.08 (-14.99-4.83)
High SDI -3.72 (-5.35--2.09)
Slope Index of Inequality in 2021
Global Concentration Index of Inequality (1980 vs. 2021)
<5 years, Sexes Combined
Cumulative Ranked Deaths Attributable to Lower respiratory infections (%)
Cumulative fraction of population ranked by SDI (%)
Tuvalu
India
Eswatini
Iraq
Egypt
Belize
CI in 1980: 0.43
CI in 2021:0.56
Concentration Index of Inequality in 2021
<5 years, Sexes Combined
Georgia
Republic of Moldova
Peru
Lesotho
Ecuador
Latvia
Sao Tome and Principe
Algeria
Pakistan
Central African Republic
Cyprus
Burkina Faso
Czechia
Guinea
Azerbaijan
Deaths of Lower respiratory infections
<5 years, Sexes Combined
Low SDI (0.21)
Low-middle SDI (0.08)
Middle SDI (-0.10)
High-middle SDI (0.58)
High SDI (0.36)
Concentration Index of Inequality in 2021

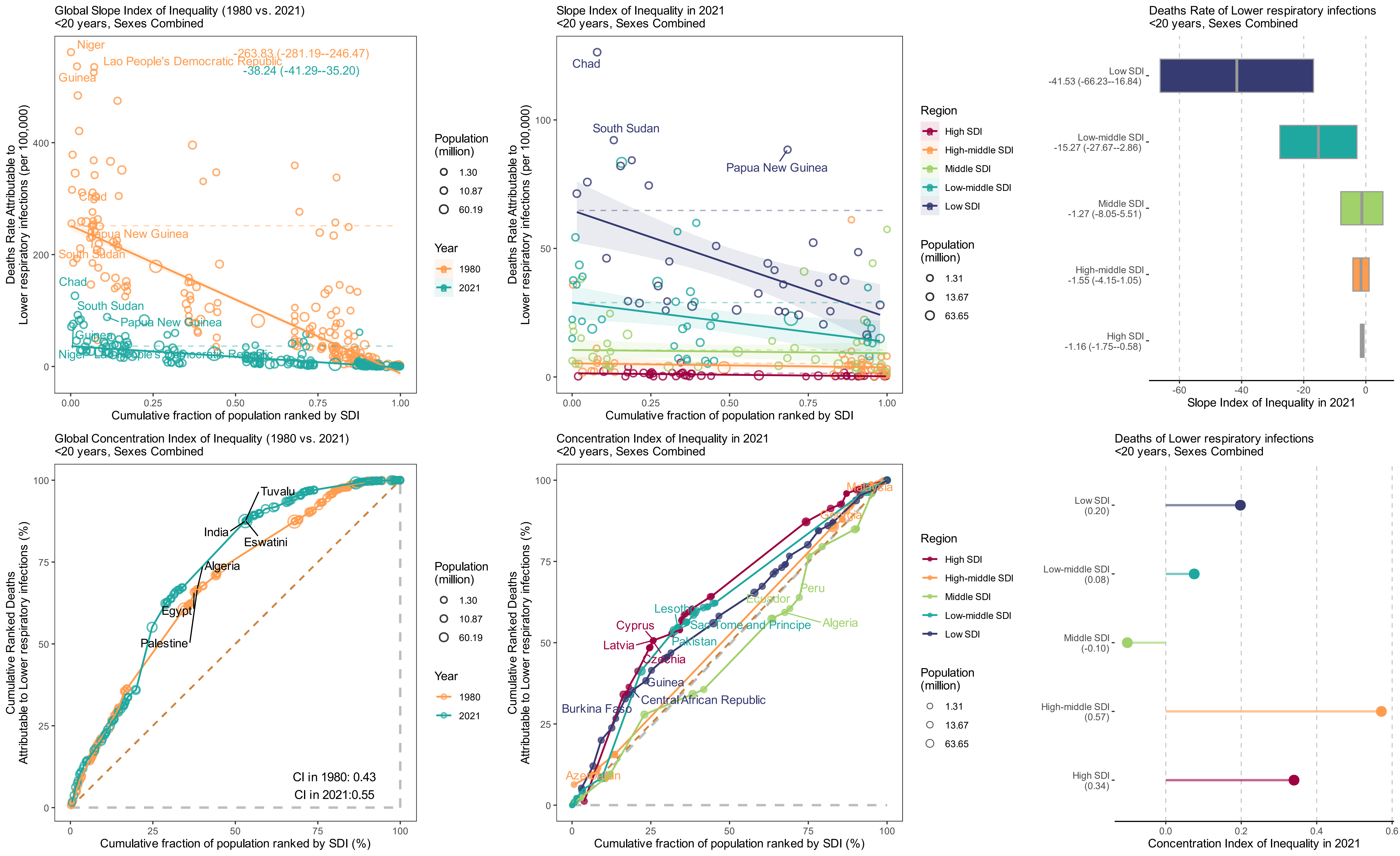

Global Slope Index of Inequality (1980 vs. 2021)
<20 years, Sexes Combined
Niger
Lao People's Democratic Republic
Guinea
Chad
Papua New Guinea
South Sudan
-263.83 (-281.19--246.47)
-38.24 (-41.29--35.20)
Deaths Rate Attributable to Lower respiratory infections (per 100,000)
Cumulative fraction of population ranked by SDI
Population (million)
1.30
10.87
60.19
Year
1980
2021
Slope Index of Inequality in 2021
<20 years, Sexes Combined
Chad
South Sudan
Papua New Guinea
Region
High SDI
High-middle SDI
Middle SDI
Low-middle SDI
Low SDI
Population (million)
1.31
13.67
63.65
Deaths Rate of Lower respiratory infections
<20 years, Sexes Combined
Low SDI
-41.53 (-66.23--16.84)
Low-middle SDI
-15.27 (-27.67--2.86)
Middle SDI
-1.27 (-8.05-5.51)
High-middle SDI
-1.55 (-4.15-1.05)
High SDI
-1.16 (-1.75--0.58)
Slope Index of Inequality in 2021
Global Concentration Index of Inequality (1980 vs. 2021)
<20 years, Sexes Combined
Tuvalu
India
Eswatini
Algeria
Egypt
Palestine
CI in 1980: 0.43
CI in 2021:0.55
Cumulative Ranked Deaths Attributable to Lower respiratory infections (%)
Cumulative fraction of population ranked by SDI (%)
Concentration Index of Inequality in 2021
<20 years, Sexes Combined
Malaysia
Georgia
Peru
Ecuador
Algeria
Lesotho
Sao Tome and Principe
Cyprus
Pakistan
Latvia
Czechia
Guinea
Central African Republic
Burkina Faso
Azerbaijan
Deaths of Lower respiratory infections
<20 years, Sexes Combined
Low SDI (0.20)
Low-middle SDI (0.08)
Middle SDI (-0.10)
High-middle SDI (0.57)
High SDI (0.34)
Concentration Index of Inequality in 2021

Frontier analysis

Metric	Yeas	Regional	Lower respiratory infections|<1 year|Female		
Deaths	Year 2021		Countries behind frontier n (%)	170 (83.3%)	Tokelau [809.5 (431.6-1114.2)]; Niue [796.7 (601.2-1002.4)]; Papua New Guinea [713.4 (461.5-974.4)]
Deaths	Year 2021	SDI Region (% in Top 100)	High (%)	1.00%	Kuwait [65.5 (52.3-81.3)]
Deaths	Year 2021	SDI Region (% in Top 100)	High-middle (%)	22.00%	Niue [796.7 (601.2-1002.4)]; Azerbaijan [634.3 (510.4-787.2)]; Palau [186.5 (136.6-247.3)]
Deaths	Year 2021	SDI Region (% in Top 100)	Middle (%)	23.00%	Tokelau [809.5 (431.6-1114.2)]; Uzbekistan [443.8 (356.6-543.5)]; Turkmenistan [412.7 (324.5-507.2)]
Deaths	Year 2021	SDI Region (% in Top 100)	Low-middle (%)	32.00%	Tajikistan [562.9 (403.1-768.3)]; Lesotho [558.2 (299.2-920.7)]; Zimbabwe [519.9 (335.4-712.3)]
Deaths	Year 2021	SDI Region (% in Top 100)	Low (%)	22.00%	Papua New Guinea [713.4 (461.5-974.4)]; Central African Republic [516.2 (234.9-835.4)]; South Sudan [461.5 (132.2-1191.5)]
Deaths	Year 2021	GBD Region (% in Top 100)	Top 1	Oceania (17.0%)	Tokelau [809.5 (431.6-1114.2)]; Niue [796.7 (601.2-1002.4)]; Papua New Guinea [713.4 (461.5-974.4)]
Deaths	Year 2021	GBD Region (% in Top 100)	Top 2	Western Sub-Saharan Africa (12.0%)	Nigeria [511.2 (363.4-655.0)]; Guinea [295.5 (112.1-482.7)]; Burkina Faso [287.1 (92.2-514.3)]
Deaths	Year 2021	GBD Region (% in Top 100)	Top 3	Caribbean (9.0%)	Haiti [388.2 (161.6-644.8)]; Dominica [115.6 (75.8-174.3)]; Guyana [95.6 (66.4-132.6)]
Deaths	1980-2021		Countries behind frontier n (%)	3 (1.5%)	Tokelau [658.4 (394.1-847.6)]; Niue [374.1 (320.3-432.9)]; Dominica [37.5 (39.5-47.1)]
Deaths	1980-2021	SDI Region (% in	High (%)	0.00%	-

		All FBF increased)			
Deaths	1980-2021	SDI Region (% in All FBF increased)	High-middle (%)	66.70%	Niue [374.1 (320.3-432.9)]; Dominica [37.5 (39.5-47.1)]
Deaths	1980-2021	SDI Region (% in All FBF increased)	Middle (%)	33.30%	Tokelau [658.4 (394.1-847.6)]
Deaths	1980-2021	SDI Region (% in All FBF increased)	Low-middle (%)	0.00%	-
Deaths	1980-2021	SDI Region (% in All FBF increased)	Low (%)	0.00%	-
Deaths	1980-2021	GBD Region (% in All FBF increased)	Top 1	Oceania (66.7%)	Tokelau [658.4 (394.1-847.6)]; Niue [374.1 (320.3-432.9)]
Deaths	1980-2021	GBD Region (% in All FBF increased)	Top 2	Caribbean (33.3%)	Dominica [37.5 (39.5-47.1)]
Deaths	1980-2021	GBD Region (% in All FBF increased)	Top 3	NA (NA%)	-
DALYs	Year 2021		Countries behind frontier n (%)	169 (82.8%)	Tokelau [72560.0 (38719.7-99873.7)]; Niue [71398.4 (53861.6-89839.6)]; Papua New Guinea [63994.0 (41394.4-87414.5)]
DALYs	Year 2021	SDI Region (% in Top 100)	High (%)	2.00%	Kuwait [5885.7 (4696.9-7303.7)]; Slovakia [3601.8 (2685.2-4521.6)]
DALYs	Year 2021	SDI Region (% in Top 100)	High-middle (%)	23.00%	Niue [71398.4 (53861.6-89839.6)]; Azerbaijan [56943.7 (45835.6-70644.8)]; Palau [16723.3 (12246.1-22169.3)]
DALYs	Year 2021	SDI Region (% in	Middle (%)	22.00%	Tokelau [72560.0 (38719.7-99873.7)]; Uzbekistan

		Top 100)			[39824.8 (32000.9-48774.9)]; Turkmenistan [37009.8 (29102.0-45474.4)]
DALYs	Year 2021	SDI Region (% in Top 100)	Low-middle (%)	31.00%	Tajikistan [50504.5 (36172.3-68948.3)]; Lesotho [49787.6 (26573.3-82222.6)]; Nigeria [45856.4 (32594.5-58753.9)]
DALYs	Year 2021	SDI Region (% in Top 100)	Low (%)	22.00%	Papua New Guinea [63994.0 (41394.4-87414.5)]; Central African Republic [46653.2 (21410.8-75325.2)]; South Sudan [41424.1 (11878.4-106933.0)]
DALYs	Year 2021	GBD Region (% in Top 100)	Top 1	Oceania (17.0%)	Tokelau [72560.0 (38719.7-99873.7)]; Niue [71398.4 (53861.6-89839.6)]; Papua New Guinea [63994.0 (41394.4-87414.5)]
DALYs	Year 2021	GBD Region (% in Top 100)	Top 2	Western Sub-Saharan Africa (12.0%)	Nigeria [45856.4 (32594.5-58753.9)]; Guinea [26475.0 (10020.6-43286.4)]; Burkina Faso [26014.9 (8527.5-46422.3)]
DALYs	Year 2021	GBD Region (% in Top 100)	Top 3	Central Asia (9.0%)	Azerbaijan [56943.7 (45835.6-70644.8)]; Tajikistan [50504.5 (36172.3-68948.3)]; Uzbekistan [39824.8 (32000.9-48774.9)]
DALYs	1990-2021		Countries behind frontier n (%)	7 (3.4%)	Tokelau [60269.5 (33405.0-81378.6)]; Niue [37464.7 (29378.7-45711.6)]; Dominica [5955.8 (4749.6-8600.3)]
DALYs	1990-2021	SDI Region (% in All FBF increased)	High (%)	0.00%	-
DALYs	1990-2021	SDI Region (% in All FBF increased)	High-middle (%)	42.90%	Niue [37464.7 (29378.7-45711.6)]; Dominica [5955.8 (4749.6-8600.3)]; Antigua and Barbuda [1094.2 (708.5-1469.6)]
DALYs	1990-2021	SDI Region (% in All FBF increased)	Middle (%)	57.10%	Tokelau [60269.5 (33405.0-81378.6)]; Saint Lucia [3124.9 (307.0-2839.8)]; Saint Vincent and the Grenadines [2496.0 (271.7-849.8)]
DALYs	1990-2021	SDI Region (% in All FBF	Low-middle (%)	0.00%	-

		increased)			
DALYs	1990-2021	SDI Region (% in All FBF increased)	Low (%)	0.00%	-
DALYs	1990-2021	GBD Region (% in All FBF increased)	Top 1	Caribbean (71.4%)	Dominica [5955.8 (4749.6-8600.3)]; Saint Lucia [3124.9 (307.0-2839.8)]; Saint Vincent and the Grenadines [2496.0 (271.7-849.8)]
DALYs	1990-2021	GBD Region (% in All FBF increased)	Top 2	Oceania (28.6%)	Tokelau [60269.5 (33405.0-81378.6)]; Niue [37464.7 (29378.7-45711.6)]
DALYs	1990-2021	GBD Region (% in All FBF increased)	Top 3	NA (NA%)	-
YLDs	Year 2021		Countries behind frontier n (%)	113 (55.4%)	Pakistan [24.1 (13.7-38.2)]; India [20.8 (12.6-32.2)]; Kenya [19.1 (11.6-29.5)]
YLDs	Year 2021	SDI Region (% in Top 100)	High (%)	7.00%	Poland [5.3 (3.2-8.1)]; Brunei Darussalam [4.8 (2.4-8.0)]; Singapore [3.8 (1.8-6.6)]
YLDs	Year 2021	SDI Region (% in Top 100)	High-middle (%)	20.00%	Azerbaijan [10.3 (5.9-17.1)]; Guam [5.8 (3.1-9.6)]; Niue [5.1 (2.2-9.0)]
YLDs	Year 2021	SDI Region (% in Top 100)	Middle (%)	17.00%	South Africa [13.6 (8.4-21.6)]; Botswana [10.2 (5.5-17.3)]; Namibia [8.3 (3.7-14.5)]
YLDs	Year 2021	SDI Region (% in Top 100)	Low-middle (%)	31.00%	Pakistan [24.1 (13.7-38.2)]; India [20.8 (12.6-32.2)]; Kenya [19.1 (11.6-29.5)]
YLDs	Year 2021	SDI Region (% in Top 100)	Low (%)	25.00%	Nepal [16.3 (7.4-29.4)]; Guinea [10.6 (2.6-23.0)]; Central African Republic [9.3 (1.3-19.4)]
YLDs	Year 2021	GBD Region (% in Top 100)	Top 1	Oceania (18.0%)	Papua New Guinea [6.9 (1.2-14.0)]; Guam [5.8 (3.1-9.6)]; Nauru [5.3 (2.2-9.4)]
YLDs	Year 2021	GBD Region (% in Top 100)	Top 2	Western Sub-Saharan Africa (17.0%)	Nigeria [15.1 (8.5-24.8)]; Mauritania [11.0 (4.9-20.0)]; Guinea [10.6 (2.6-23.0)]

YLDs	Year 2021	GBD Region (% in Top 100)	Top 3	Eastern Sub-Saharan Africa (11.0%)	Kenya [19.1 (11.6-29.5)]; Comoros [7.7 (2.6-14.0)]; Djibouti [7.3 (2.5-14.1)]
YLDs	1990-2021		Countries behind frontier n (%)	2 (1.0%)	Somalia [0.1 (3.8-5.3)]; France [0.0 (0.5-0.6)]; NA [NA]
YLDs	1990-2021	SDI Region (% in All FBF increased)	High (%)	50.00%	France [0.0 (0.5-0.6)]
YLDs	1990-2021	SDI Region (% in All FBF increased)	High-middle (%)	0.00%	-
YLDs	1990-2021	SDI Region (% in All FBF increased)	Middle (%)	0.00%	-
YLDs	1990-2021	SDI Region (% in All FBF increased)	Low-middle (%)	0.00%	-
YLDs	1990-2021	SDI Region (% in All FBF increased)	Low (%)	50.00%	Somalia [0.1 (3.8-5.3)]
YLDs	1990-2021	GBD Region (% in All FBF increased)	Top 1	Eastern Sub-Saharan Africa (50.0%)	Somalia [0.1 (3.8-5.3)]
YLDs	1990-2021	GBD Region (% in All FBF increased)	Top 2	Western Europe (50.0%)	France [0.0 (0.5-0.6)]
YLDs	1990-2021	GBD Region (% in All FBF increased)	Top 3	NA (NA%)	-

YLLs	Year 2021		Countries behind frontier n (%)	170 (83.3%)	Tokelau [72552.9 (38714.0-99864.8)]; Niue [71393.5 (53857.8-89832.9)]; Papua New Guinea [63907.5 (41314.0-87322.3)]
YLLs	Year 2021	SDI Region (% in Top 100)	High (%)	1.00%	Kuwait [5883.4 (4694.5-7301.8)]
YLLs	Year 2021	SDI Region (% in Top 100)	High-middle (%)	22.00%	Niue [71393.5 (53857.8-89832.9)]; Azerbaijan [56927.3 (45818.8-70629.9)]; Palau [16717.0 (12240.4-22164.3)]
YLLs	Year 2021	SDI Region (% in Top 100)	Middle (%)	23.00%	Tokelau [72552.9 (38714.0-99864.8)]; Uzbekistan [39812.8 (31989.9-48759.8)]; Turkmenistan [37002.2 (29094.2-45466.8)]
YLLs	Year 2021	SDI Region (% in Top 100)	Low-middle (%)	32.00%	Tajikistan [50509.5 (36177.2-68953.2)]; Lesotho [50137.0 (26905.5-82590.8)]; Zimbabwe [46597.8 (30057.4-63861.2)]
YLLs	Year 2021	SDI Region (% in Top 100)	Low (%)	22.00%	Papua New Guinea [63907.5 (41314.0-87322.3)]; Central African Republic [46547.5 (21325.9-75198.2)]; South Sudan [41432.9 (11881.1-106957.9)]
YLLs	Year 2021	GBD Region (% in Top 100)	Top 1	Oceania (17.0%)	Tokelau [72552.9 (38714.0-99864.8)]; Niue [71393.5 (53857.8-89832.9)]; Papua New Guinea [63907.5 (41314.0-87322.3)]
YLLs	Year 2021	GBD Region (% in Top 100)	Top 2	Western Sub-Saharan Africa (12.0%)	Nigeria [45807.0 (32549.1-58702.2)]; Guinea [26462.8 (10009.2-43273.2)]; Burkina Faso [25973.2 (8485.7-46377.3)]
YLLs	Year 2021	GBD Region (% in Top 100)	Top 3	Caribbean (9.0%)	Haiti [34853.7 (14522.1-57865.5)]; Dominica [10354.0 (6788.9-15626.2)]; Guyana [8570.6 (5958.9-11893.5)]
YLLs	1980-2021		Countries behind frontier n (%)	3 (1.5%)	Tokelau [59023.9 (35367.3-75962.2)]; Niue [33565.5 (28727.5-38853.5)]; Dominica [3388.7 (3571.3-4250.7)]
YLLs	1980-2021	SDI Region (% in All FBF increased)	High (%)	0.00%	-
YLLs	1980-2021	SDI Region (% in	High-middle (%)	66.70%	Niue [33565.5 (28727.5-38853.5)]; Dominica [3388.7

		All FBF increased)			(3571.3-4250.7)]
YLLs	1980-2021	SDI Region (% in All FBF increased)	Middle (%)	33.30%	Tokelau [59023.9 (35367.3-75962.2)]
YLLs	1980-2021	SDI Region (% in All FBF increased)	Low-middle (%)	0.00%	-
YLLs	1980-2021	SDI Region (% in All FBF increased)	Low (%)	0.00%	-
YLLs	1980-2021	GBD Region (% in All FBF increased)	Top 1	Oceania (66.7%)	Tokelau [59023.9 (35367.3-75962.2)]; Niue [33565.5 (28727.5-38853.5)]
YLLs	1980-2021	GBD Region (% in All FBF increased)	Top 2	Caribbean (33.3%)	Dominica [3388.7 (3571.3-4250.7)]
YLLs	1980-2021	GBD Region (% in All FBF increased)	Top 3	NA (NA%)	-
Incidence	Year 2021		Countries behind frontier n (%)	166 (81.4%)	Pakistan [17796.1 (15131.3-20925.3)]; India [15325.5 (13013.6-17962.8)]; Kenya [14067.8 (12122.6-16177.5)]
Incidence	Year 2021	SDI Region (% in Top 100)	High (%)	7.00%	Poland [3704.1 (3251.1-4240.2)]; Brunei Darussalam [3417.8 (2875.7-3997.9)]; Singapore [2645.6 (2232.2-3109.6)]
Incidence	Year 2021	SDI Region (% in Top 100)	High-middle (%)	22.00%	Azerbaijan [8381.6 (7441.9-9480.4)]; Guam [4476.7 (3859.3-5293.5)]; Northern Mariana Islands [4089.1 (3407.2-5020.9)]
Incidence	Year 2021	SDI Region (% in Top 100)	Middle (%)	17.00%	South Africa [9886.2 (8615.0-11380.2)]; Botswana [8028.0 (6724.1-9554.4)]; Namibia [6380.9 (5233.2-7668.0)]

Incidence	Year 2021	SDI Region (% in Top 100)	Low-middle (%)	31.00%	Pakistan [17796.1 (15131.3-20925.3)]; India [15325.5 (13013.6-17962.8)]; Kenya [14067.8 (12122.6-16177.5)]
Incidence	Year 2021	SDI Region (% in Top 100)	Low (%)	23.00%	Nepal [10845.2 (8211.4-13851.8)]; Guinea [8220.1 (6041.6-10693.2)]; Central African Republic [7684.3 (5537.0-9976.4)]
Incidence	Year 2021	GBD Region (% in Top 100)	Top 1	Oceania (18.0%)	Papua New Guinea [5708.0 (4360.8-7131.5)]; Guam [4476.7 (3859.3-5293.5)]; Nauru [4429.6 (3600.8-5384.1)]
Incidence	Year 2021	GBD Region (% in Top 100)	Top 2	Western Sub-Saharan Africa (16.0%)	Nigeria [12137.2 (10627.8-13867.0)]; Guinea [8220.1 (6041.6-10693.2)]; Ghana [7903.1 (6397.7-9499.3)]
Incidence	Year 2021	GBD Region (% in Top 100)	Top 3	Eastern Sub-Saharan Africa (11.0%)	Kenya [14067.8 (12122.6-16177.5)]; United Republic of Tanzania [5863.7 (4463.1-7638.0)]; Comoros [5683.5 (4253.9-7336.3)]
Incidence	1990-2021		Countries behind frontier n (%)	3 (1.5%)	Somalia [95.4 (1738.4-1979.4)]; Malta [39.7 (84.1-120.0)]; France [8.9 (81.9-89.2)]
Incidence	1990-2021	SDI Region (% in All FBF increased)	High (%)	33.30%	France [8.9 (81.9-89.2)]
Incidence	1990-2021	SDI Region (% in All FBF increased)	High-middle (%)	33.30%	Malta [39.7 (84.1-120.0)]
Incidence	1990-2021	SDI Region (% in All FBF increased)	Middle (%)	0.00%	-
Incidence	1990-2021	SDI Region (% in All FBF increased)	Low-middle (%)	0.00%	-
Incidence	1990-2021	SDI Region (% in	Low (%)	33.30%	Somalia [95.4 (1738.4-1979.4)]

		All FBF increased)			
Incidence	1990-2021	GBD Region (% in All FBF increased)	Top 1	Western Europe (66.7%)	Malta [39.7 (84.1-120.0)]; France [8.9 (81.9-89.2)]
Incidence	1990-2021	GBD Region (% in All FBF increased)	Top 2	Eastern Sub-Saharan Africa (33.3%)	Somalia [95.4 (1738.4-1979.4)]
Incidence	1990-2021	GBD Region (% in All FBF increased)	Top 3	NA (NA%)	-
			Lower respiratory infections|<5 years|Female		
Deaths	Year 2021		Countries behind frontier n (%)	167 (81.9%)	Tokelau [252.7 (181.6-317.9)]; Niue [231.6 (181.5-280.8)]; Nigeria [214.9 (138.4-290.7)]
Deaths	Year 2021	SDI Region (% in Top 100)	High (%)	1.00%	Kuwait [17.2 (13.6-21.2)]
Deaths	Year 2021	SDI Region (% in Top 100)	High-middle (%)	22.00%	Niue [231.6 (181.5-280.8)]; Azerbaijan [132.8 (105.6-167.6)]; Palau [51.1 (38.6-67.5)]
Deaths	Year 2021	SDI Region (% in Top 100)	Middle (%)	23.00%	Tokelau [252.7 (181.6-317.9)]; Turkmenistan [126.8 (101.3-155.8)]; Nauru [115.6 (80.5-160.1)]
Deaths	Year 2021	SDI Region (% in Top 100)	Low-middle (%)	32.00%	Nigeria [214.9 (138.4-290.7)]; Tajikistan [149.5 (108.0-200.8)]; Zimbabwe [136.9 (91.2-187.8)]
Deaths	Year 2021	SDI Region (% in Top 100)	Low (%)	22.00%	Papua New Guinea [190.6 (122.5-264.3)]; South Sudan [187.1 (75.3-441.8)]; Central African Republic [181.2 (90.7-295.0)]
Deaths	Year 2021	GBD Region (% in Top 100)	Top 1	Oceania (17.0%)	Tokelau [252.7 (181.6-317.9)]; Niue [231.6 (181.5-280.8)]; Papua New Guinea [190.6 (122.5-264.3)]
Deaths	Year 2021	GBD Region (% in	Top 2	Eastern	South Sudan [187.1 (75.3-441.8)]; Comoros [72.9

		Top 100)		Sub-Saharan Africa (11.0%)	(35.0-120.8)]; United Republic of Tanzania [52.9 (22.1-87.8)]
Deaths	Year 2021	GBD Region (% in Top 100)	Top 3	Western Sub-Saharan Africa (11.0%)	Nigeria [214.9 (138.4-290.7)]; Chad [131.2 (48.3-228.2)]; Burkina Faso [124.9 (56.4-195.3)]
Deaths	1980-2021		Countries behind frontier n (%)	3 (1.5%)	Niue [128.5 (116.7-137.1)]; Tokelau [22.4 (1.9-36.0)]; Dominica [13.2 (13.9-14.6)]
Deaths	1980-2021	SDI Region (% in All FBF increased)	High (%)	0.00%	-
Deaths	1980-2021	SDI Region (% in All FBF increased)	High-middle (%)	66.70%	Niue [128.5 (116.7-137.1)]; Dominica [13.2 (13.9-14.6)]
Deaths	1980-2021	SDI Region (% in All FBF increased)	Middle (%)	33.30%	Tokelau [22.4 (1.9-36.0)]
Deaths	1980-2021	SDI Region (% in All FBF increased)	Low-middle (%)	0.00%	-
Deaths	1980-2021	SDI Region (% in All FBF increased)	Low (%)	0.00%	-
Deaths	1980-2021	GBD Region (% in All FBF increased)	Top 1	Oceania (66.7%)	Niue [128.5 (116.7-137.1)]; Tokelau [22.4 (1.9-36.0)]
Deaths	1980-2021	GBD Region (% in All FBF increased)	Top 2	Caribbean (33.3%)	Dominica [13.2 (13.9-14.6)]
Deaths	1980-2021	GBD Region (% in	Top 3	NA (NA%)	-

		All FBF increased)			
DALYs	Year 2021		Countries behind frontier n (%)	162 (79.4%)	Tokelau [22457.1 (16116.2-28258.2)]; Niue [20616.6 (16119.3-24986.6)]; Nigeria [19029.0 (12268.6-25707.9)]
DALYs	Year 2021	SDI Region (% in Top 100)	High (%)	1.00%	Kuwait [1539.5 (1222.1-1899.5)]
DALYs	Year 2021	SDI Region (% in Top 100)	High-middle (%)	23.00%	Niue [20616.6 (16119.3-24986.6)]; Azerbaijan [11901.3 (9466.4-15017.0)]; Palau [4566.0 (3441.9-6028.6)]
DALYs	Year 2021	SDI Region (% in Top 100)	Middle (%)	23.00%	Tokelau [22457.1 (16116.2-28258.2)]; Turkmenistan [11321.1 (9038.0-13907.4)]; Nauru [10070.2 (6954.1-14024.0)]
DALYs	Year 2021	SDI Region (% in Top 100)	Low-middle (%)	31.00%	Nigeria [19029.0 (12268.6-25707.9)]; Tajikistan [13341.3 (9629.4-17941.0)]; Zimbabwe [12059.8 (8004.4-16596.3)]
DALYs	Year 2021	SDI Region (% in Top 100)	Low (%)	22.00%	Papua New Guinea [17029.8 (10956.4-23635.7)]; South Sudan [16539.0 (6612.4-39214.1)]; Central African Republic [16107.5 (8042.9-26163.4)]
DALYs	Year 2021	GBD Region (% in Top 100)	Top 1	Oceania (17.0%)	Tokelau [22457.1 (16116.2-28258.2)]; Niue [20616.6 (16119.3-24986.6)]; Papua New Guinea [17029.8 (10956.4-23635.7)]
DALYs	Year 2021	GBD Region (% in Top 100)	Top 2	Eastern Sub-Saharan Africa (11.0%)	South Sudan [16539.0 (6612.4-39214.1)]; Comoros [6355.6 (2992.0-10618.2)]; Zambia [4455.5 (2047.2-7102.9)]
DALYs	Year 2021	GBD Region (% in Top 100)	Top 3	Western Sub-Saharan Africa (11.0%)	Nigeria [19029.0 (12268.6-25707.9)]; Burkina Faso [11049.4 (4957.2-17313.1)]; Guinea [10847.2 (4960.5-16938.1)]
DALYs	1990-2021		Countries behind frontier n (%)	8 (3.9%)	Niue [13186.5 (10733.6-15234.6)]; Tokelau [10361.4 (8054.4-11387.3)]; Dominica [1329.7 (1022.7-1829.5)]

DALYs	1990-2021	SDI Region (% in All FBF increased)	High (%)	0.00%	-
DALYs	1990-2021	SDI Region (% in All FBF increased)	High-middle (%)	50.00%	Niue [13186.5 (10733.6-15234.6)]; Dominica [1329.7 (1022.7-1829.5)]; Antigua and Barbuda [349.1 (211.2-440.2)]
DALYs	1990-2021	SDI Region (% in All FBF increased)	Middle (%)	50.00%	Tokelau [10361.4 (8054.4-11387.3)]; Grenada [810.0 (75.6-108.8)]; Saint Lucia [780.5 (1.8-707.5)]
DALYs	1990-2021	SDI Region (% in All FBF increased)	Low-middle (%)	0.00%	-
DALYs	1990-2021	SDI Region (% in All FBF increased)	Low (%)	0.00%	-
DALYs	1990-2021	GBD Region (% in All FBF increased)	Top 1	Caribbean (62.5%)	Dominica [1329.7 (1022.7-1829.5)]; Grenada [810.0 (75.6-108.8)]; Saint Lucia [780.5 (1.8-707.5)]
DALYs	1990-2021	GBD Region (% in All FBF increased)	Top 2	Oceania (25.0%)	Niue [13186.5 (10733.6-15234.6)]; Tokelau [10361.4 (8054.4-11387.3)]
DALYs	1990-2021	GBD Region (% in All FBF increased)	Top 3	Central Europe (12.5%)	Bosnia and Herzegovina [100.0 (113.0-253.7)]
YLDs	Year 2021		Countries behind frontier n (%)	120 (58.8%)	Pakistan [11.4 (5.7-18.7)]; India [9.0 (5.0-14.5)]; Kenya [8.5 (4.3-14.1)]
YLDs	Year 2021	SDI Region (% in Top 100)	High (%)	11.00%	Taiwan (Province of China) [4.4 (2.1-7.7)]; Bermuda [2.8 (1.4-4.7)]; Brunei Darussalam [2.7 (1.3-4.5)]
YLDs	Year 2021	SDI Region (% in Top 100)	High-middle (%)	27.00%	Northern Mariana Islands [6.1 (3.2-10.6)]; Guam [4.4 (2.0-7.3)]; Palau [3.9 (1.9-6.9)]
YLDs	Year 2021	SDI Region (% in	Middle (%)	20.00%	Botswana [8.3 (4.3-14.1)]; South Africa [7.6

		Top 100)			(4.4-11.7)]; Namibia [5.3 (2.2-9.6)]
YLDs	Year 2021	SDI Region (% in Top 100)	Low-middle (%)	24.00%	Pakistan [11.4 (5.7-18.7)]; India [9.0 (5.0-14.5)]; Kenya [8.5 (4.3-14.1)]
YLDs	Year 2021	SDI Region (% in Top 100)	Low (%)	18.00%	Nepal [6.6 (1.8-13.4)]; Guinea [5.3 (0.7-11.1)]; Eritrea [5.0 (0.5-11.2)]
YLDs	Year 2021	GBD Region (% in Top 100)	Top 1	Oceania (14.0%)	Northern Mariana Islands [6.1 (3.2-10.6)]; Nauru [4.6 (1.7-8.6)]; Papua New Guinea [4.5 (0.5-10.6)]
YLDs	Year 2021	GBD Region (% in Top 100)	Top 2	Western Sub-Saharan Africa (11.0%)	Nigeria [6.5 (2.9-11.8)]; Guinea [5.3 (0.7-11.1)]; Chad [4.2 (1.0-12.2)]
YLDs	Year 2021	GBD Region (% in Top 100)	Top 3	Caribbean (10.0%)	Bermuda [2.8 (1.4-4.7)]; Antigua and Barbuda [2.7 (1.3-4.6)]; Grenada [2.5 (0.8-4.6)]
YLDs	1990-2021		Countries behind frontier n (%)	3 (1.5%)	Poland [0.3 (0.4-0.8)]; Spain [0.1 (0.6-0.6)]; Cyprus [0.0 (0.4-0.6)]
YLDs	1990-2021	SDI Region (% in All FBF increased)	High (%)	66.70%	Poland [0.3 (0.4-0.8)]; Cyprus [0.0 (0.4-0.6)]
YLDs	1990-2021	SDI Region (% in All FBF increased)	High-middle (%)	33.30%	Spain [0.1 (0.6-0.6)]
YLDs	1990-2021	SDI Region (% in All FBF increased)	Middle (%)	0.00%	-
YLDs	1990-2021	SDI Region (% in All FBF increased)	Low-middle (%)	0.00%	-
YLDs	1990-2021	SDI Region (% in All FBF increased)	Low (%)	0.00%	-
YLDs	1990-2021	GBD Region (% in	Top 1	Western	Spain [0.1 (0.6-0.6)]; Cyprus [0.0 (0.4-0.6)]

		All FBF increased)		Europe (66.7%)	
YLDs	1990-2021	GBD Region (% in All FBF increased)	Top 2	Central Europe (33.3%)	Poland [0.3 (0.4-0.8)]
YLDs	1990-2021	GBD Region (% in All FBF increased)	Top 3	NA (NA%)	-
YLLs	Year 2021		Countries behind frontier n (%)	168 (82.4%)	Tokelau [22451.8 (16111.8-28253.1)]; Niue [20613.3 (16117.6-24984.4)]; Nigeria [19034.3 (12274.6-25715.7)]
YLLs	Year 2021	SDI Region (% in Top 100)	High (%)	1.00%	Kuwait [1537.3 (1220.1-1897.9)]
YLLs	Year 2021	SDI Region (% in Top 100)	High-middle (%)	22.00%	Niue [20613.3 (16117.6-24984.4)]; Azerbaijan [11898.2 (9462.9-15014.7)]; Palau [4561.8 (3438.9-6024.0)]
YLLs	Year 2021	SDI Region (% in Top 100)	Middle (%)	23.00%	Tokelau [22451.8 (16111.8-28253.1)]; Turkmenistan [11316.7 (9033.5-13903.1)]; Nauru [10280.5 (7158.0-14239.0)]
YLLs	Year 2021	SDI Region (% in Top 100)	Low-middle (%)	32.00%	Nigeria [19034.3 (12274.6-25715.7)]; Tajikistan [13348.8 (9635.4-17948.7)]; Zimbabwe [12220.9 (8157.5-16764.5)]
YLLs	Year 2021	SDI Region (% in Top 100)	Low (%)	22.00%	Papua New Guinea [17046.5 (10972.1-23654.7)]; South Sudan [16573.1 (6645.8-39255.3)]; Central African Republic [16102.9 (8033.3-26160.6)]
YLLs	Year 2021	GBD Region (% in Top 100)	Top 1	Oceania (17.0%)	Tokelau [22451.8 (16111.8-28253.1)]; Niue [20613.3 (16117.6-24984.4)]; Papua New Guinea [17046.5 (10972.1-23654.7)]
YLLs	Year 2021	GBD Region (% in Top 100)	Top 2	Eastern Sub-Saharan Africa (11.0%)	South Sudan [16573.1 (6645.8-39255.3)]; Comoros [6471.4 (3106.4-10735.6)]; United Republic of Tanzania [4687.2 (1954.5-7793.9)]

YLLs	Year 2021	GBD Region (% in Top 100)	Top 3	Western Sub-Saharan Africa (11.0%)	Nigeria [19034.3 (12274.6-25715.7)]; Chad [11550.8 (4233.0-20145.2)]; Burkina Faso [11013.5 (4921.4-17279.5)]
YLLs	1980-2021		Countries behind frontier n (%)	3 (1.5%)	Niue [11397.5 (10317.5-12144.9)]; Tokelau [2095.9 (13.3-3266.2)]; Dominica [1183.0 (1239.7-1302.2)]
YLLs	1980-2021	SDI Region (% in All FBF increased)	High (%)	0.00%	-
YLLs	1980-2021	SDI Region (% in All FBF increased)	High-middle (%)	66.70%	Niue [11397.5 (10317.5-12144.9)]; Dominica [1183.0 (1239.7-1302.2)]
YLLs	1980-2021	SDI Region (% in All FBF increased)	Middle (%)	33.30%	Tokelau [2095.9 (13.3-3266.2)]
YLLs	1980-2021	SDI Region (% in All FBF increased)	Low-middle (%)	0.00%	-
YLLs	1980-2021	SDI Region (% in All FBF increased)	Low (%)	0.00%	-
YLLs	1980-2021	GBD Region (% in All FBF increased)	Top 1	Oceania (66.7%)	Niue [11397.5 (10317.5-12144.9)]; Tokelau [2095.9 (13.3-3266.2)]
YLLs	1980-2021	GBD Region (% in All FBF increased)	Top 2	Caribbean (33.3%)	Dominica [1183.0 (1239.7-1302.2)]
YLLs	1980-2021	GBD Region (% in All FBF increased)	Top 3	NA (NA%)	-
Incidence	Year 2021		Countries behind	167 (81.9%)	Pakistan [7955.3 (6393.8-9652.6)]; India [6619.2

			frontier n (%)		(5506.8-8079.8)]; Botswana [6117.5 (4973.0-7441.1)]
Incidence	Year 2021	SDI Region (% in Top 100)	High (%)	12.00%	Taiwan (Province of China) [3351.3 (2597.8-4203.8)]; Bermuda [2048.3 (1615.4-2549.4)]; Kuwait [1887.8 (1458.8-2383.9)]
Incidence	Year 2021	SDI Region (% in Top 100)	High-middle (%)	24.00%	Northern Mariana Islands [4422.0 (3589.4-5319.0)]; Guam [3261.7 (2733.9-3976.6)]; Palau [2892.5 (2381.3-3486.3)]
Incidence	Year 2021	SDI Region (% in Top 100)	Middle (%)	21.00%	Botswana [6117.5 (4973.0-7441.1)]; South Africa [5617.6 (4836.4-6606.3)]; Namibia [3950.5 (3154.7-4952.3)]
Incidence	Year 2021	SDI Region (% in Top 100)	Low-middle (%)	25.00%	Pakistan [7955.3 (6393.8-9652.6)]; India [6619.2 (5506.8-8079.8)]; Kenya [6044.8 (4958.3-7223.4)]
Incidence	Year 2021	SDI Region (% in Top 100)	Low (%)	18.00%	Nepal [4668.9 (3228.2-6316.0)]; Guinea [3862.0 (2624.8-5272.1)]; Eritrea [3621.0 (2314.1-5136.5)]
Incidence	Year 2021	GBD Region (% in Top 100)	Top 1	Oceania (16.0%)	Northern Mariana Islands [4422.0 (3589.4-5319.0)]; Nauru [3514.3 (2754.1-4387.7)]; Papua New Guinea [3369.3 (2230.2-4601.4)]
Incidence	Year 2021	GBD Region (% in Top 100)	Top 2	Western Sub-Saharan Africa (11.0%)	Nigeria [4861.8 (3895.3-5890.4)]; Guinea [3862.0 (2624.8-5272.1)]; Chad [3137.1 (1714.6-4829.0)]
Incidence	Year 2021	GBD Region (% in Top 100)	Top 3	Caribbean (10.0%)	Bermuda [2048.3 (1615.4-2549.4)]; Antigua and Barbuda [1963.0 (1590.2-2387.3)]; Grenada [1871.5 (1434.4-2394.4)]
Incidence	1990-2021		Countries behind frontier n (%)	4 (2.0%)	Poland [164.2 (23.9-286.2)]; Spain [68.5 (112.6-209.1)]; Somalia [6.4 (1635.2-1854.4)]
Incidence	1990-2021	SDI Region (% in All FBF increased)	High (%)	50.00%	Poland [164.2 (23.9-286.2)]; Cyprus [2.5 (92.8-109.6)]
Incidence	1990-2021	SDI Region (% in All FBF	High-middle (%)	25.00%	Spain [68.5 (112.6-209.1)]

		increased)			
Incidence	1990-2021	SDI Region (% in All FBF increased)	Middle (%)	0.00%	-
Incidence	1990-2021	SDI Region (% in All FBF increased)	Low-middle (%)	0.00%	-
Incidence	1990-2021	SDI Region (% in All FBF increased)	Low (%)	25.00%	Somalia [6.4 (1635.2-1854.4)]
Incidence	1990-2021	GBD Region (% in All FBF increased)	Top 1	Western Europe (50.0%)	Spain [68.5 (112.6-209.1)]; Cyprus [2.5 (92.8-109.6)]
Incidence	1990-2021	GBD Region (% in All FBF increased)	Top 2	Central Europe (25.0%)	Poland [164.2 (23.9-286.2)]
Incidence	1990-2021	GBD Region (% in All FBF increased)	Top 3	Eastern Sub-Saharan Africa (25.0%)	Somalia [6.4 (1635.2-1854.4)]
				Lower respiratory infections |<20 years|Female	
Deaths	Year 2021		Countries behind frontier n (%)	169 (82.8%)	Nigeria [64.6 (41.9-88.3)]; Niue [64.3 (52.1-76.1)]; Papua New Guinea [62.0 (41.2-84.6)]
Deaths	Year 2021	SDI Region (% in Top 100)	High (%)	1.00%	Kuwait [5.4 (4.5-6.5)]

Deaths	Year 2021	SDI Region (% in Top 100)	High-middle (%)	23.00%	Niue [64.3 (52.1-76.1)]; Azerbaijan [35.3 (28.4-43.4)]; Palau [15.4 (12.0-19.5)]
Deaths	Year 2021	SDI Region (% in Top 100)	Middle (%)	24.00%	Tokelau [60.6 (45.5-75.5)]; Turkmenistan [39.1 (31.9-47.6)]; Uzbekistan [36.7 (29.8-44.3)]
Deaths	Year 2021	SDI Region (% in Top 100)	Low-middle (%)	32.00%	Nigeria [64.6 (41.9-88.3)]; Tajikistan [50.1 (36.9-66.4)]; Zimbabwe [41.8 (28.7-56.7)]
Deaths	Year 2021	SDI Region (% in Top 100)	Low (%)	20.00%	Papua New Guinea [62.0 (41.2-84.6)]; South Sudan [54.5 (20.6-129.3)]; Chad [54.1 (25.9-87.1)]
Deaths	Year 2021	GBD Region (% in Top 100)	Top 1	Oceania (17.0%)	Niue [64.3 (52.1-76.1)]; Papua New Guinea [62.0 (41.2-84.6)]; Tokelau [60.6 (45.5-75.5)]
Deaths	Year 2021	GBD Region (% in Top 100)	Top 2	Western Sub-Saharan Africa (11.0%)	Nigeria [64.6 (41.9-88.3)]; Chad [54.1 (25.9-87.1)]; Burkina Faso [38.3 (15.6-61.4)]
Deaths	Year 2021	GBD Region (% in Top 100)	Top 3	Eastern Sub-Saharan Africa (10.0%)	South Sudan [54.5 (20.6-129.3)]; Comoros [23.3 (13.0-36.2)]; United Republic of Tanzania [19.4 (9.9-30.0)]
Deaths	1980-2021		Countries behind frontier n (%)	3 (1.5%)	Niue [35.8 (33.9-37.3)]; Dominica [3.3 (3.2-3.9)]; Saint Lucia [1.0 (0.9-2.1)]
Deaths	1980-2021	SDI Region (% in All FBF increased)	High (%)	0.00%	-
Deaths	1980-2021	SDI Region (% in All FBF increased)	High-middle (%)	66.70%	Niue [35.8 (33.9-37.3)]; Dominica [3.3 (3.2-3.9)]
Deaths	1980-2021	SDI Region (% in All FBF increased)	Middle (%)	33.30%	Saint Lucia [1.0 (0.9-2.1)]
Deaths	1980-2021	SDI Region (% in All FBF	Low-middle (%)	0.00%	-

		increased)			
Deaths	1980-2021	SDI Region (% in All FBF increased)	Low (%)	0.00%	-
Deaths	1980-2021	GBD Region (% in All FBF increased)	Top 1	Caribbean (66.7%)	Dominica [3.3 (3.2-3.9)]; Saint Lucia [1.0 (0.9-2.1)]
Deaths	1980-2021	GBD Region (% in All FBF increased)	Top 2	Oceania (33.3%)	Niue [35.8 (33.9-37.3)]
Deaths	1980-2021	GBD Region (% in All FBF increased)	Top 3	NA (NA%)	-
DALYs	Year 2021		Countries behind frontier n (%)	169 (82.8%)	Nigeria [5699.8 (3696.1-7768.5)]; Niue [5606.1 (4538.9-6647.8)]; Papua New Guinea [5439.0 (3587.0-7471.5)]
DALYs	Year 2021	SDI Region (% in Top 100)	High (%)	2.00%	Kuwait [474.3 (390.1-571.0)]; Slovakia [279.1 (218.8-336.9)]
DALYs	Year 2021	SDI Region (% in Top 100)	High-middle (%)	23.00%	Niue [5606.1 (4538.9-6647.8)]; Azerbaijan [3130.4 (2521.2-3858.6)]; Palau [1308.6 (1003.5-1660.2)]
DALYs	Year 2021	SDI Region (% in Top 100)	Middle (%)	23.00%	Tokelau [5261.2 (3937.7-6570.7)]; Turkmenistan [3456.0 (2814.6-4204.0)]; Uzbekistan [3250.1 (2641.3-3935.8)]
DALYs	Year 2021	SDI Region (% in Top 100)	Low-middle (%)	32.00%	Nigeria [5699.8 (3696.1-7768.5)]; Tajikistan [4431.4 (3263.4-5885.1)]; Zimbabwe [3633.7 (2468.9-4947.7)]
DALYs	Year 2021	SDI Region (% in Top 100)	Low (%)	20.00%	Papua New Guinea [5439.0 (3587.0-7471.5)]; South Sudan [4775.7 (1781.7-11396.3)]; Central African Republic [4508.0 (2212.7-7294.3)]
DALYs	Year 2021	GBD Region (% in Top 100)	Top 1	Oceania (17.0%)	Niue [5606.1 (4538.9-6647.8)]; Papua New Guinea [5439.0 (3587.0-7471.5)]; Tokelau [5261.2 (3937.7-6570.7)]

DALYs	Year 2021	GBD Region (% in Top 100)	Top 2	Western Sub-Saharan Africa (11.0%)	Nigeria [5699.8 (3696.1-7768.5)]; Burkina Faso [3422.5 (1417.8-5452.6)]; Guinea [3304.8 (1497.5-5248.7)]
DALYs	Year 2021	GBD Region (% in Top 100)	Top 3	Eastern Sub-Saharan Africa (10.0%)	South Sudan [4775.7 (1781.7-11396.3)]; Comoros [2007.3 (1094.4-3143.5)]; United Republic of Tanzania [1628.6 (794.5-2558.9)]
DALYs	1990-2021		Countries behind frontier n (%)	8 (3.9%)	Niue [3470.1 (2952.3-3866.7)]; Tokelau [1909.8 (1641.8-1925.7)]; Saint Vincent and the Grenadines [232.6 (9.8-123.0)]
DALYs	1990-2021	SDI Region (% in All FBF increased)	High (%)	0.00%	-
DALYs	1990-2021	SDI Region (% in All FBF increased)	High-middle (%)	50.00%	Niue [3470.1 (2952.3-3866.7)]; Dominica [142.1 (137.6-194.5)]; Antigua and Barbuda [34.7 (16.0-70.8)]
DALYs	1990-2021	SDI Region (% in All FBF increased)	Middle (%)	50.00%	Tokelau [1909.8 (1641.8-1925.7)]; Saint Vincent and the Grenadines [232.6 (9.8-123.0)]; Saint Lucia [201.8 (3.3-160.8)]
DALYs	1990-2021	SDI Region (% in All FBF increased)	Low-middle (%)	0.00%	-
DALYs	1990-2021	SDI Region (% in All FBF increased)	Low (%)	0.00%	-
DALYs	1990-2021	GBD Region (% in All FBF increased)	Top 1	Caribbean (62.5%)	Saint Vincent and the Grenadines [232.6 (9.8-123.0)]; Saint Lucia [201.8 (3.3-160.8)]; Grenada [143.0 (94.8-139.9)]
DALYs	1990-2021	GBD Region (% in All FBF	Top 2	Oceania (25.0%)	Niue [3470.1 (2952.3-3866.7)]; Tokelau [1909.8 (1641.8-1925.7)]

		increased)			
DALYs	1990-2021	GBD Region (% in All FBF increased)	Top 3	Central Europe (12.5%)	Bosnia and Herzegovina [33.9 (27.2-64.7)]
YLDs	Year 2021		Countries behind frontier n (%)	139 (68.1%)	Pakistan [5.9 (3.1-9.6)]; Kenya [5.0 (2.7-7.7)]; India [4.9 (2.8-7.7)]
YLDs	Year 2021	SDI Region (% in Top 100)	High (%)	12.00%	Taiwan (Province of China) [2.7 (1.4-4.6)]; Lithuania [1.6 (0.8-2.7)]; Singapore [1.6 (0.8-2.6)]
YLDs	Year 2021	SDI Region (% in Top 100)	High-middle (%)	22.00%	China [2.8 (1.7-4.4)]; Northern Mariana Islands [2.4 (1.3-4.0)]; Azerbaijan [2.2 (1.2-3.6)]
YLDs	Year 2021	SDI Region (% in Top 100)	Middle (%)	22.00%	Botswana [4.3 (2.4-6.8)]; South Africa [4.1 (2.4-6.1)]; Namibia [3.1 (1.6-5.2)]
YLDs	Year 2021	SDI Region (% in Top 100)	Low-middle (%)	24.00%	Pakistan [5.9 (3.1-9.6)]; Kenya [5.0 (2.7-7.7)]; India [4.9 (2.8-7.7)]
YLDs	Year 2021	SDI Region (% in Top 100)	Low (%)	20.00%	Nepal [4.3 (1.2-8.1)]; United Republic of Tanzania [2.8 (0.8-5.5)]; Eritrea [2.8 (0.4-6.2)]
YLDs	Year 2021	GBD Region (% in Top 100)	Top 1	Oceania (15.0%)	Nauru [2.7 (1.4-4.4)]; Northern Mariana Islands [2.4 (1.3-4.0)]; Guam [2.2 (1.2-3.5)]
YLDs	Year 2021	GBD Region (% in Top 100)	Top 2	Western Sub-Saharan Africa (13.0%)	Nigeria [3.1 (1.3-5.4)]; Chad [2.8 (0.1-6.3)]; Ghana [2.7 (1.1-4.7)]
YLDs	Year 2021	GBD Region (% in Top 100)	Top 3	Eastern Sub-Saharan Africa (12.0%)	Kenya [5.0 (2.7-7.7)]; Djibouti [3.2 (1.1-5.9)]; Comoros [2.9 (0.9-5.6)]
YLDs	1990-2021		Countries behind frontier n (%)	2 (1.0%)	Spain [0.1 (0.1-0.2)]; Greece [0.0 (0.1-0.1)]; NA [NA]
YLDs	1990-2021	SDI Region (% in All FBF increased)	High (%)	0.00%	-

YLDs	1990-2021	SDI Region (% in All FBF increased)	High-middle (%)	100.00%	Spain [0.1 (0.1-0.2)]; Greece [0.0 (0.1-0.1)]
YLDs	1990-2021	SDI Region (% in All FBF increased)	Middle (%)	0.00%	-
YLDs	1990-2021	SDI Region (% in All FBF increased)	Low-middle (%)	0.00%	-
YLDs	1990-2021	SDI Region (% in All FBF increased)	Low (%)	0.00%	-
YLDs	1990-2021	GBD Region (% in All FBF increased)	Top 1	Western Europe (100.0%)	Spain [0.1 (0.1-0.2)]; Greece [0.0 (0.1-0.1)]
YLDs	1990-2021	GBD Region (% in All FBF increased)	Top 2	NA (NA%)	-
YLDs	1990-2021	GBD Region (% in All FBF increased)	Top 3	NA (NA%)	-
YLLs	Year 2021		Countries behind frontier n (%)	169 (82.8%)	Nigeria [5701.0 (3697.4-7771.3)]; Niue [5604.3 (4536.2-6645.5)]; Papua New Guinea [5545.5 (3692.2-7578.1)]
YLLs	Year 2021	SDI Region (% in Top 100)	High (%)	1.00%	Kuwait [472.9 (388.8-569.6)]
YLLs	Year 2021	SDI Region (% in Top 100)	High-middle (%)	23.00%	Niue [5604.3 (4536.2-6645.5)]; Azerbaijan [3128.2 (2519.1-3856.6)]; Palau [1306.9 (1001.3-1658.6)]
YLLs	Year 2021	SDI Region (% in Top 100)	Middle (%)	23.00%	Tokelau [5259.2 (3936.0-6568.6)]; Turkmenistan [3453.7 (2812.1-4202.1)]; Uzbekistan [3248.4 (2638.8-3934.6)]

YLLs	Year 2021	SDI Region (% in Top 100)	Low-middle (%)	32.00%	Nigeria [5701.0 (3697.4-7771.3)]; Tajikistan [4432.3 (3263.5-5886.6)]; Zimbabwe [3686.8 (2521.0-5002.8)]
YLLs	Year 2021	SDI Region (% in Top 100)	Low (%)	21.00%	Papua New Guinea [5545.5 (3692.2-7578.1)]; South Sudan [4786.4 (1790.3-11411.0)]; Chad [4767.2 (2291.6-7671.4)]
YLLs	Year 2021	GBD Region (% in Top 100)	Top 1	Oceania (17.0%)	Niue [5604.3 (4536.2-6645.5)]; Papua New Guinea [5545.5 (3692.2-7578.1)]; Tokelau [5259.2 (3936.0-6568.6)]
YLLs	Year 2021	GBD Region (% in Top 100)	Top 2	Western Sub-Saharan Africa (11.0%)	Nigeria [5701.0 (3697.4-7771.3)]; Chad [4767.2 (2291.6-7671.4)]; Burkina Faso [3426.8 (1422.9-5458.4)]
YLLs	Year 2021	GBD Region (% in Top 100)	Top 3	Eastern Sub-Saharan Africa (10.0%)	South Sudan [4786.4 (1790.3-11411.0)]; Comoros [2022.6 (1109.2-3160.3)]; United Republic of Tanzania [1691.9 (853.7-2624.5)]
YLLs	1980-2021		Countries behind frontier n (%)	4 (2.0%)	Niue [3090.9 (2931.2-3210.3)]; Dominica [282.5 (269.9-337.9)]; Saint Lucia [51.6 (43.7-217.5)]
YLLs	1980-2021	SDI Region (% in All FBF increased)	High (%)	0.00%	-
YLLs	1980-2021	SDI Region (% in All FBF increased)	High-middle (%)	50.00%	Niue [3090.9 (2931.2-3210.3)]; Dominica [282.5 (269.9-337.9)]
YLLs	1980-2021	SDI Region (% in All FBF increased)	Middle (%)	50.00%	Saint Lucia [51.6 (43.7-217.5)]; Cuba [10.3 (26.3-39.9)]
YLLs	1980-2021	SDI Region (% in All FBF increased)	Low-middle (%)	0.00%	-
YLLs	1980-2021	SDI Region (% in	Low (%)	0.00%	-

		All FBF increased)			
YLLs	1980-2021	GBD Region (% in All FBF increased)	Top 1	Caribbean (75.0%)	Dominica [282.5 (269.9-337.9)]; Saint Lucia [51.6 (43.7-217.5)]; Cuba [10.3 (26.3-39.9)]
YLLs	1980-2021	GBD Region (% in All FBF increased)	Top 2	Oceania (25.0%)	Niue [3090.9 (2931.2-3210.3)]
YLLs	1980-2021	GBD Region (% in All FBF increased)	Top 3	NA (NA%)	-
Incidence	Year 2021		Countries behind frontier n (%)	173 (84.8%)	Pakistan [4226.7 (3559.9-5056.9)]; India [3609.5 (3071.1-4274.9)]; Kenya [3601.9 (3121.2-4190.8)]
Incidence	Year 2021	SDI Region (% in Top 100)	High (%)	11.00%	Taiwan (Province of China) [1930.3 (1568.5-2365.3)]; Lithuania [1152.8 (877.3-1458.7)]; Kuwait [992.3 (815.6-1197.5)]
Incidence	Year 2021	SDI Region (% in Top 100)	High-middle (%)	20.00%	China [1978.7 (1613.1-2398.9)]; Northern Mariana Islands [1726.2 (1459.3-2027.1)]; Guam [1606.7 (1372.3-1860.8)]
Incidence	Year 2021	SDI Region (% in Top 100)	Middle (%)	22.00%	Botswana [3058.4 (2614.4-3674.9)]; South Africa [2854.5 (2492.6-3318.1)]; Namibia [2237.8 (1913.8-2649.1)]
Incidence	Year 2021	SDI Region (% in Top 100)	Low-middle (%)	26.00%	Pakistan [4226.7 (3559.9-5056.9)]; India [3609.5 (3071.1-4274.9)]; Kenya [3601.9 (3121.2-4190.8)]
Incidence	Year 2021	SDI Region (% in Top 100)	Low (%)	21.00%	Nepal [3127.1 (2402.1-4059.6)]; United Republic of Tanzania [2109.2 (1585.6-2700.8)]; Eritrea [2081.7 (1514.2-2867.9)]
Incidence	Year 2021	GBD Region (% in Top 100)	Top 1	Oceania (16.0%)	Nauru [1960.1 (1636.5-2292.7)]; Northern Mariana Islands [1726.2 (1459.3-2027.1)]; Guam [1606.7 (1372.3-1860.8)]
Incidence	Year 2021	GBD Region (% in	Top 2	Eastern	Kenya [3601.9 (3121.2-4190.8)]; Djibouti [2383.7

		Top 100)		Sub-Saharan Africa (13.0%)	(1906.4-2961.3)]; Comoros [2152.3 (1689.1-2697.7)]
Incidence	Year 2021	GBD Region (% in Top 100)	Top 3	Western Sub-Saharan Africa (13.0%)	Nigeria [2354.9 (1966.9-2792.8)]; Ghana [2027.3 (1675.3-2414.7)]; Chad [1950.9 (1326.1-2682.8)]
Incidence	1990-2021		Countries behind frontier n (%)	2 (1.0%)	Spain [28.1 (3.5-60.3)]; Greece [4.1 (24.3-25.8)]; NA [NA]
Incidence	1990-2021	SDI Region (% in All FBF increased)	High (%)	0.00%	-
Incidence	1990-2021	SDI Region (% in All FBF increased)	High-middle (%)	100.00%	Spain [28.1 (3.5-60.3)]; Greece [4.1 (24.3-25.8)]
Incidence	1990-2021	SDI Region (% in All FBF increased)	Middle (%)	0.00%	-
Incidence	1990-2021	SDI Region (% in All FBF increased)	Low-middle (%)	0.00%	-
Incidence	1990-2021	SDI Region (% in All FBF increased)	Low (%)	0.00%	-
Incidence	1990-2021	GBD Region (% in All FBF increased)	Top 1	Western Europe (100.0%)	Spain [28.1 (3.5-60.3)]; Greece [4.1 (24.3-25.8)]
Incidence	1990-2021	GBD Region (% in All FBF increased)	Top 2	NA (NA%)	-
Incidence	1990-2021	GBD Region (% in	Top 3	NA (NA%)	-

		All FBF increased)			
				Lower respiratory infections |<1 year|Male	
Deaths	Year 2021		Countries behind frontier n (%)	172 (84.3%)	Lesotho [990.4 (604.4-1395.6)]; Papua New Guinea [891.5 (594.6-1258.7)]; Tokelau [744.8 (434.2-966.5)]
Deaths	Year 2021	SDI Region (% in Top 100)	High (%)	1.00%	Kuwait [62.8 (49.4-78.8)]
Deaths	Year 2021	SDI Region (% in Top 100)	High-middle (%)	21.00%	Niue [601.7 (468.8-744.9)]; Azerbaijan [587.6 (455.7-745.2)]; Armenia [160.5 (125.8-203.7)]
Deaths	Year 2021	SDI Region (% in Top 100)	Middle (%)	23.00%	Tokelau [744.8 (434.2-966.5)]; Turkmenistan [495.6 (389.3-609.3)]; Uzbekistan [491.2 (395.3-599.6)]
Deaths	Year 2021	SDI Region (% in Top 100)	Low-middle (%)	33.00%	Lesotho [990.4 (604.4-1395.6)]; Nigeria [708.9 (500.2-953.6)]; Tajikistan [693.5 (425.8-991.8)]
Deaths	Year 2021	SDI Region (% in Top 100)	Low (%)	22.00%	Papua New Guinea [891.5 (594.6-1258.7)]; Central African Republic [567.8 (222.9-999.5)]; Chad [543.1 (199.5-929.3)]
Deaths	Year 2021	GBD Region (% in Top 100)	Top 1	Oceania (17.0%)	Papua New Guinea [891.5 (594.6-1258.7)]; Tokelau [744.8 (434.2-966.5)]; Niue [601.7 (468.8-744.9)]
Deaths	Year 2021	GBD Region (% in Top 100)	Top 2	Western Sub-Saharan Africa (12.0%)	Nigeria [708.9 (500.2-953.6)]; Chad [543.1 (199.5-929.3)]; Guinea [473.6 (212.0-833.1)]
Deaths	Year 2021	GBD Region (% in Top 100)	Top 3	Eastern Sub-Saharan Africa (9.0%)	South Sudan [392.7 (60.2-946.7)]; Madagascar [374.1 (173.5-628.5)]; Comoros [298.4 (148.7-490.6)]

Deaths	1980-2021		Countries behind frontier n (%)	5 (2.5%)	Niue [252.8 (240.2-243.0)]; Tokelau [110.3 (18.0-50.2)]; Lesotho [80.2 (83.2-85.7)]
Deaths	1980-2021	SDI Region (% in All FBF increased)	High (%)	0.00%	-
Deaths	1980-2021	SDI Region (% in All FBF increased)	High-middle (%)	40.00%	Niue [252.8 (240.2-243.0)]; Dominica [38.6 (32.7-62.6)]
Deaths	1980-2021	SDI Region (% in All FBF increased)	Middle (%)	20.00%	Tokelau [110.3 (18.0-50.2)]
Deaths	1980-2021	SDI Region (% in All FBF increased)	Low-middle (%)	40.00%	Lesotho [80.2 (83.2-85.7)]; Cabo Verde [15.8 (122.7-149.4)]
Deaths	1980-2021	SDI Region (% in All FBF increased)	Low (%)	0.00%	-
Deaths	1980-2021	GBD Region (% in All FBF increased)	Top 1	Oceania (40.0%)	Niue [252.8 (240.2-243.0)]; Tokelau [110.3 (18.0-50.2)]
Deaths	1980-2021	GBD Region (% in All FBF increased)	Top 2	Caribbean (20.0%)	Dominica [38.6 (32.7-62.6)]
Deaths	1980-2021	GBD Region (% in All FBF increased)	Top 3	Southern Sub-Saharan Africa (20.0%)	Lesotho [80.2 (83.2-85.7)]
DALYs	Year 2021		Countries behind frontier n (%)	172 (84.3%)	Lesotho [88828.0 (54217.5-125140.4)]; Papua New Guinea [79847.3 (53223.2-112734.3)]; Tokelau [66750.3 (38926.2-86611.0)]
DALYs	Year 2021	SDI Region (% in	High (%)	1.00%	Kuwait [5635.8 (4434.7-7072.2)]

		Top 100)			
DALYs	Year 2021	SDI Region (% in Top 100)	High-middle (%)	21.00%	Niue [53928.7 (42025.6-66780.4)]; Azerbaijan [52760.0 (40919.9-66907.7)]; Armenia [14417.6 (11311.0-18298.4)]
DALYs	Year 2021	SDI Region (% in Top 100)	Middle (%)	23.00%	Tokelau [66750.3 (38926.2-86611.0)]; Turkmenistan [44442.1 (34914.2-54637.8)]; Uzbekistan [44100.3 (35496.1-53839.4)]
DALYs	Year 2021	SDI Region (% in Top 100)	Low-middle (%)	33.00%	Lesotho [88828.0 (54217.5-125140.4)]; Nigeria [63726.3 (44984.2-85690.2)]; Tajikistan [62198.8 (38196.9-88964.2)]
DALYs	Year 2021	SDI Region (% in Top 100)	Low (%)	22.00%	Papua New Guinea [79847.3 (53223.2-112734.3)]; Central African Republic [51008.7 (20038.6-89729.5)]; Haiti [42881.5 (21541.1-69808.9)]
DALYs	Year 2021	GBD Region (% in Top 100)	Top 1	Oceania (17.0%)	Papua New Guinea [79847.3 (53223.2-112734.3)]; Tokelau [66750.3 (38926.2-86611.0)]; Niue [53928.7 (42025.6-66780.4)]
DALYs	Year 2021	GBD Region (% in Top 100)	Top 2	Western Sub-Saharan Africa (12.0%)	Nigeria [63726.3 (44984.2-85690.2)]; Guinea [42496.8 (18985.3-74767.8)]; Chad [40615.7 (9863.4-75191.2)]
DALYs	Year 2021	GBD Region (% in Top 100)	Top 3	Eastern Sub-Saharan Africa (9.0%)	South Sudan [34919.6 (5145.3-84580.2)]; Madagascar [33652.9 (15644.4-56481.5)]; Comoros [26706.1 (13282.6-43971.6)]
DALYs	1990-2021		Countries behind frontier n (%)	11 (5.4%)	Niue [33461.3 (27863.6-38644.9)]; Tokelau [31328.8 (15547.6-36496.1)]; Lesotho [13124.1 (5524.0-21680.8)]
DALYs	1990-2021	SDI Region (% in All FBF increased)	High (%)	0.00%	-
DALYs	1990-2021	SDI Region (% in	High-middle (%)	45.50%	Niue [33461.3 (27863.6-38644.9)]; Dominica [6674.5

		All FBF increased)			(4803.7-10515.5)]; Antigua and Barbuda [775.0 (32.2-1315.9)]
DALYs	1990-2021	SDI Region (% in All FBF increased)	Middle (%)	36.40%	Tokelau [31328.8 (15547.6-36496.1)]; Grenada [4878.9 (217.4-2354.6)]; Saint Lucia [3905.5 (705.9-3495.3)]
DALYs	1990-2021	SDI Region (% in All FBF increased)	Low-middle (%)	18.20%	Lesotho [13124.1 (5524.0-21680.8)]; Cabo Verde [1394.5 (8747.9-9210.3)]
DALYs	1990-2021	SDI Region (% in All FBF increased)	Low (%)	0.00%	-
DALYs	1990-2021	GBD Region (% in All FBF increased)	Top 1	Caribbean (45.5%)	Dominica [6674.5 (4803.7-10515.5)]; Grenada [4878.9 (217.4-2354.6)]; Saint Lucia [3905.5 (705.9-3495.3)]
DALYs	1990-2021	GBD Region (% in All FBF increased)	Top 2	Oceania (18.2%)	Niue [33461.3 (27863.6-38644.9)]; Tokelau [31328.8 (15547.6-36496.1)]
DALYs	1990-2021	GBD Region (% in All FBF increased)	Top 3	Central Europe (9.1%)	Bosnia and Herzegovina [211.6 (639.6-796.9)]
YLDs	Year 2021		Countries behind frontier n (%)	122 (59.8%)	Pakistan [27.3 (15.8-42.4)]; India [23.4 (14.3-36.9)]; Nepal [22.4 (11.6-37.3)]
YLDs	Year 2021	SDI Region (% in Top 100)	High (%)	4.00%	Poland [7.4 (4.5-11.4)]; Brunei Darussalam [5.9 (2.9-10.0)]; Singapore [4.4 (2.1-7.4)]
YLDs	Year 2021	SDI Region (% in Top 100)	High-middle (%)	19.00%	Azerbaijan [10.3 (5.4-17.1)]; Guam [6.0 (3.0-10.4)]; Romania [5.9 (3.1-10.0)]
YLDs	Year 2021	SDI Region (% in Top 100)	Middle (%)	18.00%	South Africa [12.8 (7.3-19.9)]; Botswana [10.7 (5.4-17.1)]; Gabon [9.4 (4.6-17.4)]
YLDs	Year 2021	SDI Region (% in Top 100)	Low-middle (%)	30.00%	Pakistan [27.3 (15.8-42.4)]; India [23.4 (14.3-36.9)]; Kenya [20.3 (12.4-32.3)]
YLDs	Year 2021	SDI Region (% in	Low (%)	29.00%	Nepal [22.4 (11.6-37.3)]; Sierra Leone [15.5

		Top 100)			(6.8-28.8)]; Guinea [15.4 (6.4-29.3)]
YLDs	Year 2021	GBD Region (% in Top 100)	Top 1	Western Sub-Saharan Africa (18.0%)	Nigeria [17.7 (10.4-28.3)]; Sierra Leone [15.5 (6.8-28.8)]; Guinea [15.4 (6.4-29.3)]
YLDs	Year 2021	GBD Region (% in Top 100)	Top 2	Oceania (15.0%)	Papua New Guinea [10.6 (4.0-19.9)]; Nauru [6.2 (2.5-11.7)]; Guam [6.0 (3.0-10.4)]
YLDs	Year 2021	GBD Region (% in Top 100)	Top 3	Eastern Sub-Saharan Africa (14.0%)	Kenya [20.3 (12.4-32.3)]; Djibouti [10.2 (4.4-18.5)]; Ethiopia [9.9 (3.5-18.7)]
YLDs	1990-2021		Countries behind frontier n (%)	2 (1.0%)	Somalia [0.2 (5.9-8.0)]; Mali [0.2 (3.0-4.2)]; NA [NA]
YLDs	1990-2021	SDI Region (% in All FBF increased)	High (%)	0.00%	-
YLDs	1990-2021	SDI Region (% in All FBF increased)	High-middle (%)	0.00%	-
YLDs	1990-2021	SDI Region (% in All FBF increased)	Middle (%)	0.00%	-
YLDs	1990-2021	SDI Region (% in All FBF increased)	Low-middle (%)	0.00%	-
YLDs	1990-2021	SDI Region (% in All FBF increased)	Low (%)	100.00%	Somalia [0.2 (5.9-8.0)]; Mali [0.2 (3.0-4.2)]
YLDs	1990-2021	GBD Region (% in All FBF increased)	Top 1	Eastern Sub-Saharan Africa	Somalia [0.2 (5.9-8.0)]

				(50.0%)	
YLDs	1990-2021	GBD Region (% in All FBF increased)	Top 2	Western Sub-Saharan Africa (50.0%)	Mali [0.2 (3.0-4.2)]
YLDs	1990-2021	GBD Region (% in All FBF increased)	Top 3	NA (NA%)	-
YLLs	Year 2021		Countries behind frontier n (%)	172 (84.3%)	Lesotho [88736.8 (54129.8-125051.5)]; Papua New Guinea [79997.2 (53368.6-112890.9)]; Tokelau [66748.5 (38925.3-86609.7)]
YLLs	Year 2021	SDI Region (% in Top 100)	High (%)	1.00%	Kuwait [5633.6 (4433.2-7069.6)]
YLLs	Year 2021	SDI Region (% in Top 100)	High-middle (%)	21.00%	Niue [53924.5 (42022.8-66776.1)]; Azerbaijan [52750.0 (40910.4-66900.8)]; Armenia [14398.8 (11292.0-18277.8)]
YLLs	Year 2021	SDI Region (% in Top 100)	Middle (%)	23.00%	Tokelau [66748.5 (38925.3-86609.7)]; Turkmenistan [44437.5 (34907.2-54634.9)]; Uzbekistan [44100.0 (35494.6-53838.9)]
YLLs	Year 2021	SDI Region (% in Top 100)	Low-middle (%)	33.00%	Lesotho [88736.8 (54129.8-125051.5)]; Nigeria [63651.4 (44915.6-85613.8)]; Tajikistan [62187.5 (38182.8-88952.1)]
YLLs	Year 2021	SDI Region (% in Top 100)	Low (%)	22.00%	Papua New Guinea [79997.2 (53368.6-112890.9)]; Central African Republic [50905.9 (19942.9-89614.6)]; Chad [48729.6 (17904.8-83390.9)]
YLLs	Year 2021	GBD Region (% in Top 100)	Top 1	Oceania (17.0%)	Papua New Guinea [79997.2 (53368.6-112890.9)]; Tokelau [66748.5 (38925.3-86609.7)]; Niue [53924.5 (42022.8-66776.1)]
YLLs	Year 2021	GBD Region (% in Top 100)	Top 2	Western Sub-Saharan Africa	Nigeria [63651.4 (44915.6-85613.8)]; Chad [48729.6 (17904.8-83390.9)]; Guinea [42481.6 (18976.5-74749.9)]

				(12.0%)	
YLLs	Year 2021	GBD Region (% in Top 100)	Top 3	Eastern Sub-Saharan Africa (9.0%)	South Sudan [35284.8 (5464.8-84988.1)]; Madagascar [33660.4 (15647.5-56489.1)]; Comoros [26774.4 (13339.3-44049.5)]
YLLs	1980-2021		Countries behind frontier n (%)	5 (2.5%)	Niue [22646.4 (21532.5-21782.2)]; Tokelau [9853.3 (1610.5-4444.6)]; Lesotho [7078.3 (7371.6-7538.8)]
YLLs	1980-2021	SDI Region (% in All FBF increased)	High (%)	0.00%	-
YLLs	1980-2021	SDI Region (% in All FBF increased)	High-middle (%)	40.00%	Niue [22646.4 (21532.5-21782.2)]; Dominica [3389.1 (2862.6-5542.7)]
YLLs	1980-2021	SDI Region (% in All FBF increased)	Middle (%)	20.00%	Tokelau [9853.3 (1610.5-4444.6)]
YLLs	1980-2021	SDI Region (% in All FBF increased)	Low-middle (%)	40.00%	Lesotho [7078.3 (7371.6-7538.8)]; Cabo Verde [1436.3 (10985.8-13418.5)]
YLLs	1980-2021	SDI Region (% in All FBF increased)	Low (%)	0.00%	-
YLLs	1980-2021	GBD Region (% in All FBF increased)	Top 1	Oceania (40.0%)	Niue [22646.4 (21532.5-21782.2)]; Tokelau [9853.3 (1610.5-4444.6)]
YLLs	1980-2021	GBD Region (% in All FBF increased)	Top 2	Caribbean (20.0%)	Dominica [3389.1 (2862.6-5542.7)]
YLLs	1980-2021	GBD Region (% in All FBF increased)	Top 3	Southern Sub-Saharan Africa	Lesotho [7078.3 (7371.6-7538.8)]

				(20.0%)	
Incidence	Year 2021		Countries behind frontier n (%)	171 (83.8%)	Pakistan [20132.2 (17097.8-23467.2)]; India [16601.4 (14018.0-19589.1)]; Nepal [15495.3 (12434.9-19425.4)]
Incidence	Year 2021	SDI Region (% in Top 100)	High (%)	4.00%	Poland [5157.4 (4580.1-5883.7)]; Brunei Darussalam [4222.1 (3401.8-4956.1)]; Singapore [3076.8 (2597.8-3648.9)]
Incidence	Year 2021	SDI Region (% in Top 100)	High-middle (%)	19.00%	Azerbaijan [7936.2 (6904.3-9204.7)]; Guam [4594.9 (3938.7-5292.2)]; Northern Mariana Islands [4425.7 (3742.5-5267.7)]
Incidence	Year 2021	SDI Region (% in Top 100)	Middle (%)	18.00%	South Africa [9379.3 (8117.9-10858.4)]; Botswana [8344.9 (7011.9-9866.0)]; Gabon [7132.4 (5703.8-8685.9)]
Incidence	Year 2021	SDI Region (% in Top 100)	Low-middle (%)	30.00%	Pakistan [20132.2 (17097.8-23467.2)]; India [16601.4 (14018.0-19589.1)]; Kenya [14840.7 (12762.7-17084.4)]
Incidence	Year 2021	SDI Region (% in Top 100)	Low (%)	29.00%	Nepal [15495.3 (12434.9-19425.4)]; Sierra Leone [12226.4 (9363.2-15580.2)]; Guinea [11736.4 (9091.0-14647.5)]
Incidence	Year 2021	GBD Region (% in Top 100)	Top 1	Western Sub-Saharan Africa (18.0%)	Nigeria [14138.8 (12384.8-15958.0)]; Sierra Leone [12226.4 (9363.2-15580.2)]; Guinea [11736.4 (9091.0-14647.5)]
Incidence	Year 2021	GBD Region (% in Top 100)	Top 2	Oceania (15.0%)	Papua New Guinea [8735.0 (7400.7-10379.8)]; Nauru [5005.4 (4182.6-6087.0)]; Guam [4594.9 (3938.7-5292.2)]
Incidence	Year 2021	GBD Region (% in Top 100)	Top 3	Eastern Sub-Saharan Africa (14.0%)	Kenya [14840.7 (12762.7-17084.4)]; Djibouti [7947.1 (6370.1-9635.4)]; United Republic of Tanzania [7201.4 (5589.7-9110.7)]
Incidence	1990-2021		Countries behind frontier n (%)	1 (0.5%)	Somalia [133.0 (1475.5-2120.6)]; NA [NA]; NA [NA]
Incidence	1990-2021	SDI Region (% in	High (%)	0.00%	-

		All FBF increased)			
Incidence	1990-2021	SDI Region (% in All FBF increased)	High-middle (%)	0.00%	-
Incidence	1990-2021	SDI Region (% in All FBF increased)	Middle (%)	0.00%	-
Incidence	1990-2021	SDI Region (% in All FBF increased)	Low-middle (%)	0.00%	-
Incidence	1990-2021	SDI Region (% in All FBF increased)	Low (%)	100.00%	Somalia [133.0 (1475.5-2120.6)]
Incidence	1990-2021	GBD Region (% in All FBF increased)	Top 1	Eastern Sub-Saharan Africa (100.0%)	Somalia [133.0 (1475.5-2120.6)]
Incidence	1990-2021	GBD Region (% in All FBF increased)	Top 2	NA (NA%)	-
Incidence	1990-2021	GBD Region (% in All FBF increased)	Top 3	NA (NA%)	-
			Lower respiratory infections|<5 years|Male		
Deaths	Year 2021		Countries behind frontier n (%)	171 (83.8%)	Chad [271.3 (145.5-413.4)]; Nigeria [263.8 (170.9-387.3)]; Papua New Guinea [232.8 (150.5-335.5)]
Deaths	Year 2021	SDI Region (% in Top 100)	High (%)	1.00%	Kuwait [15.8 (12.3-19.8)]

Deaths	Year 2021	SDI Region (% in Top 100)	High-middle (%)	20.00%	Niue [216.9 (178.6-262.9)]; Azerbaijan [130.0 (99.0-166.8)]; Palau [45.0 (33.3-60.3)]
Deaths	Year 2021	SDI Region (% in Top 100)	Middle (%)	24.00%	Tokelau [223.3 (135.4-278.0)]; Turkmenistan [155.4 (122.8-190.7)]; Uzbekistan [126.8 (101.8-155.6)]
Deaths	Year 2021	SDI Region (% in Top 100)	Low-middle (%)	32.00%	Nigeria [263.8 (170.9-387.3)]; Lesotho [220.2 (136.1-314.0)]; Tajikistan [184.9 (112.9-268.4)]
Deaths	Year 2021	SDI Region (% in Top 100)	Low (%)	23.00%	Chad [271.3 (145.5-413.4)]; Papua New Guinea [232.8 (150.5-335.5)]; Central African Republic [184.6 (71.9-326.6)]
Deaths	Year 2021	GBD Region (% in Top 100)	Top 1	Oceania (17.0%)	Papua New Guinea [232.8 (150.5-335.5)]; Tokelau [223.3 (135.4-278.0)]; Niue [216.9 (178.6-262.9)]
Deaths	Year 2021	GBD Region (% in Top 100)	Top 2	Western Sub-Saharan Africa (14.0%)	Chad [271.3 (145.5-413.4)]; Nigeria [263.8 (170.9-387.3)]; Guinea [167.3 (78.6-287.6)]
Deaths	Year 2021	GBD Region (% in Top 100)	Top 3	Eastern Sub-Saharan Africa (10.0%)	South Sudan [169.9 (68.1-334.0)]; Comoros [86.5 (47.3-135.4)]; Madagascar [76.0 (29.2-134.0)]
Deaths	1980-2021		Countries behind frontier n (%)	4 (2.0%)	Niue [109.4 (104.6-113.0)]; Tokelau [81.4 (45.2-69.9)]; Dominica [16.4 (16.1-19.3)]
Deaths	1980-2021	SDI Region (% in All FBF increased)	High (%)	0.00%	-
Deaths	1980-2021	SDI Region (% in All FBF increased)	High-middle (%)	50.00%	Niue [109.4 (104.6-113.0)]; Dominica [16.4 (16.1-19.3)]
Deaths	1980-2021	SDI Region (% in All FBF increased)	Middle (%)	50.00%	Tokelau [81.4 (45.2-69.9)]; Saint Lucia [0.1 (1.4-14.5)]
Deaths	1980-2021	SDI Region (% in	Low-middle (%)	0.00%	-

		All FBF increased)			
Deaths	1980-2021	SDI Region (% in All FBF increased)	Low (%)	0.00%	-
Deaths	1980-2021	GBD Region (% in All FBF increased)	Top 1	Caribbean (50.0%)	Dominica [16.4 (16.1-19.3)]; Saint Lucia [0.1 (1.4-14.5)]
Deaths	1980-2021	GBD Region (% in All FBF increased)	Top 2	Oceania (50.0%)	Niue [109.4 (104.6-113.0)]; Tokelau [81.4 (45.2-69.9)]
Deaths	1980-2021	GBD Region (% in All FBF increased)	Top 3	NA (NA%)	-
DALYs	Year 2021		Countries behind frontier n (%)	167 (81.9%)	Nigeria [23429.0 (15193.8-34356.5)]; Papua New Guinea [20813.2 (13464.7-29970.7)]; Tokelau [19849.3 (12015.4-24716.8)]
DALYs	Year 2021	SDI Region (% in Top 100)	High (%)	1.00%	Kuwait [1412.4 (1100.0-1770.1)]
DALYs	Year 2021	SDI Region (% in Top 100)	High-middle (%)	22.00%	Niue [19258.5 (15871.5-23317.9)]; Azerbaijan [11611.5 (8858.8-14889.1)]; Palau [4012.2 (2968.5-5380.0)]
DALYs	Year 2021	SDI Region (% in Top 100)	Middle (%)	24.00%	Tokelau [19849.3 (12015.4-24716.8)]; Turkmenistan [13871.5 (10961.2-17016.6)]; Uzbekistan [11336.2 (9105.2-13902.0)]
DALYs	Year 2021	SDI Region (% in Top 100)	Low-middle (%)	31.00%	Nigeria [23429.0 (15193.8-34356.5)]; Lesotho [19682.6 (12169.5-28050.3)]; Tajikistan [16494.4 (10073.1-23940.7)]
DALYs	Year 2021	SDI Region (% in Top 100)	Low (%)	22.00%	Papua New Guinea [20813.2 (13464.7-29970.7)]; Chad [18883.7 (7732.8-31409.7)]; Central African Republic [16386.8 (6369.5-29015.4)]
DALYs	Year 2021	GBD Region (% in	Top 1	Oceania	Papua New Guinea [20813.2 (13464.7-29970.7)]; Tokelau

		Top 100)		(17.0%)	[19849.3 (12015.4-24716.8)]; Niue [19258.5 (15871.5-23317.9)]
DALYs	Year 2021	GBD Region (% in Top 100)	Top 2	Western Sub-Saharan Africa (13.0%)	Nigeria [23429.0 (15193.8-34356.5)]; Chad [18883.7 (7732.8-31409.7)]; Guinea [14864.9 (7010.4-25543.3)]
DALYs	Year 2021	GBD Region (% in Top 100)	Top 3	Eastern Sub-Saharan Africa (10.0%)	South Sudan [14350.7 (5364.1-28954.1)]; Comoros [7699.8 (4205.2-12067.1)]; Madagascar [6793.8 (2610.2-11983.4)]
DALYs	1990-2021		Countries behind frontier n (%)	10 (4.9%)	Tokelau [13108.0 (7963.4-14837.3)]; Niue [10221.3 (9473.2-11306.2)]; Lesotho [2150.0 (716.0-4049.4)]
DALYs	1990-2021	SDI Region (% in All FBF increased)	High (%)	0.00%	-
DALYs	1990-2021	SDI Region (% in All FBF increased)	High-middle (%)	50.00%	Niue [10221.3 (9473.2-11306.2)]; Dominica [1369.1 (984.4-2026.1)]; Antigua and Barbuda [181.2 (42.9-336.7)]
DALYs	1990-2021	SDI Region (% in All FBF increased)	Middle (%)	40.00%	Tokelau [13108.0 (7963.4-14837.3)]; Grenada [1301.6 (184.9-520.6)]; Saint Lucia [786.8 (7.2-634.2)]
DALYs	1990-2021	SDI Region (% in All FBF increased)	Low-middle (%)	10.00%	Lesotho [2150.0 (716.0-4049.4)]
DALYs	1990-2021	SDI Region (% in All FBF increased)	Low (%)	0.00%	-
DALYs	1990-2021	GBD Region (% in All FBF increased)	Top 1	Caribbean (50.0%)	Dominica [1369.1 (984.4-2026.1)]; Grenada [1301.6 (184.9-520.6)]; Saint Lucia [786.8 (7.2-634.2)]
DALYs	1990-2021	GBD Region (% in	Top 2	Oceania	Tokelau [13108.0 (7963.4-14837.3)]; Niue [10221.3

		All FBF increased)		(20.0%)	(9473.2-11306.2)]
DALYs	1990-2021	GBD Region (% in All FBF increased)	Top 3	Central Europe (10.0%)	Bosnia and Herzegovina [75.1 (155.3-318.5)]
YLDs	Year 2021		Countries behind frontier n (%)	116 (56.9%)	Pakistan [13.0 (7.0-21.0)]; India [9.9 (5.3-16.3)]; Nepal [9.3 (3.4-17.8)]
YLDs	Year 2021	SDI Region (% in Top 100)	High (%)	10.00%	Taiwan (Province of China) [3.4 (1.4-6.4)]; Poland [3.4 (2.0-5.4)]; Lithuania [3.2 (1.2-6.2)]
YLDs	Year 2021	SDI Region (% in Top 100)	High-middle (%)	25.00%	Niue [5.3 (2.3-9.7)]; Northern Mariana Islands [5.2 (2.5-8.7)]; Palau [4.9 (2.3-8.5)]
YLDs	Year 2021	SDI Region (% in Top 100)	Middle (%)	19.00%	Botswana [8.4 (4.2-14.1)]; South Africa [7.7 (4.4-11.7)]; Brazil [4.9 (2.6-8.3)]
YLDs	Year 2021	SDI Region (% in Top 100)	Low-middle (%)	26.00%	Pakistan [13.0 (7.0-21.0)]; India [9.9 (5.3-16.3)]; Kenya [8.7 (4.4-14.8)]
YLDs	Year 2021	SDI Region (% in Top 100)	Low (%)	20.00%	Nepal [9.3 (3.4-17.8)]; Guinea [6.2 (1.4-13.5)]; Sierra Leone [5.6 (0.9-13.3)]
YLDs	Year 2021	GBD Region (% in Top 100)	Top 1	Oceania (15.0%)	Papua New Guinea [5.3 (0.5-12.0)]; Niue [5.3 (2.3-9.7)]; Northern Mariana Islands [5.2 (2.5-8.7)]
YLDs	Year 2021	GBD Region (% in Top 100)	Top 2	Western Sub-Saharan Africa (13.0%)	Nigeria [8.2 (4.1-14.3)]; Guinea [6.2 (1.4-13.5)]; Sierra Leone [5.6 (0.9-13.3)]
YLDs	Year 2021	GBD Region (% in Top 100)	Top 3	Eastern Sub-Saharan Africa (11.0%)	Kenya [8.7 (4.4-14.8)]; Djibouti [6.1 (2.1-11.4)]; Ethiopia [4.8 (0.7-9.9)]
YLDs	1990-2021		Countries behind frontier n (%)	3 (1.5%)	Poland [1.1 (0.4-1.4)]; Somalia [0.2 (5.0-7.5)]; Spain [0.1 (0.7-0.9)]
YLDs	1990-2021	SDI Region (% in All FBF	High (%)	33.30%	Poland [1.1 (0.4-1.4)]

		increased)			
YLDs	1990-2021	SDI Region (% in All FBF increased)	High-middle (%)	33.30%	Spain [0.1 (0.7-0.9)]
YLDs	1990-2021	SDI Region (% in All FBF increased)	Middle (%)	0.00%	-
YLDs	1990-2021	SDI Region (% in All FBF increased)	Low-middle (%)	0.00%	-
YLDs	1990-2021	SDI Region (% in All FBF increased)	Low (%)	33.30%	Somalia [0.2 (5.0-7.5)]
YLDs	1990-2021	GBD Region (% in All FBF increased)	Top 1	Central Europe (33.3%)	Poland [1.1 (0.4-1.4)]
YLDs	1990-2021	GBD Region (% in All FBF increased)	Top 2	Eastern Sub-Saharan Africa (33.3%)	Somalia [0.2 (5.0-7.5)]
YLDs	1990-2021	GBD Region (% in All FBF increased)	Top 3	Western Europe (33.3%)	Spain [0.1 (0.7-0.9)]
YLLs	Year 2021		Countries behind frontier n (%)	171 (83.8%)	Chad [23932.9 (12737.8-36506.0)]; Nigeria [23425.8 (15188.7-34350.8)]; Papua New Guinea [20790.0 (13435.9-29947.7)]
YLLs	Year 2021	SDI Region (% in Top 100)	High (%)	1.00%	Kuwait [1410.2 (1097.6-1767.6)]
YLLs	Year 2021	SDI Region (% in Top 100)	High-middle (%)	21.00%	Niue [19253.1 (15866.1-23311.7)]; Azerbaijan [11608.0 (8854.7-14886.0)]; Palau [4008.0 (2964.6-5375.1)]
YLLs	Year 2021	SDI Region (% in	Middle (%)	24.00%	Tokelau [19846.6 (12013.7-24713.6)]; Turkmenistan

		Top 100)			[13867.2 (10956.4-17013.6)]; Uzbekistan [11334.7 (9103.3-13902.1)]
YLLs	Year 2021	SDI Region (% in Top 100)	Low-middle (%)	31.00%	Nigeria [23425.8 (15188.7-34350.8)]; Lesotho [19684.0 (12168.1-28051.8)]; Tajikistan [16497.2 (10074.9-23944.3)]
YLLs	Year 2021	SDI Region (% in Top 100)	Low (%)	23.00%	Chad [23932.9 (12737.8-36506.0)]; Papua New Guinea [20790.0 (13435.9-29947.7)]; Central African Republic [16391.4 (6369.3-29019.5)]
YLLs	Year 2021	GBD Region (% in Top 100)	Top 1	Oceania (17.0%)	Papua New Guinea [20790.0 (13435.9-29947.7)]; Tokelau [19846.6 (12013.7-24713.6)]; Niue [19253.1 (15866.1-23311.7)]
YLLs	Year 2021	GBD Region (% in Top 100)	Top 2	Western Sub-Saharan Africa (14.0%)	Chad [23932.9 (12737.8-36506.0)]; Nigeria [23425.8 (15188.7-34350.8)]; Guinea [14856.7 (6999.7-25533.5)]
YLLs	Year 2021	GBD Region (% in Top 100)	Top 3	Eastern Sub-Saharan Africa (10.0%)	South Sudan [15108.5 (6059.7-29774.3)]; Comoros [7713.5 (4217.0-12082.9)]; Madagascar [6801.6 (2619.3-11991.7)]
YLLs	1980-2021		Countries behind frontier n (%)	3 (1.5%)	Niue [9675.0 (9211.3-10008.9)]; Tokelau [7133.3 (3939.7-6093.9)]; Dominica [1457.6 (1425.1-1716.4)]
YLLs	1980-2021	SDI Region (% in All FBF increased)	High (%)	0.00%	-
YLLs	1980-2021	SDI Region (% in All FBF increased)	High-middle (%)	66.70%	Niue [9675.0 (9211.3-10008.9)]; Dominica [1457.6 (1425.1-1716.4)]
YLLs	1980-2021	SDI Region (% in All FBF increased)	Middle (%)	33.30%	Tokelau [7133.3 (3939.7-6093.9)]
YLLs	1980-2021	SDI Region (% in	Low-middle (%)	0.00%	-

		All FBF increased)			
YLLs	1980-2021	SDI Region (% in All FBF increased)	Low (%)	0.00%	-
YLLs	1980-2021	GBD Region (% in All FBF increased)	Top 1	Oceania (66.7%)	Niue [9675.0 (9211.3-10008.9)]; Tokelau [7133.3 (3939.7-6093.9)]
YLLs	1980-2021	GBD Region (% in All FBF increased)	Top 2	Caribbean (33.3%)	Dominica [1457.6 (1425.1-1716.4)]
YLLs	1980-2021	GBD Region (% in All FBF increased)	Top 3	NA (NA%)	-
Incidence	Year 2021		Countries behind frontier n (%)	163 (79.9%)	Pakistan [9399.8 (7789.6-11185.5)]; India [7078.7 (5786.3-8727.6)]; Nepal [6508.7 (4895.4-8361.8)]
Incidence	Year 2021	SDI Region (% in Top 100)	High (%)	11.00%	Taiwan (Province of China) [2758.6 (2123.3-3548.3)]; Lithuania [2409.1 (1779.3-3249.7)]; Poland [2352.6 (1995.1-2748.4)]
Incidence	Year 2021	SDI Region (% in Top 100)	High-middle (%)	24.00%	Niue [3896.1 (3108.6-4823.0)]; Northern Mariana Islands [3886.8 (3118.9-4734.3)]; Palau [3664.2 (2980.5-4514.1)]
Incidence	Year 2021	SDI Region (% in Top 100)	Middle (%)	21.00%	Botswana [6101.9 (4911.6-7578.5)]; South Africa [5704.9 (4826.6-6699.7)]; Namibia [3700.6 (2820.6-4719.8)]
Incidence	Year 2021	SDI Region (% in Top 100)	Low-middle (%)	24.00%	Pakistan [9399.8 (7789.6-11185.5)]; India [7078.7 (5786.3-8727.6)]; Kenya [6043.6 (4960.2-7252.1)]
Incidence	Year 2021	SDI Region (% in Top 100)	Low (%)	20.00%	Nepal [6508.7 (4895.4-8361.8)]; Guinea [4597.0 (3371.5-5964.9)]; Sierra Leone [4229.9 (3021.1-5631.1)]
Incidence	Year 2021	GBD Region (% in	Top 1	Oceania	Papua New Guinea [4056.2 (2737.8-5600.5)]; Niue

		Top 100)		(15.0%)	[3896.1 (3108.6-4823.0)]; Northern Mariana Islands [3886.8 (3118.9-4734.3)]
Incidence	Year 2021	GBD Region (% in Top 100)	Top 2	Western Sub-Saharan Africa (13.0%)	Nigeria [6013.4 (5071.3-7093.9)]; Guinea [4597.0 (3371.5-5964.9)]; Sierra Leone [4229.9 (3021.1-5631.1)]
Incidence	Year 2021	GBD Region (% in Top 100)	Top 3	Eastern Sub-Saharan Africa (11.0%)	Kenya [6043.6 (4960.2-7252.1)]; Djibouti [4517.2 (3422.5-5859.8)]; United Republic of Tanzania [3506.0 (2509.7-4828.9)]
Incidence	1990-2021		Countries behind frontier n (%)	3 (1.5%)	Poland [653.3 (483.5-767.2)]; Spain [73.9 (94.1-273.3)]; Somalia [66.6 (1604.7-1704.7)]
Incidence	1990-2021	SDI Region (% in All FBF increased)	High (%)	33.30%	Poland [653.3 (483.5-767.2)]
Incidence	1990-2021	SDI Region (% in All FBF increased)	High-middle (%)	33.30%	Spain [73.9 (94.1-273.3)]
Incidence	1990-2021	SDI Region (% in All FBF increased)	Middle (%)	0.00%	-
Incidence	1990-2021	SDI Region (% in All FBF increased)	Low-middle (%)	0.00%	-
Incidence	1990-2021	SDI Region (% in All FBF increased)	Low (%)	33.30%	Somalia [66.6 (1604.7-1704.7)]
Incidence	1990-2021	GBD Region (% in All FBF increased)	Top 1	Central Europe (33.3%)	Poland [653.3 (483.5-767.2)]
Incidence	1990-2021	GBD Region (% in	Top 2	Eastern	Somalia [66.6 (1604.7-1704.7)]

		All FBF increased)		Sub-Saharan Africa (33.3%)	
Incidence	1990-2021	GBD Region (% in All FBF increased)	Top 3	Western Europe (33.3%)	Spain [73.9 (94.1-273.3)]
			Lower respiratory infections|<20 years|Male		
Deaths	Year 2021		Countries behind frontier n (%)	174 (85.3%)	Chad [103.6 (60.3-152.2)]; Nigeria [83.9 (54.6-122.1)]; Papua New Guinea [76.1 (50.5-107.9)]
Deaths	Year 2021	SDI Region (% in Top 100)	High (%)	1.00%	Kuwait [5.5 (4.4-6.7)]
Deaths	Year 2021	SDI Region (% in Top 100)	High-middle (%)	20.00%	Niue [57.0 (48.8-67.2)]; Azerbaijan [34.7 (26.8-44.4)]; Palau [11.8 (8.9-15.4)]
Deaths	Year 2021	SDI Region (% in Top 100)	Middle (%)	23.00%	Tokelau [52.7 (34.0-64.8)]; Turkmenistan [47.1 (38.0-57.2)]; Uzbekistan [42.8 (34.7-51.9)]
Deaths	Year 2021	SDI Region (% in Top 100)	Low-middle (%)	31.00%	Nigeria [83.9 (54.6-122.1)]; Lesotho [61.7 (41.2-85.7)]; Tajikistan [60.7 (37.9-87.1)]
Deaths	Year 2021	SDI Region (% in Top 100)	Low (%)	25.00%	Chad [103.6 (60.3-152.2)]; Papua New Guinea [76.1 (50.5-107.9)]; Central African Republic [56.6 (21.4-98.3)]
Deaths	Year 2021	GBD Region (% in Top 100)	Top 1	Oceania (18.0%)	Papua New Guinea [76.1 (50.5-107.9)]; Niue [57.0 (48.8-67.2)]; Tokelau [52.7 (34.0-64.8)]
Deaths	Year 2021	GBD Region (% in Top 100)	Top 2	Western Sub-Saharan Africa (16.0%)	Chad [103.6 (60.3-152.2)]; Nigeria [83.9 (54.6-122.1)]; Burkina Faso [54.0 (26.2-87.2)]
Deaths	Year 2021	GBD Region (% in Top 100)	Top 3	Eastern Sub-Saharan Africa	South Sudan [51.4 (21.5-99.5)]; Madagascar [28.9 (15.0-45.8)]; Comoros [25.9 (15.5-39.4)]

				(11.0%)	
Deaths	1980-2021		Countries behind frontier n (%)	5 (2.5%)	Niue [26.5 (24.1-29.5)]; Tokelau [11.7 (5.6-7.7)]; Dominica [3.6 (3.5-4.2)]
Deaths	1980-2021	SDI Region (% in All FBF increased)	High (%)	0.00%	-
Deaths	1980-2021	SDI Region (% in All FBF increased)	High-middle (%)	40.00%	Niue [26.5 (24.1-29.5)]; Dominica [3.6 (3.5-4.2)]
Deaths	1980-2021	SDI Region (% in All FBF increased)	Middle (%)	40.00%	Tokelau [11.7 (5.6-7.7)]; Saint Lucia [0.6 (1.3-3.9)]
Deaths	1980-2021	SDI Region (% in All FBF increased)	Low-middle (%)	20.00%	Cabo Verde [0.4 (10.7-11.0)]
Deaths	1980-2021	SDI Region (% in All FBF increased)	Low (%)	0.00%	-
Deaths	1980-2021	GBD Region (% in All FBF increased)	Top 1	Caribbean (40.0%)	Dominica [3.6 (3.5-4.2)]; Saint Lucia [0.6 (1.3-3.9)]
Deaths	1980-2021	GBD Region (% in All FBF increased)	Top 2	Oceania (40.0%)	Niue [26.5 (24.1-29.5)]; Tokelau [11.7 (5.6-7.7)]
Deaths	1980-2021	GBD Region (% in All FBF increased)	Top 3	Western Sub-Saharan Africa (20.0%)	Cabo Verde [0.4 (10.7-11.0)]
DALYs	Year 2021		Countries behind frontier n (%)	173 (84.8%)	Nigeria [7424.2 (4857.5-10796.1)]; Papua New Guinea [6794.3 (4516.8-9630.0)]; Chad [6683.8 (2846.5-10996.1)]

DALYs	Year 2021	SDI Region (% in Top 100)	High (%)	2.00%	Kuwait [473.6 (380.8-580.2)]; Slovakia [350.6 (264.3-440.9)]
DALYs	Year 2021	SDI Region (% in Top 100)	High-middle (%)	20.00%	Niue [4964.4 (4236.6-5853.7)]; Azerbaijan [3065.4 (2368.8-3918.0)]; Palau [1027.8 (773.0-1339.6)]
DALYs	Year 2021	SDI Region (% in Top 100)	Middle (%)	23.00%	Tokelau [4586.6 (2935.1-5645.4)]; Turkmenistan [4158.4 (3347.2-5052.2)]; Uzbekistan [3791.3 (3073.6-4603.9)]
DALYs	Year 2021	SDI Region (% in Top 100)	Low-middle (%)	31.00%	Nigeria [7424.2 (4857.5-10796.1)]; Lesotho [5423.6 (3587.4-7539.0)]; Tajikistan [5383.5 (3356.4-7735.6)]
DALYs	Year 2021	SDI Region (% in Top 100)	Low (%)	24.00%	Papua New Guinea [6794.3 (4516.8-9630.0)]; Chad [6683.8 (2846.5-10996.1)]; Central African Republic [4988.6 (1892.2-8712.3)]
DALYs	Year 2021	GBD Region (% in Top 100)	Top 1	Oceania (18.0%)	Papua New Guinea [6794.3 (4516.8-9630.0)]; Niue [4964.4 (4236.6-5853.7)]; Tokelau [4586.6 (2935.1-5645.4)]
DALYs	Year 2021	GBD Region (% in Top 100)	Top 2	Western Sub-Saharan Africa (15.0%)	Nigeria [7424.2 (4857.5-10796.1)]; Chad [6683.8 (2846.5-10996.1)]; Burkina Faso [4724.5 (2266.7-7656.0)]
DALYs	Year 2021	GBD Region (% in Top 100)	Top 3	Eastern Sub-Saharan Africa (11.0%)	South Sudan [4130.2 (1511.2-8370.9)]; Madagascar [2532.4 (1300.4-4027.9)]; Comoros [2283.3 (1362.3-3468.9)]
DALYs	1990-2021		Countries behind frontier n (%)	9 (4.4%)	Tokelau [2511.0 (1619.2-2670.5)]; Niue [2322.0 (2290.2-2364.1)]; Grenada [318.2 (0.5-59.7)]
DALYs	1990-2021	SDI Region (% in All FBF increased)	High (%)	0.00%	-
DALYs	1990-2021	SDI Region (% in All FBF increased)	High-middle (%)	55.60%	Niue [2322.0 (2290.2-2364.1)]; Dominica [157.7 (157.0-217.8)]; Bosnia and Herzegovina [23.0 (46.0-92.0)]

DALYs	1990-2021	SDI Region (% in All FBF increased)	Middle (%)	44.40%	Tokelau [2511.0 (1619.2-2670.5)]; Grenada [318.2 (0.5-59.7)]; Saint Lucia [201.4 (12.1-132.5)]
DALYs	1990-2021	SDI Region (% in All FBF increased)	Low-middle (%)	0.00%	-
DALYs	1990-2021	SDI Region (% in All FBF increased)	Low (%)	0.00%	-
DALYs	1990-2021	GBD Region (% in All FBF increased)	Top 1	Caribbean (55.6%)	Grenada [318.2 (0.5-59.7)]; Saint Lucia [201.4 (12.1-132.5)]; Dominica [157.7 (157.0-217.8)]
DALYs	1990-2021	GBD Region (% in All FBF increased)	Top 2	Oceania (22.2%)	Tokelau [2511.0 (1619.2-2670.5)]; Niue [2322.0 (2290.2-2364.1)]
DALYs	1990-2021	GBD Region (% in All FBF increased)	Top 3	Central Europe (11.1%)	Bosnia and Herzegovina [23.0 (46.0-92.0)]
YLDs	Year 2021		Countries behind frontier n (%)	141 (69.1%)	Pakistan [8.2 (4.7-12.5)]; Nepal [6.4 (2.7-11.1)]; India [5.7 (3.2-8.8)]
YLDs	Year 2021	SDI Region (% in Top 100)	High (%)	9.00%	Taiwan (Province of China) [2.4 (1.2-4.2)]; Lithuania [1.8 (0.9-3.1)]; Singapore [1.6 (0.9-2.7)]
YLDs	Year 2021	SDI Region (% in Top 100)	High-middle (%)	23.00%	China [2.5 (1.4-3.9)]; Niue [2.4 (1.2-4.0)]; Palau [2.4 (1.3-3.9)]
YLDs	Year 2021	SDI Region (% in Top 100)	Middle (%)	20.00%	Botswana [4.7 (2.6-7.6)]; South Africa [4.3 (2.6-6.4)]; Namibia [3.7 (1.9-6.1)]
YLDs	Year 2021	SDI Region (% in Top 100)	Low-middle (%)	23.00%	Pakistan [8.2 (4.7-12.5)]; India [5.7 (3.2-8.8)]; Kenya [5.4 (2.8-8.4)]
YLDs	Year 2021	SDI Region (% in Top 100)	Low (%)	25.00%	Nepal [6.4 (2.7-11.1)]; Guinea [3.5 (0.9-7.1)]; Sierra Leone [3.5 (1.0-7.0)]
YLDs	Year 2021	GBD Region (% in	Top 1	Oceania	Nauru [2.7 (1.3-4.7)]; Niue [2.4 (1.2-4.0)]; Palau [2.4

		Top 100)		(16.0%)	(1.3-3.9)]
YLDs	Year 2021	GBD Region (% in Top 100)	Top 2	Western Sub-Saharan Africa (14.0%)	Nigeria [4.0 (1.9-6.5)]; Guinea [3.5 (0.9-7.1)]; Sierra Leone [3.5 (1.0-7.0)]
YLDs	Year 2021	GBD Region (% in Top 100)	Top 3	Eastern Sub-Saharan Africa (13.0%)	Kenya [5.4 (2.8-8.4)]; Djibouti [3.8 (1.6-6.7)]; Eritrea [3.4 (0.9-7.0)]
YLDs	1990-2021		Countries behind frontier n (%)	3 (1.5%)	Somalia [0.1 (2.0-2.3)]; Poland [0.1 (0.2-0.3)]; Spain [0.1 (0.2-0.2)]
YLDs	1990-2021	SDI Region (% in All FBF increased)	High (%)	33.30%	Poland [0.1 (0.2-0.3)]
YLDs	1990-2021	SDI Region (% in All FBF increased)	High-middle (%)	33.30%	Spain [0.1 (0.2-0.2)]
YLDs	1990-2021	SDI Region (% in All FBF increased)	Middle (%)	0.00%	-
YLDs	1990-2021	SDI Region (% in All FBF increased)	Low-middle (%)	0.00%	-
YLDs	1990-2021	SDI Region (% in All FBF increased)	Low (%)	33.30%	Somalia [0.1 (2.0-2.3)]
YLDs	1990-2021	GBD Region (% in All FBF increased)	Top 1	Central Europe (33.3%)	Poland [0.1 (0.2-0.3)]
YLDs	1990-2021	GBD Region (% in All FBF	Top 2	Eastern Sub-Sahara	Somalia [0.1 (2.0-2.3)]

		increased)		n Africa (33.3%)	
YLDs	1990-2021	GBD Region (% in All FBF increased)	Top 3	Western Europe (33.3%)	Spain [0.1 (0.2-0.2)]
YLLs	Year 2021		Countries behind frontier n (%)	173 (84.8%)	Chad [9174.8 (5329.7-13495.6)]; Nigeria [7420.8 (4854.5-10791.6)]; Papua New Guinea [6808.5 (4526.4-9646.0)]
YLLs	Year 2021	SDI Region (% in Top 100)	High (%)	1.00%	Kuwait [472.5 (379.3-579.2)]
YLLs	Year 2021	SDI Region (% in Top 100)	High-middle (%)	20.00%	Niue [4962.3 (4234.7-5852.4)]; Azerbaijan [3063.1 (2366.7-3915.9)]; Palau [1025.6 (771.1-1337.6)]
YLLs	Year 2021	SDI Region (% in Top 100)	Middle (%)	23.00%	Tokelau [4585.1 (2933.3-5643.8)]; Turkmenistan [4156.8 (3345.1-5050.7)]; Uzbekistan [3789.8 (3072.2-4602.9)]
YLLs	Year 2021	SDI Region (% in Top 100)	Low-middle (%)	31.00%	Nigeria [7420.8 (4854.5-10791.6)]; Lesotho [5422.9 (3585.2-7540.0)]; Tajikistan [5383.7 (3356.1-7736.0)]
YLLs	Year 2021	SDI Region (% in Top 100)	Low (%)	25.00%	Chad [9174.8 (5329.7-13495.6)]; Papua New Guinea [6808.5 (4526.4-9646.0)]; Central African Republic [4990.4 (1893.5-8712.4)]
YLLs	Year 2021	GBD Region (% in Top 100)	Top 1	Oceania (18.0%)	Papua New Guinea [6808.5 (4526.4-9646.0)]; Niue [4962.3 (4234.7-5852.4)]; Tokelau [4585.1 (2933.3-5643.8)]
YLLs	Year 2021	GBD Region (% in Top 100)	Top 2	Western Sub-Saharan Africa (16.0%)	Chad [9174.8 (5329.7-13495.6)]; Nigeria [7420.8 (4854.5-10791.6)]; Burkina Faso [4747.5 (2280.5-7686.4)]
YLLs	Year 2021	GBD Region (% in Top 100)	Top 3	Eastern Sub-Saharan Africa (11.0%)	South Sudan [4459.9 (1812.9-8730.2)]; Madagascar [2539.7 (1306.9-4035.4)]; Comoros [2282.0 (1358.4-3467.7)]

YLLs	1980-2021		Countries behind frontier n (%)	5 (2.5%)	Niue [2306.3 (2068.8-2561.0)]; Tokelau [970.3 (412.6-607.1)]; Dominica [314.8 (303.4-366.7)]
YLLs	1980-2021	SDI Region (% in All FBF increased)	High (%)	0.00%	-
YLLs	1980-2021	SDI Region (% in All FBF increased)	High-middle (%)	40.00%	Niue [2306.3 (2068.8-2561.0)]; Dominica [314.8 (303.4-366.7)]
YLLs	1980-2021	SDI Region (% in All FBF increased)	Middle (%)	40.00%	Tokelau [970.3 (412.6-607.1)]; Saint Lucia [39.2 (103.3-366.8)]
YLLs	1980-2021	SDI Region (% in All FBF increased)	Low-middle (%)	20.00%	Cabo Verde [19.6 (970.1-977.9)]
YLLs	1980-2021	SDI Region (% in All FBF increased)	Low (%)	0.00%	-
YLLs	1980-2021	GBD Region (% in All FBF increased)	Top 1	Caribbean (40.0%)	Dominica [314.8 (303.4-366.7)]; Saint Lucia [39.2 (103.3-366.8)]
YLLs	1980-2021	GBD Region (% in All FBF increased)	Top 2	Oceania (40.0%)	Niue [2306.3 (2068.8-2561.0)]; Tokelau [970.3 (412.6-607.1)]
YLLs	1980-2021	GBD Region (% in All FBF increased)	Top 3	Western Sub-Saharan Africa (20.0%)	Cabo Verde [19.6 (970.1-977.9)]
Incidence	Year 2021		Countries behind frontier n (%)	178 (87.3%)	Pakistan [5809.7 (5045.5-6819.5)]; Nepal [4514.9 (3622.1-5575.0)]; India [4093.5 (3420.4-4923.3)]
Incidence	Year 2021	SDI Region (% in Top 100)	High (%)	9.00%	Taiwan (Province of China) [1727.4 (1384.9-2152.7)]; Lithuania [1295.0 (1004.6-1673.8)]; Estonia [1091.3

					(842.3-1395.4)]
Incidence	Year 2021	SDI Region (% in Top 100)	High-middle (%)	23.00%	Niue [1725.4 (1470.0-2029.6)]; China [1711.7 (1380.2-2102.4)]; Palau [1708.8 (1459.6-2022.2)]
Incidence	Year 2021	SDI Region (% in Top 100)	Middle (%)	19.00%	Botswana [3372.2 (2801.8-4023.8)]; South Africa [2991.8 (2605.9-3500.3)]; Namibia [2655.6 (2213.2-3221.5)]
Incidence	Year 2021	SDI Region (% in Top 100)	Low-middle (%)	24.00%	Pakistan [5809.7 (5045.5-6819.5)]; India [4093.5 (3420.4-4923.3)]; Kenya [3819.8 (3263.5-4411.8)]
Incidence	Year 2021	SDI Region (% in Top 100)	Low (%)	25.00%	Nepal [4514.9 (3622.1-5575.0)]; Guinea [2603.3 (1988.3-3268.1)]; Sierra Leone [2565.3 (1970.5-3200.2)]
Incidence	Year 2021	GBD Region (% in Top 100)	Top 1	Oceania (16.0%)	Nauru [1985.3 (1638.4-2365.1)]; Niue [1725.4 (1470.0-2029.6)]; Palau [1708.8 (1459.6-2022.2)]
Incidence	Year 2021	GBD Region (% in Top 100)	Top 2	Western Sub-Saharan Africa (14.0%)	Nigeria [2917.5 (2493.2-3417.9)]; Guinea [2603.3 (1988.3-3268.1)]; Sierra Leone [2565.3 (1970.5-3200.2)]
Incidence	Year 2021	GBD Region (% in Top 100)	Top 3	Eastern Sub-Saharan Africa (13.0%)	Kenya [3819.8 (3263.5-4411.8)]; Djibouti [2833.0 (2306.2-3450.1)]; Eritrea [2563.7 (1955.1-3269.3)]
Incidence	1990-2021		Countries behind frontier n (%)	3 (1.5%)	Spain [32.2 (0.2-70.2)]; Somalia [30.5 (413.1-544.3)]; Poland [17.8 (77.6-87.7)]
Incidence	1990-2021	SDI Region (% in All FBF increased)	High (%)	33.30%	Poland [17.8 (77.6-87.7)]
Incidence	1990-2021	SDI Region (% in All FBF increased)	High-middle (%)	33.30%	Spain [32.2 (0.2-70.2)]
Incidence	1990-2021	SDI Region (% in All FBF	Middle (%)	0.00%	-

		increased)			
Incidence	1990-2021	SDI Region (% in All FBF increased)	Low-middle (%)	0.00%	-
Incidence	1990-2021	SDI Region (% in All FBF increased)	Low (%)	33.30%	Somalia [30.5 (413.1-544.3)]
Incidence	1990-2021	GBD Region (% in All FBF increased)	Top 1	Central Europe (33.3%)	Poland [17.8 (77.6-87.7)]
Incidence	1990-2021	GBD Region (% in All FBF increased)	Top 2	Eastern Sub-Saharan Africa (33.3%)	Somalia [30.5 (413.1-544.3)]
Incidence	1990-2021	GBD Region (% in All FBF increased)	Top 3	Western Europe (33.3%)	Spain [32.2 (0.2-70.2)]
			Lower respiratory infections|<1 year|Both		
Deaths	Year 2021		Countries behind frontier n (%)	171 (83.8%)	Papua New Guinea [806.4 (574.4-1088.3)]; Lesotho [776.0 (531.3-1053.2)]; Tokelau [775.5 (452.7-980.4)]
Deaths	Year 2021	SDI Region (% in Top 100)	High (%)	1.00%	Kuwait [64.0 (50.7-78.1)]
Deaths	Year 2021	SDI Region (% in Top 100)	High-middle (%)	23.00%	Niue [694.4 (579.5-812.8)]; Azerbaijan [609.0 (484.4-752.8)]; Palau [168.8 (124.4-220.6)]
Deaths	Year 2021	SDI Region (% in Top 100)	Middle (%)	23.00%	Tokelau [775.5 (452.7-980.4)]; Uzbekistan [468.5 (377.3-572.6)]; Turkmenistan [455.2 (359.6-561.4)]
Deaths	Year 2021	SDI Region (% in Top 100)	Low-middle (%)	32.00%	Lesotho [776.0 (531.3-1053.2)]; Tajikistan [630.5 (420.9-884.3)]; Nigeria [610.6 (442.2-794.0)]
Deaths	Year 2021	SDI Region (% in	Low (%)	21.00%	Papua New Guinea [806.4 (574.4-1088.3)]; Central

		Top 100)			African Republic [543.6 (270.0-862.6)]; Haiti [426.5 (227.3-640.6)]
Deaths	Year 2021	GBD Region (% in Top 100)	Top 1	Oceania (17.0%)	Papua New Guinea [806.4 (574.4-1088.3)]; Tokelau [775.5 (452.7-980.4)]; Niue [694.4 (579.5-812.8)]
Deaths	Year 2021	GBD Region (% in Top 100)	Top 2	Western Sub-Saharan Africa (12.0%)	Nigeria [610.6 (442.2-794.0)]; Guinea [386.6 (186.8-609.8)]; Chad [366.6 (102.5-674.8)]
Deaths	Year 2021	GBD Region (% in Top 100)	Top 3	Caribbean (9.0%)	Haiti [426.5 (227.3-640.6)]; Dominica [131.4 (88.0-192.7)]; Guyana [116.1 (79.9-164.7)]
Deaths	1980-2021		Countries behind frontier n (%)	4 (2.0%)	Tokelau [375.5 (205.5-409.2)]; Niue [310.9 (307.3-309.4)]; Dominica [37.8 (34.8-57.1)]
Deaths	1980-2021	SDI Region (% in All FBF increased)	High (%)	0.00%	-
Deaths	1980-2021	SDI Region (% in All FBF increased)	High-middle (%)	50.00%	Niue [310.9 (307.3-309.4)]; Dominica [37.8 (34.8-57.1)]
Deaths	1980-2021	SDI Region (% in All FBF increased)	Middle (%)	25.00%	Tokelau [375.5 (205.5-409.2)]
Deaths	1980-2021	SDI Region (% in All FBF increased)	Low-middle (%)	25.00%	Cabo Verde [3.2 (121.6-154.8)]
Deaths	1980-2021	SDI Region (% in All FBF increased)	Low (%)	0.00%	-
Deaths	1980-2021	GBD Region (% in All FBF increased)	Top 1	Oceania (50.0%)	Tokelau [375.5 (205.5-409.2)]; Niue [310.9 (307.3-309.4)]
Deaths	1980-2021	GBD Region (% in	Top 2	Caribbean	Dominica [37.8 (34.8-57.1)]

		All FBF increased)		(25.0%)	
Deaths	1980-2021	GBD Region (% in All FBF increased)	Top 3	Western Sub-Saharan Africa (25.0%)	Cabo Verde [3.2 (121.6-154.8)]
DALYs	Year 2021		Countries behind frontier n (%)	172 (84.3%)	Papua New Guinea [72239.4 (51443.2-97537.4)]; Tokelau [69513.2 (40596.2-87887.4)]; Lesotho [69506.6 (47577.8-94331.1)]
DALYs	Year 2021	SDI Region (% in Top 100)	High (%)	2.00%	Kuwait [5752.5 (4551.2-7014.3)]; Slovakia [4079.0 (3047.2-5137.1)]
DALYs	Year 2021	SDI Region (% in Top 100)	High-middle (%)	23.00%	Niue [62231.8 (51927.3-72853.0)]; Azerbaijan [54681.3 (43491.6-67588.1)]; Palau [15141.8 (11163.0-19790.2)]
DALYs	Year 2021	SDI Region (% in Top 100)	Middle (%)	23.00%	Tokelau [69513.2 (40596.2-87887.4)]; Uzbekistan [42052.7 (33868.0-51392.2)]; Turkmenistan [40824.0 (32252.3-50353.0)]
DALYs	Year 2021	SDI Region (% in Top 100)	Low-middle (%)	31.00%	Lesotho [69506.6 (47577.8-94331.1)]; Tajikistan [56551.9 (37749.6-79335.5)]; Nigeria [54870.7 (39760.6-71318.4)]
DALYs	Year 2021	SDI Region (% in Top 100)	Low (%)	21.00%	Papua New Guinea [72239.4 (51443.2-97537.4)]; Central African Republic [48613.2 (24074.3-77226.7)]; Haiti [38267.0 (20387.5-57443.0)]
DALYs	Year 2021	GBD Region (% in Top 100)	Top 1	Oceania (17.0%)	Papua New Guinea [72239.4 (51443.2-97537.4)]; Tokelau [69513.2 (40596.2-87887.4)]; Niue [62231.8 (51927.3-72853.0)]
DALYs	Year 2021	GBD Region (% in Top 100)	Top 2	Western Sub-Saharan Africa (12.0%)	Nigeria [54870.7 (39760.6-71318.4)]; Guinea [34786.1 (16844.7-54783.9)]; Chad [31224.1 (7661.5-58745.4)]
DALYs	Year 2021	GBD Region (% in Top 100)	Top 3	Caribbean (9.0%)	Haiti [38267.0 (20387.5-57443.0)]; Dominica [11791.9 (7893.7-17292.5)]; Guyana [10419.7 (7170.7-14781.2)]

DALYs	1990-2021		Countries behind frontier n (%)	9 (4.4%)	Tokelau [45332.3 (24833.9-54507.6)]; Niue [35380.9 (31223.8-38534.6)]; Dominica [6313.7 (4578.0-9463.7)]
DALYs	1990-2021	SDI Region (% in All FBF increased)	High (%)	0.00%	-
DALYs	1990-2021	SDI Region (% in All FBF increased)	High-middle (%)	44.40%	Niue [35380.9 (31223.8-38534.6)]; Dominica [6313.7 (4578.0-9463.7)]; Antigua and Barbuda [928.5 (309.4-1407.8)]
DALYs	1990-2021	SDI Region (% in All FBF increased)	Middle (%)	44.40%	Tokelau [45332.3 (24833.9-54507.6)]; Saint Lucia [3690.9 (440.7-3374.0)]; Grenada [2357.0 (74.8-2074.6)]
DALYs	1990-2021	SDI Region (% in All FBF increased)	Low-middle (%)	11.10%	Lesotho [5142.4 (74.9-9368.1)]
DALYs	1990-2021	SDI Region (% in All FBF increased)	Low (%)	0.00%	-
DALYs	1990-2021	GBD Region (% in All FBF increased)	Top 1	Caribbean (55.6%)	Dominica [6313.7 (4578.0-9463.7)]; Saint Lucia [3690.9 (440.7-3374.0)]; Grenada [2357.0 (74.8-2074.6)]
DALYs	1990-2021	GBD Region (% in All FBF increased)	Top 2	Oceania (22.2%)	Tokelau [45332.3 (24833.9-54507.6)]; Niue [35380.9 (31223.8-38534.6)]
DALYs	1990-2021	GBD Region (% in All FBF increased)	Top 3	Central Europe (11.1%)	Bosnia and Herzegovina [54.5 (387.1-613.9)]
YLDs	Year 2021		Countries behind frontier n (%)	120 (58.8%)	Pakistan [25.6 (15.2-40.3)]; India [22.0 (13.5-34.6)]; Nepal [19.4 (10.2-33.4)]
YLDs	Year 2021	SDI Region (% in Top 100)	High (%)	7.00%	Poland [6.4 (3.9-9.8)]; Brunei Darussalam [5.4 (2.9-8.6)]; Singapore [4.1 (2.2-6.8)]
YLDs	Year 2021	SDI Region (% in	High-middle (%)	20.00%	Azerbaijan [10.2 (5.8-16.4)]; Guam [5.9 (3.4-9.2)];

		Top 100)			Romania [5.5 (3.0-9.2)]
YLDs	Year 2021	SDI Region (% in Top 100)	Middle (%)	18.00%	South Africa [13.0 (7.7-20.9)]; Botswana [10.4 (5.8-17.0)]; Gabon [8.4 (4.4-14.3)]
YLDs	Year 2021	SDI Region (% in Top 100)	Low-middle (%)	29.00%	Pakistan [25.6 (15.2-40.3)]; India [22.0 (13.5-34.6)]; Kenya [19.4 (11.7-30.4)]
YLDs	Year 2021	SDI Region (% in Top 100)	Low (%)	26.00%	Nepal [19.4 (10.2-33.4)]; Guinea [13.1 (5.1-26.1)]; Sierra Leone [12.4 (4.9-23.4)]
YLDs	Year 2021	GBD Region (% in Top 100)	Top 1	Western Sub-Saharan Africa (17.0%)	Nigeria [16.4 (9.6-26.4)]; Guinea [13.1 (5.1-26.1)]; Mauritania [12.5 (6.3-22.1)]
YLDs	Year 2021	GBD Region (% in Top 100)	Top 2	Oceania (16.0%)	Papua New Guinea [8.8 (2.8-16.8)]; Guam [5.9 (3.4-9.2)]; Nauru [5.7 (2.5-10.2)]
YLDs	Year 2021	GBD Region (% in Top 100)	Top 3	Eastern Sub-Saharan Africa (12.0%)	Kenya [19.4 (11.7-30.4)]; Djibouti [8.7 (3.9-15.6)]; Comoros [8.3 (3.2-15.0)]
YLDs	1990-2021		Countries behind frontier n (%)	2 (1.0%)	Somalia [0.2 (4.9-7.4)]; France [0.0 (0.4-0.5)]; NA [NA]
YLDs	1990-2021	SDI Region (% in All FBF increased)	High (%)	50.00%	France [0.0 (0.4-0.5)]
YLDs	1990-2021	SDI Region (% in All FBF increased)	High-middle (%)	0.00%	-
YLDs	1990-2021	SDI Region (% in All FBF increased)	Middle (%)	0.00%	-
YLDs	1990-2021	SDI Region (% in All FBF increased)	Low-middle (%)	0.00%	-

YLDs	1990-2021	SDI Region (% in All FBF increased)	Low (%)	50.00%	Somalia [0.2 (4.9-7.4)]
YLDs	1990-2021	GBD Region (% in All FBF increased)	Top 1	Eastern Sub-Saharan Africa (50.0%)	Somalia [0.2 (4.9-7.4)]
YLDs	1990-2021	GBD Region (% in All FBF increased)	Top 2	Western Europe (50.0%)	France [0.0 (0.4-0.5)]
YLDs	1990-2021	GBD Region (% in All FBF increased)	Top 3	NA (NA%)	-
YLLs	Year 2021		Countries behind frontier n (%)	172 (84.3%)	Papua New Guinea [72341.5 (51533.9-97646.1)]; Lesotho [69511.3 (47574.5-94342.9)]; Tokelau [69505.6 (40587.1-87878.0)]
YLLs	Year 2021	SDI Region (% in Top 100)	High (%)	1.00%	Kuwait [5750.6 (4549.4-7012.8)]
YLLs	Year 2021	SDI Region (% in Top 100)	High-middle (%)	23.00%	Niue [62226.9 (51921.3-72845.6)]; Azerbaijan [54673.4 (43483.7-67582.2)]; Palau [15134.7 (11156.7-19782.6)]
YLLs	Year 2021	SDI Region (% in Top 100)	Middle (%)	23.00%	Tokelau [69505.6 (40587.1-87878.0)]; Uzbekistan [42049.9 (33862.5-51388.0)]; Turkmenistan [40810.8 (32241.0-50338.6)]
YLLs	Year 2021	SDI Region (% in Top 100)	Low-middle (%)	32.00%	Lesotho [69511.3 (47574.5-94342.9)]; Tajikistan [56556.8 (37754.3-79337.5)]; Nigeria [54940.5 (39829.0-71395.0)]
YLLs	Year 2021	SDI Region (% in Top 100)	Low (%)	21.00%	Papua New Guinea [72341.5 (51533.9-97646.1)]; Central African Republic [48705.9 (24158.3-77324.1)]; Haiti [38309.7 (20423.0-57494.8)]
YLLs	Year 2021	GBD Region (% in Top 100)	Top 1	Oceania (17.0%)	Papua New Guinea [72341.5 (51533.9-97646.1)]; Tokelau [69505.6 (40587.1-87878.0)]; Niue [62226.9

					(51921.3-72845.6)]
YLLs	Year 2021	GBD Region (% in Top 100)	Top 2	Western Sub-Saharan Africa (12.0%)	Nigeria [54940.5 (39829.0-71395.0)]; Guinea [34697.6 (16762.7-54693.2)]; Chad [32910.2 (9203.0-60579.2)]
YLLs	Year 2021	GBD Region (% in Top 100)	Top 3	Caribbean (9.0%)	Haiti [38309.7 (20423.0-57494.8)]; Dominica [11789.0 (7890.9-17288.7)]; Guyana [10435.7 (7182.4-14800.3)]
YLLs	1980-2021		Countries behind frontier n (%)	4 (2.0%)	Tokelau [33634.2 (18416.8-36638.4)]; Niue [27904.0 (27582.0-27752.4)]; Dominica [3437.5 (3169.4-5177.5)]
YLLs	1980-2021	SDI Region (% in All FBF increased)	High (%)	0.00%	-
YLLs	1980-2021	SDI Region (% in All FBF increased)	High-middle (%)	50.00%	Niue [27904.0 (27582.0-27752.4)]; Dominica [3437.5 (3169.4-5177.5)]
YLLs	1980-2021	SDI Region (% in All FBF increased)	Middle (%)	25.00%	Tokelau [33634.2 (18416.8-36638.4)]
YLLs	1980-2021	SDI Region (% in All FBF increased)	Low-middle (%)	25.00%	Cabo Verde [287.0 (10900.4-13905.2)]
YLLs	1980-2021	SDI Region (% in All FBF increased)	Low (%)	0.00%	-
YLLs	1980-2021	GBD Region (% in All FBF increased)	Top 1	Oceania (50.0%)	Tokelau [33634.2 (18416.8-36638.4)]; Niue [27904.0 (27582.0-27752.4)]
YLLs	1980-2021	GBD Region (% in All FBF increased)	Top 2	Caribbean (25.0%)	Dominica [3437.5 (3169.4-5177.5)]
YLLs	1980-2021	GBD Region (% in	Top 3	Western	Cabo Verde [287.0 (10900.4-13905.2)]

		All FBF increased)		Sub-Saharan Africa (25.0%)	
Incidence	Year 2021		Countries behind frontier n (%)	166 (81.4%)	Pakistan [18965.7 (16153.3-21969.7)]; India [15853.7 (13424.9-18598.4)]; Kenya [14267.1 (12297.7-16450.8)]
Incidence	Year 2021	SDI Region (% in Top 100)	High (%)	6.00%	Poland [4441.8 (3933.4-5038.7)]; Brunei Darussalam [3829.6 (3196.8-4465.0)]; Singapore [2854.2 (2428.4-3318.8)]
Incidence	Year 2021	SDI Region (% in Top 100)	High-middle (%)	20.00%	Azerbaijan [8020.5 (7193.4-9110.5)]; Guam [4531.9 (3941.3-5232.4)]; Northern Mariana Islands [4249.2 (3626.7-5029.5)]
Incidence	Year 2021	SDI Region (% in Top 100)	Middle (%)	18.00%	South Africa [9524.4 (8243.6-10906.7)]; Botswana [8174.7 (6941.4-9533.4)]; Gabon [6293.6 (5053.0-7666.5)]
Incidence	Year 2021	SDI Region (% in Top 100)	Low-middle (%)	30.00%	Pakistan [18965.7 (16153.3-21969.7)]; India [15853.7 (13424.9-18598.4)]; Kenya [14267.1 (12297.7-16450.8)]
Incidence	Year 2021	SDI Region (% in Top 100)	Low (%)	26.00%	Nepal [13217.2 (10603.7-16514.7)]; Guinea [9993.5 (7740.5-12353.4)]; Sierra Leone [9700.8 (7345.9-12134.0)]
Incidence	Year 2021	GBD Region (% in Top 100)	Top 1	Oceania (17.0%)	Papua New Guinea [7191.7 (6023.6-8610.2)]; Nauru [4725.8 (3994.0-5644.5)]; Guam [4531.9 (3941.3-5232.4)]
Incidence	Year 2021	GBD Region (% in Top 100)	Top 2	Western Sub-Saharan Africa (17.0%)	Nigeria [13128.4 (11504.6-14903.1)]; Guinea [9993.5 (7740.5-12353.4)]; Sierra Leone [9700.8 (7345.9-12134.0)]
Incidence	Year 2021	GBD Region (% in Top 100)	Top 3	Eastern Sub-Saharan Africa (12.0%)	Kenya [14267.1 (12297.7-16450.8)]; Djibouti [6725.8 (5453.6-8153.3)]; United Republic of Tanzania [6441.5 (5081.9-8186.0)]
Incidence	1990-2021		Countries behind	1 (0.5%)	Somalia [111.2 (1183.3-2076.3)]; NA [NA]; NA [NA]

			frontier n (%)		
Incidence	1990-2021	SDI Region (% in All FBF increased)	High (%)	0.00%	-
Incidence	1990-2021	SDI Region (% in All FBF increased)	High-middle (%)	0.00%	-
Incidence	1990-2021	SDI Region (% in All FBF increased)	Middle (%)	0.00%	-
Incidence	1990-2021	SDI Region (% in All FBF increased)	Low-middle (%)	0.00%	-
Incidence	1990-2021	SDI Region (% in All FBF increased)	Low (%)	100.00%	Somalia [111.2 (1183.3-2076.3)]
Incidence	1990-2021	GBD Region (% in All FBF increased)	Top 1	Eastern Sub-Saharan Africa (100.0%)	Somalia [111.2 (1183.3-2076.3)]
Incidence	1990-2021	GBD Region (% in All FBF increased)	Top 2	NA (NA%)	-
Incidence	1990-2021	GBD Region (% in All FBF increased)	Top 3	NA (NA%)	-
			Lower respiratory infections|<5 years|Both		
Deaths	Year 2021		Countries behind frontier n (%)	170 (83.3%)	Nigeria [239.6 (154.6-337.4)]; Tokelau [237.3 (162.9-289.0)]; Niue [223.9 (191.7-255.5)]

Deaths	Year 2021	SDI Region (% in Top 100)	High (%)	1.00%	Kuwait [16.5 (13.1-20.0)]
Deaths	Year 2021	SDI Region (% in Top 100)	High-middle (%)	23.00%	Niue [223.9 (191.7-255.5)]; Azerbaijan [131.3 (102.8-166.5)]; Palau [47.8 (36.6-61.3)]
Deaths	Year 2021	SDI Region (% in Top 100)	Middle (%)	23.00%	Tokelau [237.3 (162.9-289.0)]; Turkmenistan [141.5 (112.8-174.0)]; Uzbekistan [118.3 (95.4-145.6)]
Deaths	Year 2021	SDI Region (% in Top 100)	Low-middle (%)	32.00%	Nigeria [239.6 (154.6-337.4)]; Lesotho [170.6 (112.0-235.7)]; Tajikistan [167.7 (111.6-235.1)]
Deaths	Year 2021	SDI Region (% in Top 100)	Low (%)	21.00%	Papua New Guinea [209.2 (145.9-291.1)]; Chad [202.2 (109.4-305.6)]; Central African Republic [181.8 (85.5-296.7)]
Deaths	Year 2021	GBD Region (% in Top 100)	Top 1	Oceania (17.0%)	Tokelau [237.3 (162.9-289.0)]; Niue [223.9 (191.7-255.5)]; Papua New Guinea [209.2 (145.9-291.1)]
Deaths	Year 2021	GBD Region (% in Top 100)	Top 2	Western Sub-Saharan Africa (11.0%)	Nigeria [239.6 (154.6-337.4)]; Chad [202.2 (109.4-305.6)]; Guinea [144.5 (72.5-230.7)]
Deaths	Year 2021	GBD Region (% in Top 100)	Top 3	Eastern Sub-Saharan Africa (10.0%)	South Sudan [175.4 (84.3-395.0)]; Comoros [79.6 (49.0-115.3)]; United Republic of Tanzania [59.8 (26.7-99.1)]
Deaths	1980-2021		Countries behind frontier n (%)	3 (1.5%)	Niue [118.9 (112.2-123.4)]; Tokelau [53.7 (30.7-39.4)]; Dominica [15.2 (16.1-18.2)]
Deaths	1980-2021	SDI Region (% in All FBF increased)	High (%)	0.00%	-
Deaths	1980-2021	SDI Region (% in All FBF increased)	High-middle (%)	66.70%	Niue [118.9 (112.2-123.4)]; Dominica [15.2 (16.1-18.2)]
Deaths	1980-2021	SDI Region (% in All FBF	Middle (%)	33.30%	Tokelau [53.7 (30.7-39.4)]

		increased)			
Deaths	1980-2021	SDI Region (% in All FBF increased)	Low-middle (%)	0.00%	-
Deaths	1980-2021	SDI Region (% in All FBF increased)	Low (%)	0.00%	-
Deaths	1980-2021	GBD Region (% in All FBF increased)	Top 1	Oceania (66.7%)	Niue [118.9 (112.2-123.4)]; Tokelau [53.7 (30.7-39.4)]
Deaths	1980-2021	GBD Region (% in All FBF increased)	Top 2	Caribbean (33.3%)	Dominica [15.2 (16.1-18.2)]
Deaths	1980-2021	GBD Region (% in All FBF increased)	Top 3	NA (NA%)	-
DALYs	Year 2021		Countries behind frontier n (%)	167 (81.9%)	Nigeria [21241.8 (13722.6-29904.0)]; Tokelau [21090.2 (14448.0-25697.5)]; Niue [19905.4 (17033.4-22713.6)]
DALYs	Year 2021	SDI Region (% in Top 100)	High (%)	1.00%	Kuwait [1474.3 (1177.2-1790.6)]
DALYs	Year 2021	SDI Region (% in Top 100)	High-middle (%)	23.00%	Niue [19905.4 (17033.4-22713.6)]; Azerbaijan [11746.3 (9205.5-14893.0)]; Palau [4271.6 (3271.0-5470.2)]
DALYs	Year 2021	SDI Region (% in Top 100)	Middle (%)	23.00%	Tokelau [21090.2 (14448.0-25697.5)]; Turkmenistan [12628.6 (10064.7-15523.6)]; Uzbekistan [10573.9 (8534.6-13007.6)]
DALYs	Year 2021	SDI Region (% in Top 100)	Low-middle (%)	32.00%	Nigeria [21241.8 (13722.6-29904.0)]; Lesotho [15260.2 (10016.3-21065.3)]; Tajikistan [14969.7 (9961.5-20978.1)]
DALYs	Year 2021	SDI Region (% in Top 100)	Low (%)	21.00%	Papua New Guinea [18657.1 (12995.5-25977.4)]; Central African Republic [16128.7 (7586.8-26286.5)]; South Sudan [15292.2 (7238.8-34778.9)]

DALYs	Year 2021	GBD Region (% in Top 100)	Top 1	Oceania (17.0%)	Tokelau [21090.2 (14448.0-25697.5)]; Niue [19905.4 (17033.4-22713.6)]; Papua New Guinea [18657.1 (12995.5-25977.4)]
DALYs	Year 2021	GBD Region (% in Top 100)	Top 2	Western Sub-Saharan Africa (11.0%)	Nigeria [21241.8 (13722.6-29904.0)]; Chad [13314.1 (5086.3-22446.4)]; Guinea [12858.3 (6465.7-20522.3)]
DALYs	Year 2021	GBD Region (% in Top 100)	Top 3	Eastern Sub-Saharan Africa (10.0%)	South Sudan [15292.2 (7238.8-34778.9)]; Comoros [7039.9 (4323.2-10241.0)]; United Republic of Tanzania [5252.1 (2318.6-8746.8)]
DALYs	1990-2021		Countries behind frontier n (%)	8 (3.9%)	Tokelau [11847.0 (8224.2-13009.0)]; Niue [11651.4 (10826.9-12041.0)]; Dominica [1340.1 (1023.2-1962.6)]
DALYs	1990-2021	SDI Region (% in All FBF increased)	High (%)	0.00%	-
DALYs	1990-2021	SDI Region (% in All FBF increased)	High-middle (%)	50.00%	Niue [11651.4 (10826.9-12041.0)]; Dominica [1340.1 (1023.2-1962.6)]; Antigua and Barbuda [264.9 (92.6-406.3)]
DALYs	1990-2021	SDI Region (% in All FBF increased)	Middle (%)	50.00%	Tokelau [11847.0 (8224.2-13009.0)]; Grenada [1207.6 (153.9-491.4)]; Saint Lucia [809.7 (2.5-697.5)]
DALYs	1990-2021	SDI Region (% in All FBF increased)	Low-middle (%)	0.00%	-
DALYs	1990-2021	SDI Region (% in All FBF increased)	Low (%)	0.00%	-
DALYs	1990-2021	GBD Region (% in All FBF increased)	Top 1	Caribbean (62.5%)	Dominica [1340.1 (1023.2-1962.6)]; Grenada [1207.6 (153.9-491.4)]; Saint Lucia [809.7 (2.5-697.5)]

DALYs	1990-2021	GBD Region (% in All FBF increased)	Top 2	Oceania (25.0%)	Tokelau [11847.0 (8224.2-13009.0)]; Niue [11651.4 (10826.9-12041.0)]
DALYs	1990-2021	GBD Region (% in All FBF increased)	Top 3	Central Europe (12.5%)	Bosnia and Herzegovina [81.2 (130.2-288.4)]
YLDs	Year 2021		Countries behind frontier n (%)	116 (56.9%)	Pakistan [12.1 (6.5-19.5)]; India [9.3 (5.1-15.3)]; Botswana [8.3 (4.6-13.7)]
YLDs	Year 2021	SDI Region (% in Top 100)	High (%)	10.00%	Taiwan (Province of China) [3.9 (1.9-6.6)]; Poland [3.1 (1.8-4.8)]; Brunei Darussalam [2.9 (1.6-4.7)]
YLDs	Year 2021	SDI Region (% in Top 100)	High-middle (%)	25.00%	Northern Mariana Islands [5.7 (3.0-9.1)]; Palau [4.4 (2.5-7.5)]; Guam [4.3 (2.4-6.9)]
YLDs	Year 2021	SDI Region (% in Top 100)	Middle (%)	21.00%	Botswana [8.3 (4.6-13.7)]; South Africa [7.6 (4.6-11.9)]; Namibia [5.0 (2.1-9.4)]
YLDs	Year 2021	SDI Region (% in Top 100)	Low-middle (%)	25.00%	Pakistan [12.1 (6.5-19.5)]; India [9.3 (5.1-15.3)]; Kenya [8.3 (4.1-14.1)]
YLDs	Year 2021	SDI Region (% in Top 100)	Low (%)	19.00%	Nepal [7.9 (2.8-15.1)]; Guinea [5.7 (1.2-12.0)]; Central African Republic [5.2 (0.6-12.5)]
YLDs	Year 2021	GBD Region (% in Top 100)	Top 1	Oceania (15.0%)	Northern Mariana Islands [5.7 (3.0-9.1)]; Papua New Guinea [4.9 (0.8-10.9)]; Palau [4.4 (2.5-7.5)]
YLDs	Year 2021	GBD Region (% in Top 100)	Top 2	Western Sub-Saharan Africa (12.0%)	Nigeria [7.3 (3.4-13.0)]; Guinea [5.7 (1.2-12.0)]; Sierra Leone [4.8 (0.7-11.0)]
YLDs	Year 2021	GBD Region (% in Top 100)	Top 3	Caribbean (10.0%)	Haiti [3.2 (0.2-7.5)]; Antigua and Barbuda [2.9 (1.5-4.8)]; Bermuda [2.8 (1.6-4.7)]
YLDs	1990-2021		Countries behind frontier n (%)	3 (1.5%)	Poland [0.7 (0.1-1.1)]; Spain [0.1 (0.7-0.7)]; Somalia [0.1 (4.6-6.5)]
YLDs	1990-2021	SDI Region (% in All FBF increased)	High (%)	33.30%	Poland [0.7 (0.1-1.1)]

YLDs	1990-2021	SDI Region (% in All FBF increased)	High-middle (%)	33.30%	Spain [0.1 (0.7-0.7)]
YLDs	1990-2021	SDI Region (% in All FBF increased)	Middle (%)	0.00%	-
YLDs	1990-2021	SDI Region (% in All FBF increased)	Low-middle (%)	0.00%	-
YLDs	1990-2021	SDI Region (% in All FBF increased)	Low (%)	33.30%	Somalia [0.1 (4.6-6.5)]
YLDs	1990-2021	GBD Region (% in All FBF increased)	Top 1	Central Europe (33.3%)	Poland [0.7 (0.1-1.1)]
YLDs	1990-2021	GBD Region (% in All FBF increased)	Top 2	Eastern Sub-Saharan Africa (33.3%)	Somalia [0.1 (4.6-6.5)]
YLDs	1990-2021	GBD Region (% in All FBF increased)	Top 3	Western Europe (33.3%)	Spain [0.1 (0.7-0.7)]
YLLs	Year 2021		Countries behind frontier n (%)	170 (83.3%)	Nigeria [21238.4 (13720.2-29898.0)]; Tokelau [21086.9 (14444.8-25694.7)]; Niue [19901.3 (17030.1-22709.3)]
YLLs	Year 2021	SDI Region (% in Top 100)	High (%)	1.00%	Kuwait [1471.7 (1174.5-1787.9)]
YLLs	Year 2021	SDI Region (% in Top 100)	High-middle (%)	23.00%	Niue [19901.3 (17030.1-22709.3)]; Azerbaijan [11742.3 (9201.4-14889.4)]; Palau [4268.2 (3267.7-5467.1)]
YLLs	Year 2021	SDI Region (% in Top 100)	Middle (%)	23.00%	Tokelau [21086.9 (14444.8-25694.7)]; Turkmenistan [12624.1 (10059.9-15519.7)]; Uzbekistan [10572.3 (8532.5-13007.1)]

YLLs	Year 2021	SDI Region (% in Top 100)	Low-middle (%)	32.00%	Nigeria [21238.4 (13720.2-29898.0)]; Lesotho [15254.1 (10007.6-21058.4)]; Tajikistan [14973.3 (9964.4-20983.1)]
YLLs	Year 2021	SDI Region (% in Top 100)	Low (%)	21.00%	Papua New Guinea [18710.0 (13043.9-26033.8)]; Chad [17836.3 (9593.9-26981.3)]; Central African Republic [16168.5 (7624.5-26325.9)]
YLLs	Year 2021	GBD Region (% in Top 100)	Top 1	Oceania (17.0%)	Tokelau [21086.9 (14444.8-25694.7)]; Niue [19901.3 (17030.1-22709.3)]; Papua New Guinea [18710.0 (13043.9-26033.8)]
YLLs	Year 2021	GBD Region (% in Top 100)	Top 2	Western Sub-Saharan Africa (11.0%)	Nigeria [21238.4 (13720.2-29898.0)]; Chad [17836.3 (9593.9-26981.3)]; Guinea [12852.7 (6459.3-20514.5)]
YLLs	Year 2021	GBD Region (% in Top 100)	Top 3	Eastern Sub-Saharan Africa (10.0%)	South Sudan [15514.6 (7444.6-35020.4)]; Comoros [7061.5 (4342.3-10263.4)]; United Republic of Tanzania [5270.2 (2332.4-8769.6)]
YLLs	1980-2021		Countries behind frontier n (%)	3 (1.5%)	Niue [10539.0 (9940.0-10941.2)]; Tokelau [4778.4 (2737.6-3485.6)]; Dominica [1359.7 (1439.5-1624.0)]
YLLs	1980-2021	SDI Region (% in All FBF increased)	High (%)	0.00%	-
YLLs	1980-2021	SDI Region (% in All FBF increased)	High-middle (%)	66.70%	Niue [10539.0 (9940.0-10941.2)]; Dominica [1359.7 (1439.5-1624.0)]
YLLs	1980-2021	SDI Region (% in All FBF increased)	Middle (%)	33.30%	Tokelau [4778.4 (2737.6-3485.6)]
YLLs	1980-2021	SDI Region (% in All FBF increased)	Low-middle (%)	0.00%	-

YLLs	1980-2021	SDI Region (% in All FBF increased)	Low (%)	0.00%	-
YLLs	1980-2021	GBD Region (% in All FBF increased)	Top 1	Oceania (66.7%)	Niue [10539.0 (9940.0-10941.2)]; Tokelau [4778.4 (2737.6-3485.6)]
YLLs	1980-2021	GBD Region (% in All FBF increased)	Top 2	Caribbean (33.3%)	Dominica [1359.7 (1439.5-1624.0)]
YLLs	1980-2021	GBD Region (% in All FBF increased)	Top 3	NA (NA%)	-
Incidence	Year 2021		Countries behind frontier n (%)	164 (80.4%)	Pakistan [8689.2 (7127.1-10361.9)]; India [6682.4 (5474.6-8261.2)]; Botswana [6093.2 (4986.5-7436.2)]
Incidence	Year 2021	SDI Region (% in Top 100)	High (%)	11.00%	Taiwan (Province of China) [3040.8 (2413.0-3857.6)]; Bermuda [2124.3 (1686.4-2611.4)]; Lithuania [2122.6 (1566.4-2814.8)]
Incidence	Year 2021	SDI Region (% in Top 100)	High-middle (%)	25.00%	Northern Mariana Islands [4146.9 (3379.8-4947.8)]; Palau [3302.2 (2757.5-3998.4)]; Guam [3215.9 (2705.9-3802.7)]
Incidence	Year 2021	SDI Region (% in Top 100)	Middle (%)	21.00%	Botswana [6093.2 (4986.5-7436.2)]; South Africa [5658.9 (4843.4-6627.8)]; Namibia [3813.7 (3000.5-4782.4)]
Incidence	Year 2021	SDI Region (% in Top 100)	Low-middle (%)	24.00%	Pakistan [8689.2 (7127.1-10361.9)]; India [6682.4 (5474.6-8261.2)]; Kenya [5856.8 (4764.1-7058.3)]
Incidence	Year 2021	SDI Region (% in Top 100)	Low (%)	19.00%	Nepal [5562.2 (4139.1-7176.8)]; Guinea [4218.6 (3048.7-5491.8)]; Central African Republic [3810.0 (2352.5-5471.8)]
Incidence	Year 2021	GBD Region (% in Top 100)	Top 1	Oceania (15.0%)	Northern Mariana Islands [4146.9 (3379.8-4947.8)]; Papua New Guinea [3717.5 (2612.8-5016.7)]; Nauru [3381.6 (2649.6-4139.0)]

Incidence	Year 2021	GBD Region (% in Top 100)	Top 2	Western Sub-Saharan Africa (11.0%)	Nigeria [5428.1 (4488.8-6448.2)]; Guinea [4218.6 (3048.7-5491.8)]; Sierra Leone [3559.9 (2432.7-4769.1)]
Incidence	Year 2021	GBD Region (% in Top 100)	Top 3	Caribbean (10.0%)	Haiti [2320.1 (1561.0-3166.0)]; Bermuda [2124.3 (1686.4-2611.4)]; Antigua and Barbuda [2120.6 (1732.3-2584.5)]
Incidence	1990-2021		Countries behind frontier n (%)	3 (1.5%)	Poland [420.1 (255.7-548.8)]; Spain [74.3 (89.2-239.2)]; Somalia [39.9 (1435.8-1942.0)]
Incidence	1990-2021	SDI Region (% in All FBF increased)	High (%)	33.30%	Poland [420.1 (255.7-548.8)]
Incidence	1990-2021	SDI Region (% in All FBF increased)	High-middle (%)	33.30%	Spain [74.3 (89.2-239.2)]
Incidence	1990-2021	SDI Region (% in All FBF increased)	Middle (%)	0.00%	-
Incidence	1990-2021	SDI Region (% in All FBF increased)	Low-middle (%)	0.00%	-
Incidence	1990-2021	SDI Region (% in All FBF increased)	Low (%)	33.30%	Somalia [39.9 (1435.8-1942.0)]
Incidence	1990-2021	GBD Region (% in All FBF increased)	Top 1	Central Europe (33.3%)	Poland [420.1 (255.7-548.8)]
Incidence	1990-2021	GBD Region (% in All FBF increased)	Top 2	Eastern Sub-Saharan Africa (33.3%)	Somalia [39.9 (1435.8-1942.0)]

Incidence	1990-2021	GBD Region (% in All FBF increased)	Top 3	Western Europe (33.3%)	Spain [74.3 (89.2-239.2)]
			Lower respiratory infections|<20 years|Both		
Deaths	Year 2021		Countries behind frontier n (%)	173 (84.8%)	Chad [79.0 (46.8-115.1)]; Nigeria [74.0 (48.1-104.2)]; Papua New Guinea [68.9 (48.8-94.1)]
Deaths	Year 2021	SDI Region (% in Top 100)	High (%)	1.00%	Kuwait [5.5 (4.5-6.6)]
Deaths	Year 2021	SDI Region (% in Top 100)	High-middle (%)	22.00%	Niue [60.4 (53.3-68.6)]; Azerbaijan [35.0 (27.9-43.6)]; Palau [13.6 (10.6-17.0)]
Deaths	Year 2021	SDI Region (% in Top 100)	Middle (%)	23.00%	Tokelau [56.4 (39.7-68.4)]; Turkmenistan [43.2 (35.1-52.3)]; Uzbekistan [39.8 (32.6-48.4)]
Deaths	Year 2021	SDI Region (% in Top 100)	Low-middle (%)	31.00%	Nigeria [74.0 (48.1-104.2)]; Tajikistan [55.5 (37.9-76.3)]; Lesotho [48.1 (33.9-64.7)]
Deaths	Year 2021	SDI Region (% in Top 100)	Low (%)	23.00%	Chad [79.0 (46.8-115.1)]; Papua New Guinea [68.9 (48.8-94.1)]; Central African Republic [53.5 (25.1-87.7)]
Deaths	Year 2021	GBD Region (% in Top 100)	Top 1	Oceania (17.0%)	Papua New Guinea [68.9 (48.8-94.1)]; Niue [60.4 (53.3-68.6)]; Tokelau [56.4 (39.7-68.4)]
Deaths	Year 2021	GBD Region (% in Top 100)	Top 2	Western Sub-Saharan Africa (13.0%)	Chad [79.0 (46.8-115.1)]; Nigeria [74.0 (48.1-104.2)]; Guinea [45.3 (22.3-72.1)]
Deaths	Year 2021	GBD Region (% in Top 100)	Top 3	Eastern Sub-Saharan Africa (11.0%)	South Sudan [52.6 (25.2-116.2)]; Comoros [24.6 (16.4-34.7)]; United Republic of Tanzania [21.9 (12.1-34.3)]
Deaths	1980-2021		Countries behind frontier n (%)	4 (2.0%)	Niue [30.9 (28.6-33.9)]; Tokelau [4.7 (3.6-4.6)]; Dominica [3.5 (3.6-4.0)]

Deaths	1980-2021	SDI Region (% in All FBF increased)	High (%)	0.00%	-
Deaths	1980-2021	SDI Region (% in All FBF increased)	High-middle (%)	50.00%	Niue [30.9 (28.6-33.9)]; Dominica [3.5 (3.6-4.0)]
Deaths	1980-2021	SDI Region (% in All FBF increased)	Middle (%)	50.00%	Tokelau [4.7 (3.6-4.6)]; Saint Lucia [2.0 (1.8-2.2)]
Deaths	1980-2021	SDI Region (% in All FBF increased)	Low-middle (%)	0.00%	-
Deaths	1980-2021	SDI Region (% in All FBF increased)	Low (%)	0.00%	-
Deaths	1980-2021	GBD Region (% in All FBF increased)	Top 1	Caribbean (50.0%)	Dominica [3.5 (3.6-4.0)]; Saint Lucia [2.0 (1.8-2.2)]
Deaths	1980-2021	GBD Region (% in All FBF increased)	Top 2	Oceania (50.0%)	Niue [30.9 (28.6-33.9)]; Tokelau [4.7 (3.6-4.6)]
Deaths	1980-2021	GBD Region (% in All FBF increased)	Top 3	NA (NA%)	-
DALYs	Year 2021		Countries behind frontier n (%)	173 (84.8%)	Nigeria [6534.9 (4260.8-9189.4)]; Papua New Guinea [6151.5 (4363.8-8409.7)]; Niue [5266.1 (4628.3-5977.9)]
DALYs	Year 2021	SDI Region (% in Top 100)	High (%)	1.00%	Kuwait [474.1 (388.0-569.2)]
DALYs	Year 2021	SDI Region (% in Top 100)	High-middle (%)	23.00%	Niue [5266.1 (4628.3-5977.9)]; Azerbaijan [3096.0 (2464.4-3861.3)]; Palau [1163.2 (907.1-1462.2)]

DALYs	Year 2021	SDI Region (% in Top 100)	Middle (%)	22.00%	Tokelau [4905.0 (3436.3-5962.1)]; Turkmenistan [3818.3 (3093.3-4627.8)]; Uzbekistan [3529.4 (2885.8-4293.1)]
DALYs	Year 2021	SDI Region (% in Top 100)	Low-middle (%)	31.00%	Nigeria [6534.9 (4260.8-9189.4)]; Tajikistan [4920.2 (3352.7-6769.5)]; Lesotho [4218.6 (2952.6-5698.0)]
DALYs	Year 2021	SDI Region (% in Top 100)	Low (%)	23.00%	Papua New Guinea [6151.5 (4363.8-8409.7)]; Central African Republic [4732.1 (2215.1-7767.2)]; Chad [4691.9 (1848.4-7887.1)]
DALYs	Year 2021	GBD Region (% in Top 100)	Top 1	Oceania (17.0%)	Papua New Guinea [6151.5 (4363.8-8409.7)]; Niue [5266.1 (4628.3-5977.9)]; Tokelau [4905.0 (3436.3-5962.1)]
DALYs	Year 2021	GBD Region (% in Top 100)	Top 2	Western Sub-Saharan Africa (13.0%)	Nigeria [6534.9 (4260.8-9189.4)]; Chad [4691.9 (1848.4-7887.1)]; Guinea [4004.5 (1977.1-6379.1)]
DALYs	Year 2021	GBD Region (% in Top 100)	Top 3	Eastern Sub-Saharan Africa (11.0%)	South Sudan [4459.2 (2055.5-10101.9)]; Comoros [2147.2 (1431.2-3038.7)]; United Republic of Tanzania [1890.0 (1017.8-2984.1)]
DALYs	1990-2021		Countries behind frontier n (%)	9 (4.4%)	Niue [2863.3 (2790.2-2890.3)]; Tokelau [2217.3 (1590.9-2310.3)]; Grenada [251.5 (23.8-61.0)]
DALYs	1990-2021	SDI Region (% in All FBF increased)	High (%)	0.00%	-
DALYs	1990-2021	SDI Region (% in All FBF increased)	High-middle (%)	55.60%	Niue [2863.3 (2790.2-2890.3)]; Dominica [148.8 (138.1-201.3)]; Antigua and Barbuda [28.0 (31.0-76.8)]
DALYs	1990-2021	SDI Region (% in All FBF increased)	Middle (%)	44.40%	Tokelau [2217.3 (1590.9-2310.3)]; Grenada [251.5 (23.8-61.0)]; Saint Lucia [214.5 (14.6-166.5)]
DALYs	1990-2021	SDI Region (% in	Low-middle (%)	0.00%	-

		All FBF increased)			
DALYs	1990-2021	SDI Region (% in All FBF increased)	Low (%)	0.00%	-
DALYs	1990-2021	GBD Region (% in All FBF increased)	Top 1	Caribbean (55.6%)	Grenada [251.5 (23.8-61.0)]; Saint Lucia [214.5 (14.6-166.5)]; Saint Vincent and the Grenadines [177.9 (69.3-80.0)]
DALYs	1990-2021	GBD Region (% in All FBF increased)	Top 2	Oceania (22.2%)	Niue [2863.3 (2790.2-2890.3)]; Tokelau [2217.3 (1590.9-2310.3)]
DALYs	1990-2021	GBD Region (% in All FBF increased)	Top 3	Central Europe (11.1%)	Bosnia and Herzegovina [28.0 (33.9-76.8)]
YLDs	Year 2021		Countries behind frontier n (%)	136 (66.7%)	Pakistan [7.0 (3.8-10.9)]; Nepal [5.3 (2.2-9.3)]; India [5.2 (2.8-8.0)]
YLDs	Year 2021	SDI Region (% in Top 100)	High (%)	11.00%	Taiwan (Province of China) [2.6 (1.4-4.2)]; Lithuania [1.7 (0.9-2.8)]; Singapore [1.6 (0.9-2.5)]
YLDs	Year 2021	SDI Region (% in Top 100)	High-middle (%)	24.00%	China [2.6 (1.5-4.2)]; Northern Mariana Islands [2.3 (1.3-3.6)]; Azerbaijan [2.3 (1.3-3.7)]
YLDs	Year 2021	SDI Region (% in Top 100)	Middle (%)	20.00%	Botswana [4.5 (2.5-7.3)]; South Africa [4.2 (2.6-6.2)]; Namibia [3.4 (1.8-5.4)]
YLDs	Year 2021	SDI Region (% in Top 100)	Low-middle (%)	24.00%	Pakistan [7.0 (3.8-10.9)]; India [5.2 (2.8-8.0)]; Kenya [5.1 (2.7-8.0)]
YLDs	Year 2021	SDI Region (% in Top 100)	Low (%)	21.00%	Nepal [5.3 (2.2-9.3)]; Eritrea [3.1 (0.7-6.3)]; United Republic of Tanzania [3.0 (1.0-5.5)]
YLDs	Year 2021	GBD Region (% in Top 100)	Top 1	Oceania (14.0%)	Nauru [2.7 (1.4-4.5)]; Northern Mariana Islands [2.3 (1.3-3.6)]; Fiji [2.2 (1.2-3.7)]
YLDs	Year 2021	GBD Region (% in Top 100)	Top 2	Western Sub-Saharan Africa	Nigeria [3.4 (1.5-5.9)]; Guinea [3.0 (0.4-6.2)]; Chad [2.8 (0.0-6.4)]

				(14.0%)	
YLDs	Year 2021	GBD Region (% in Top 100)	Top 3	Eastern Sub-Saharan Africa (13.0%)	Kenya [5.1 (2.7-8.0)]; Djibouti [3.5 (1.5-6.2)]; Eritrea [3.1 (0.7-6.3)]
YLDs	1990-2021		Countries behind frontier n (%)	1 (0.5%)	Spain [0.1 (0.1-0.2)]; NA [NA]; NA [NA]
YLDs	1990-2021	SDI Region (% in All FBF increased)	High (%)	0.00%	-
YLDs	1990-2021	SDI Region (% in All FBF increased)	High-middle (%)	100.00%	Spain [0.1 (0.1-0.2)]
YLDs	1990-2021	SDI Region (% in All FBF increased)	Middle (%)	0.00%	-
YLDs	1990-2021	SDI Region (% in All FBF increased)	Low-middle (%)	0.00%	-
YLDs	1990-2021	SDI Region (% in All FBF increased)	Low (%)	0.00%	-
YLDs	1990-2021	GBD Region (% in All FBF increased)	Top 1	Western Europe (100.0%)	Spain [0.1 (0.1-0.2)]
YLDs	1990-2021	GBD Region (% in All FBF increased)	Top 2	NA (NA%)	-
YLDs	1990-2021	GBD Region (% in All FBF increased)	Top 3	NA (NA%)	-

YLLs	Year 2021		Countries behind frontier n (%)	172 (84.3%)	Chad [6980.9 (4137.1-10176.0)]; Nigeria [6535.0 (4261.5-9189.0)]; Papua New Guinea [6167.8 (4377.6-8428.2)]
YLLs	Year 2021	SDI Region (% in Top 100)	High (%)	1.00%	Kuwait [472.6 (386.6-567.2)]
YLLs	Year 2021	SDI Region (% in Top 100)	High-middle (%)	22.00%	Niue [5264.1 (4626.9-5975.4)]; Azerbaijan [3093.7 (2461.7-3859.1)]; Palau [1161.1 (904.9-1460.2)]
YLLs	Year 2021	SDI Region (% in Top 100)	Middle (%)	23.00%	Tokelau [4903.4 (3435.0-5960.6)]; Turkmenistan [3816.1 (3091.1-4625.5)]; Uzbekistan [3527.9 (2883.3-4292.4)]
YLLs	Year 2021	SDI Region (% in Top 100)	Low-middle (%)	31.00%	Nigeria [6535.0 (4261.5-9189.0)]; Tajikistan [4921.1 (3352.7-6770.4)]; Lesotho [4217.0 (2950.9-5696.4)]
YLLs	Year 2021	SDI Region (% in Top 100)	Low (%)	23.00%	Chad [6980.9 (4137.1-10176.0)]; Papua New Guinea [6167.8 (4377.6-8428.2)]; Central African Republic [4734.0 (2217.2-7766.9)]
YLLs	Year 2021	GBD Region (% in Top 100)	Top 1	Oceania (17.0%)	Papua New Guinea [6167.8 (4377.6-8428.2)]; Niue [5264.1 (4626.9-5975.4)]; Tokelau [4903.4 (3435.0-5960.6)]
YLLs	Year 2021	GBD Region (% in Top 100)	Top 2	Western Sub-Saharan Africa (13.0%)	Chad [6980.9 (4137.1-10176.0)]; Nigeria [6535.0 (4261.5-9189.0)]; Guinea [4004.1 (1975.9-6378.7)]
YLLs	Year 2021	GBD Region (% in Top 100)	Top 3	Eastern Sub-Saharan Africa (11.0%)	South Sudan [4603.1 (2187.6-10257.7)]; Comoros [2149.2 (1433.7-3040.3)]; United Republic of Tanzania [1921.5 (1046.8-3018.5)]
YLLs	1980-2021		Countries behind frontier n (%)	4 (2.0%)	Niue [2669.3 (2452.1-2926.5)]; Tokelau [353.1 (350.4-380.5)]; Dominica [300.5 (310.8-350.0)]
YLLs	1980-2021	SDI Region (% in All FBF increased)	High (%)	0.00%	-

YLLs	1980-2021	SDI Region (% in All FBF increased)	High-middle (%)	50.00%	Niue [2669.3 (2452.1-2926.5)]; Dominica [300.5 (310.8-350.0)]
YLLs	1980-2021	SDI Region (% in All FBF increased)	Middle (%)	50.00%	Tokelau [353.1 (350.4-380.5)]; Saint Lucia [141.9 (163.0-195.7)]
YLLs	1980-2021	SDI Region (% in All FBF increased)	Low-middle (%)	0.00%	-
YLLs	1980-2021	SDI Region (% in All FBF increased)	Low (%)	0.00%	-
YLLs	1980-2021	GBD Region (% in All FBF increased)	Top 1	Caribbean (50.0%)	Dominica [300.5 (310.8-350.0)]; Saint Lucia [141.9 (163.0-195.7)]
YLLs	1980-2021	GBD Region (% in All FBF increased)	Top 2	Oceania (50.0%)	Niue [2669.3 (2452.1-2926.5)]; Tokelau [353.1 (350.4-380.5)]
YLLs	1980-2021	GBD Region (% in All FBF increased)	Top 3	NA (NA%)	-
Incidence	Year 2021		Countries behind frontier n (%)	175 (85.8%)	Pakistan [4939.0 (4237.2-5846.0)]; India [3748.9 (3134.5-4477.4)]; Nepal [3707.4 (2942.5-4658.1)]
Incidence	Year 2021	SDI Region (% in Top 100)	High (%)	10.00%	Taiwan (Province of China) [1825.0 (1491.1-2247.7)]; Lithuania [1224.5 (973.0-1558.5)]; Estonia [1034.8 (822.7-1308.5)]
Incidence	Year 2021	SDI Region (% in Top 100)	High-middle (%)	23.00%	China [1836.8 (1490.5-2240.4)]; Northern Mariana Islands [1666.3 (1412.5-1945.4)]; Azerbaijan [1633.2 (1413.5-1871.9)]
Incidence	Year 2021	SDI Region (% in Top 100)	Middle (%)	21.00%	Botswana [3215.8 (2720.7-3801.3)]; South Africa [2923.0 (2554.9-3399.8)]; Namibia [2437.2

					(2085.5-2902.0)]
Incidence	Year 2021	SDI Region (% in Top 100)	Low-middle (%)	25.00%	Pakistan [4939.0 (4237.2-5846.0)]; India [3748.9 (3134.5-4477.4)]; Kenya [3669.0 (3162.0-4262.9)]
Incidence	Year 2021	SDI Region (% in Top 100)	Low (%)	21.00%	Nepal [3707.4 (2942.5-4658.1)]; Eritrea [2298.5 (1720.6-2961.8)]; United Republic of Tanzania [2217.2 (1708.8-2790.7)]
Incidence	Year 2021	GBD Region (% in Top 100)	Top 1	Oceania (15.0%)	Nauru [1974.7 (1669.7-2305.6)]; Northern Mariana Islands [1666.3 (1412.5-1945.4)]; Fiji [1593.3 (1350.6-1867.9)]
Incidence	Year 2021	GBD Region (% in Top 100)	Top 2	Western Sub-Saharan Africa (14.0%)	Nigeria [2508.2 (2111.7-2973.7)]; Guinea [2202.1 (1701.5-2811.5)]; Sierra Leone [2096.0 (1579.9-2680.3)]
Incidence	Year 2021	GBD Region (% in Top 100)	Top 3	Eastern Sub-Saharan Africa (13.0%)	Kenya [3669.0 (3162.0-4262.9)]; Djibouti [2566.3 (2112.0-3137.5)]; Eritrea [2298.5 (1720.6-2961.8)]
Incidence	1990-2021		Countries behind frontier n (%)	1 (0.5%)	Spain [29.3 (0.9-67.3)]; NA [NA]; NA [NA]
Incidence	1990-2021	SDI Region (% in All FBF increased)	High (%)	0.00%	-
Incidence	1990-2021	SDI Region (% in All FBF increased)	High-middle (%)	100.00%	Spain [29.3 (0.9-67.3)]
Incidence	1990-2021	SDI Region (% in All FBF increased)	Middle (%)	0.00%	-
Incidence	1990-2021	SDI Region (% in All FBF increased)	Low-middle (%)	0.00%	-

Incidence	1990-2021	SDI Region (% in All FBF increased)	Low (%)	0.00%	-
Incidence	1990-2021	GBD Region (% in All FBF increased)	Top 1	Western Europe (100.0%)	Spain [29.3 (0.9-67.3)]
Incidence	1990-2021	GBD Region (% in All FBF increased)	Top 2	NA (NA%)	-
Incidence	1990-2021	GBD Region (% in All FBF increased)	Top 3	NA (NA%)	-
